# Supplementary material for: Skeletal Ni electrode-catalyzed C-O cleavage of diaryl ethers entails direct elimination via benzyne intermediates
Source: Nat Commun. 2022 Apr 19;13:2050. doi: 10.1038/s41467-022-29555-3 (PMC9018776; doi:10.1038/s41467-022-29555-3)
Supplement: Supplementary file 1 — SUPPLEMENTARY INFO [file 41467_2022_29555_MOESM1_ESM.pdf]

Supplementary information

Skeletal Ni Electrode-Catalyzed C-O Cleavage of Diaryl Ethers Entails Direct Elimination via Benzyne Intermediates

Yuting Zhou,*[,1] Grace E. Klinger,[1,2] Eric L. Hegg,[2] Christopher M. Saffron,[3,4] James E. Jackson*[,1]

Departments of [1]Chemistry, [2]Biochemistry & Molecular Biology, [3]Biosystems & Agricultural Engineering, & [4]Chemical Engineering & Material Science, Michigan State University, East Lansing, Michigan 48824, United States

Table of Contents

General Experimental Details S2
Preparation of Ni Plating Solution, pH 7 Phosphate Buffer and pH 8 Borate Buffer S3
Preparation of Skeletal Nickel Cathode Electrode S3
Preparation of Water splitting CoP Anode S4
General Procedure of Electro-Catalytic Hydrogenation/Hydrogenolysis (ECH) S4
Volatile Product Evaporation Accounts for the Main Losses in Mass Balance S5
General Experimental Procedure for H/D Isotope Exchange Experiments S6
MS Fitting Method for analysis of H/D Labeling Experiment S6
Fitting Results: % D_n Incorporation During H/D Exchange of Diphenyl Ether S9
H/D Exchange Analysis: Mass and NMR Spectra of Diphenyl Ether S10
Fitting Results: % D_n Incorporation During H/D Exchange of Diphenyl Ether with Applied Current S12
Full ECH Time Courses for Diphenyl Ether, 4-Phenoxytoluene, 4-Phenoxyanisole and 4-Phenoxyphenol S13
Fitting Results: % D_n Incorporation During H/D Exchange of Methoxylated Diphenyl Ether S14
H/D Exchange Analysis: Mass and NMR Spectra of Methoxylated Diphenyl Ether S15
Fitting Results: % D_n Incorporation During H/D Exchange of Methylated Diphenyl Ether S17
H/D Exchange Analysis: Mass and NMR Spectra of Methylated Diphenyl Ether S18
Fitting Results: % D_n Incorporation During H/D Exchange of Hydroxylated Diphenyl Ether S20
H/D Exchange Analysis: Mass and NMR Spectra of Hydroxylated Diphenyl Ether S21
Full ECH Time Courses for para, meta and ortho Methoxylated Diphenyl Ethers S23
Full ECH Time Courses for para, meta and ortho Methylated Diphenyl Ethers S24
Full ECH Time Courses for para, meta and ortho Hydroxylated Diphenyl Ethers S25
Cleavage Regioselectivity of Asymmetric Diphenyl Ethers S26
Cleavage Regioselectivity of Methylated Diphenyl Ether Under Different Currents/Potentials S27
Hydroxylated DPE: The Outlier, Regioselectivity Is Not Altered by OH Position S28
Fitting Results: % D_n Incorporation During H/D Exchange of meta and ortho Methylated Diphenyl Ether S29
H/D Exchange Analysis Mass and NMR Spectra of meta and ortho Methylated Diphenyl Ether S30
Fitting Results: % D_n Incorporation During H/D Exchange of meta Methoxylated Diphenyl Ether S34

H/D Exchange Analysis: Mass and NMR Spectra of meta Methoxylated Diphenyl Ether S35
Fitting Results: % D_n Incorporation During H/D Exchange of meta and ortho Hydroxylated Diphenyl Ether S37
H/D Exchange Analysis: Mass and NMR Spectra of meta and ortho Hydroxylated Diphenyl Ether...... S38
Full ECH Time Courses for Diphenyl Ether with Different Organic Co-Solvents S43
Full ECH Time Courses for Hydroxylated Diphenyl Ether with Different Organic Co-Solvents S44
Plotted Comparison of Acetone Inhibitory Effects: DPE vs. HO-DPE S44
Extraction Efficiency of Other Aromatic Products S45
ECH of Diphenyl Ether Using Reticulated Vitreous Carbon (RVC) Electrode S45
Full ECH Time Courses for 1-(4-phenoxyphenyl)ethan-1-one S46
Full ECH Time Courses for 1-(4-phenoxyphenyl)ethan-1-ol S46
Full ECH Time Courses for 4-phenoxybenzonitrile S47
Full ECH Time Courses for 4-phenoxybenzaldehyde S47
Full ECH Time Courses for 1-fluoro-4-phenoxybenzene S48
Full ECH Time Courses for 1-phenoxy-4-(trifluoromethyl)benzene S48
Full ECH Time Courses for 1-ethyl-4-phenoxybenzene S49
Full ECH Time Courses of 1-phenoxy-4-(trifluoromethyl)benzene with Higher Current S49
Full ECH Time Courses of 4,4'-oxybis(methoxybenzene) S50
Kinetic Cleavage Rate Comparison of Different Functionalized Diphenyl Ethers S51
Synthesis Procedures of Substituted Diphenyl Ethers S52
NMR Spectra of Synthesized Diphenyl Ethers S55
References S70

General Experimental Details

All purchased chemicals were tested for purity using ^{1}H NMR prior to use. O-cresol ≥98%, m-cresol 99%, p-cresol 99%, nickel(II) chloride hexahydrate 99.3% (metals basis), 1,2-cyclohexanediol (cis+trans) 98%, phenol 99+%, 4-methoxyphenol (98+%), and 4-methylcyclohexanol (cis+trans) 98% were purchased from Alfa Aesar; 4-fluorophenol 99%, α,α,α-trifluorotoluene anhydrous ≥99%, 4-bromobenzotrifluoride 99%, 4-(trifluoromethyl)phenol 97%, 4-bromoanisole ≥99%, 4-bromobenzaldehyde 99%, 4-bromobenzonitrile 99%, 4-bromoacetophenone 98%, cyclohexanemethanol ≥99%, potassium tetraborate tetrahydrate ≥99.5%, benzyl alcohol anhydrous 99.8%, benzonitrile 99%, ethylbenzene anhydrous 99.8%, acetophenone 99%, 1-phenylethanol 98%, 1-cyclohexylethanol 97%, cyclohexanol 99%, toluene ≥99.5%, 4-ethylphenol 99%, 3-methoxyphenol 96%, anisole 99.7%, benzene ≥99%, and iodobenzene 98% were purchased from Sigma-Aldrich; guaiacol 99+% was purchased from Acros Organics;

Nafion membrane 117 was purchased from Fuel Cell Store; aluminum-nickel alloy purum (Al:Ni 50:50 by weight) was purchased from Sigma-Aldrich; nickel strip 22x127 mm was purchased from Science Company; boric acid (Granular) 99.5% was purchased from J. T. Baker; potassium carbonate, sodium hydride (60% in mineral oil) and potassium phosphate monobasic (KH_2PO_4) were purchased from Jade Scientific; potassium phosphate dibasic (K_2HPO_4) and ammonium chloride ≥99.5% were purchased from Columbus Chemical Industries, Inc; ammonium hydroxide (28~30% NH_3) was purchased from Mallinckrodt Chemicals; sodium hydroxide, cesium carbonate 99% and copper(I) iodide 98% were purchased from Sigma-Aldrich; N,N-dimethylglycine >98% was purchased from Tokyo Chemical Industry Co.; pentane HPLC grade 99.7% was purchased from Fisher Chemical; isopropyl alcohol ≥99.5%, dichloromethane ≥99.5% (40-150 ppm amylene as stabilizer), ethanol 99.8%, acetone ≥99.5%, hexanes ≥99% and 1,4-dioxane ≥99.0% were purchased from Sigma-Aldrich. All of the solvents were purchased

from commercial suppliers and used without further purification. Duocel® reticulated vitreous carbon foam (100 pores per inch) was purchased from ERG materials & aerospace.

Nuclear magnetic resonance (NMR) spectra were acquired on Agilent 500/54 premium shielded instruments at the Michigan State University Max T. Rogers NMR facility. ^{1}H and ^{13}C chemical shifts are reported in ppm downfield of tetramethylsilane and referenced to residual solvent peak ($CHCl_3$; δH = 7.26 ppm). Multiplicities are reported using the following abbreviations: s = singlet, d = doublet, t = triplet, q = quartet, m = multiplet, br = broad resonance. GC-MS analyses were performed on an Agilent 7890A GC/ single quadrupole mass spectrometer with 5975C inert XL MSD (Agilent, Santa Clara, CA) equipped with Agilent J&W VF-5ms column (30 m x 0.25 mm x 0.25 mm) (Agilent, Santa Clara, CA). GC-MS yields were determined based on external standards with prepared concentrations (0.125 mM - 4 mM) to form a 6-point calibration curve for each compound of interest. Each GC-MS analysis sequence included a freshly prepared external standard sequence.

Column chromatographic purification of reaction mixtures was performed using Silicycle (Quebec City, Canada) SiliaFlash P60 silica gel (40-63 μm). Thin layer chromatography (TLC) analysis of reaction mixtures was performed using Sigma Aldrich (St. Louis, MO) plastic silica gel 60 F-254 plates and the bands were visualized using UV light (254 nm).

Preparation of Plating Solution, pH 8 Borate Buffer and pH 7 Phosphate Buffer[1, 2]

Plating Solution:
To a 1 L volumetric flask, 213 g of $NiCl_2{\cdot}6H_2O$ and 30 g of NH_4Cl were added; 500 mL of deionized water was then added, followed by 200 mL of NH_4OH solution (28~30% NH_3) to keep the plating bath pH between 9-10. Deionized water was then added to make a total volume of 1 L plating solution.

pH 8 Borate Buffer (0.1 M):
To achieve an ultimate experimental concentration of 0.1 M considering the intended co-solvent fraction, the appropriate amount of DI water was measured by a graduated cylinder (e.g. for the standard conditions, 333 mL of water was measured for a 2:1 water:co-solvent buffer containing 3.00 g of H_3BO_3 (0.097 M) and 1.00 g of $K_2B_4O_7{\cdot}4H_2O$ (0.0066 M)). Depending on measured pH, if needed, KOH was added to adjust pH to 8. Upon assembly of the electrode chemical apparatus, the appropriate corresponding volume of co-solvent was added as described in the reaction procedure (page S4).

pH 7 Phosphate Buffer (0.1 M):
To a 1 L volumetric flask, 5.30 g of KH_2PO_4 (0.039 M) and 10.8 g of K_2HPO_4 (0.062 M) was added, deionized water was added to make a buffer solution with 1 L total volume.

Preparation of Skeletal Nickel Cathode Electrode Following Previously Reported Procedure[2]

To achieve maximum surface area that contains most potential active sites, a square piece of stainless-steel wire mesh was subjected to nickel electroplating to trap the aluminum-nickel powder. About 1.5 g of aluminum-nickel (1:1) alloy purum was placed in 50 ml of the above nickel-ammonia plating solution with stirring at 350 rpm. A square of stainless steel 314 screen (50 mesh, 2.5 x 2.5 cm) that connected to the cathode was positioned facing parallel to a plain nickel bar as a sacrificial anode. Application of electrical current effects direct deposition of nickel on the mesh, trapping the nickel-aluminum powder during the reduction of nickel ions. The plating current was maintained at 0.6 A for approximately 1~2 hours, and the cathode was turned 180° every 30 minutes to ensure even deposition of the nickel-aluminum particles on

both sides. The plated skeletal nickel cathode was then activated in 30 wt% NaOH solution at 75 °C for 6 hours to etch out the aluminum. After the etching process, the activated skeletal Ni electrode was washed with deionized water to wash off the surface base, and stored in 2-propanol (IPA) for a minimum of 24 hours before the next use.

As reported in the previous studies[2], 2-propanol (IPA) was found to be a good storage solvent to maintain the activity of the Ni catalyst. IPA is also used as hydrogen provider in the Ni catalyzed catalytic hydrogen transfer reactions, the reducing ability made it an excellent organic solvent to store freshly prepared Ni electrode.

Preparation of CoP Anode Following Reported Procedure[3]

The Kanan-Nocera[3] cobalt phosphate (Co-P) water splitting anode electrode was formed in-situ during the electro-catalytic process before the injection of the organic substrate in the cathode half-cell. A flat square of stainless steel (8 mesh, 12 cm x 4 cm) was rolled up into a cylinder shape with a cross-section of 2.5 cm x 2.5 cm and immersed in the electrolyte in the anode half-cell in 30 ml of 0.1 M potassium phosphate buffer with 30 mg of $Co(NO_3)_2 \cdot 6H_2O$. Under application of electrical current, the Co-P deposited on the stainless-steel surface as a black thin coating, which enabled oxygen evolution and proton production via water oxidation.

Electrocatalytic Hydrogenation (ECH) of Diaryl Ethers[2]

The electrocatalytic hydrogenation/hydrogenolysis (ECH) of all diaryl ethers followed over previously reported procedure.[2] The two half cells were separated by a Nafion 117 membrane. The cathode half-cell was equipped with a water condenser to prevent evaporative loss of the buffer solution. The skeletal Ni cathode was placed in the cathode side with 20 mL of 0.1 M pH 8 potassium borate buffer with the addition of 8 mL 2-propanol as co-solvent. A small stir bar was also placed inside the cathode half-cell (stirring at 300 rpm). An additional 2 ml of 2-propanol was used to dissolve the weighed aryl ether substrate (0.25 mmol) in a 4-mL glass vial, in preparation for its addition to the cathode solution. The Co-P coated stainless steel anode was placed in 30 mL of 0.1 M pH 7 aqueous potassium phosphate buffer. The entire set up was placed in a water bath where the temperature was maintained at 60 °C. The divided cell containing both electrodes was equilibrated for 30 mins to obtain a constant current at 50 mA (current density 8 mA/cm^2, ~10 V). The vial with the 2 mL of 2-propanol with the aryl ether substrate was added into the cathode solution and the 50 mA current was continued for 9-12 hours at 60 °C under ambient pressure.

For the co-solvent studies, the ECH procedure is the same as above, but with adjustments on the choice of the organic co-solvent and the ratio of co-solvent to buffer.

Quantitative Analysis Preparation:
At each desired time point, 0.25 mL of cathode solution was syringed into a 1.7 mL conical vial for extraction. To extract the organics, 3 drops of diluted HCl solution (1 M) were added to the conical vial to acidify the cathode solution following by the addition of 3 drops of saturated NaCl solution, and 1 mL of dichloromethane (DCM). The conical vial was then vortexed, and the bottom DCM layer was carefully pipetted into a GC vial. The sample was analyzed through GC-MS and quantified with the calibration curves of the corresponding external standard of each compound. Every sequence of the GC-MS analyses included a set of freshly prepared external standard calibration samples. Anolyte solutions were analyzed via the same procedures at the reaction end points to test for membrane crossover of substrates and products. The crossover of starting materials was never observed, and product migration was negligible (typically $\leq$ 1%).

Volatile Product Evaporation Accounts for the Main Losses in Mass Balance

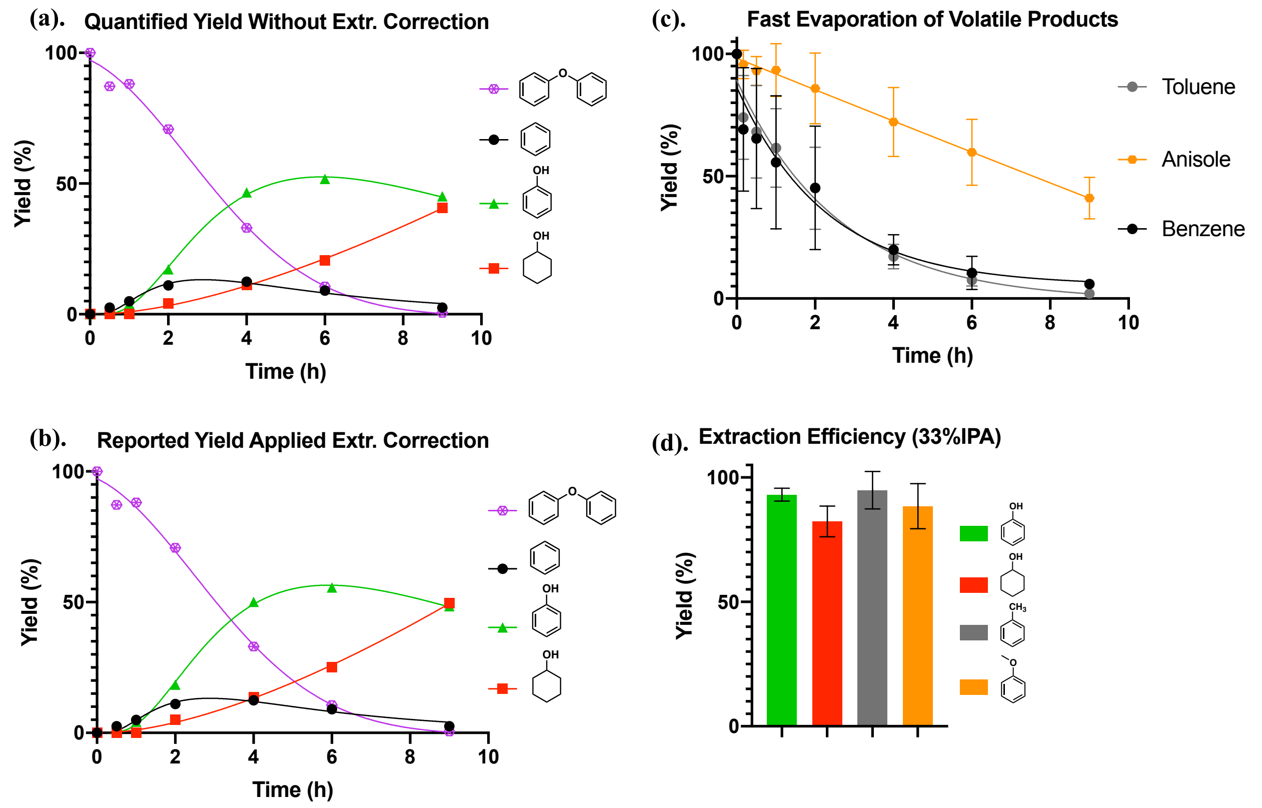

Supplementary Figure 1. (a) ECH reaction profile of diphenyl ether, showing quantified yields from GC-MS measurements. (b) ECH reaction profile of diphenyl ether, showing reported yield after extraction corrections for cyclohexanol and phenol. No yield corrections were applied for benzene or diphenyl ether. ECH performed at standard conditions, 60 °C, 50 mA and 33% v/v 2-propanol (IPA) as co-solvent. (c) Kinetic evaporation curves of benzene, toluene and anisole (non-reducible products) under standard ECH condition. (d) Extraction efficiency of analysis work up for phenol, cyclohexanol, toluene and anisole from 2:1 borate buffer/IPA electrolyte.

As indicated in *Supplementary Fig. 1c*, non-polar products with high hydrophobicity showed fast evaporation rates from the polar aqueous mixture, making yield corrections difficult. Since rate of evaporation is faster than the rate of formation of the highly volatile products, only extraction corrections for the more stable polar products were applied. Extraction correction was performed following equation [1].

$$Yield\ (reported) = \frac{Yield\ (quantified)}{Extraction\ Efficiency} \quad [1]$$

Example: Phenol yield correction at 9-hour *Supplementary Fig. 1b*

$$Yield\ (Phenol\ @\ 9h) = \frac{45\%}{0.93} = 48\%$$

H/D Isotope Exchange Experiments[2]

Following the previously reported procedure[2], a one-compartment single cell was equipped with stir bar and rubber stopper with a stainless-steel metal clip. The acetone-treated skeletal Ni catalyst (half size and same preparation as the electrode) was hung on the metal clip and placed inside the one compartment cell, positioned high enough to avoid contact with the stirrer. 10 mL of D_2O with the addition of 3 mL 2-propanol (non-deuterated) was charged inside the cell. As with the ECH studies, another 2 ml of 2-propanol was used to dissolve the weighed diaryl ether substrate (0.125 mmol) in a 4-mL glass vial, before it was added to the D_2O solution. The single cell was maintained at 60 °C, and the skeletal Ni catalyst was allowed to react with the D_2O/2-propanol mixture for 1 hour, after which the 2 mL of 2-propanol that contained the dissolved diaryl ether was added into the D_2O solution and allowed to exchange for a maximum of 12 hours at 60 °C under ambient pressure. At each desired time point, 0.25 mL of solution was sampled and analyzed by GC-MS as described above.

As previous reported[2], acetone was found to be an excellent organic solvent to discharge the stored reducing power remaining in the skeletal Ni after basic etching of the Al; thus, acetone-treated Ni catalyst was used in the labeling studies to avoid significant cleavage of the diphenyl ethers. Acetone treatment: The fresh activated skeletal Ni catalyst prepared as described above was immersed in pure acetone under nitrogen for a minimum of 24 hours.

Note, an extra step was needed to accurately determine the amount of deuterium that was incorporated in the hydroxylated analogue of diphenyl ether (DPE), since the H/D exchange on –OH functional groups happen essentially instantaneously. The exchanged samples of hydroxylated DPE were extracted as usual with 1 mL of dichloromethane, then the dichloromethane extract was washed with 1 mL of deionized non-deuterated water to make sure there was no D on the –OH functional group, prior to injection on GC-MS.

MS Quantitative Fitting of Isotope Labeling Following the Previously Established Method[2]

The total ion abundance of different mass fragments after H/D exchange as provided by GC-MS represents a mixture of D_n dimer isotopomers. The measured intensity of each ion fragment is a combination of the intensities of the same mass fragments from different D_n incorporated diaryl ether dimers. Therefore, to determine the % abundance of D_n in the exchange results, the proportion of D_n in each mixture is defined as fraction coefficient X_n (n = 0,1,2,3...n). The sum of the products of fraction coefficient X_n times peak fragment intensity I_n should be the total intensity of the corresponding peak in the total ion chromatogram. Meanwhile, the relative fragmentation pattern of different D labeled aryl ethers should not change, only the peak value will shift by one for each H that was replaced by D incorporated in the compound

(*Supplementary Fig. 2*). To avoid complications due to any isotope effects on fragmentation, only the molecular ions were analyzed in this manner.

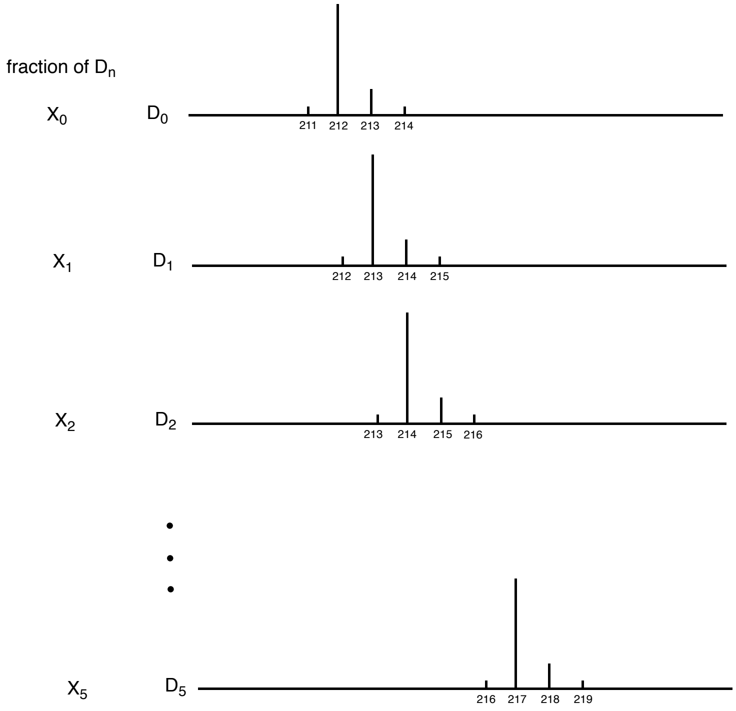

Supplementary Figure 2. As the numbers of D labels in the compound increase, the mass fragmentation pattern and intensity remain the same; only the peak's m/z value will shift. To analyze the molecular ions, fractional coefficients X_n were used as the proportions of D_n in the total ion mixture.

Excel solver was used to fit the corresponding fraction X_n of D_n in the total ion mixture. Below is an example of the solver fitting method of 4-phenoxytoluene H/D exchange at 12 hours.

As presented in **Supplementary Table 1**, column 2 is the intensity distribution of unlabeled 4-phenoxytoluene fragments (parent). Column 3 is the intensity distribution of the D labeled mixture (labeled). The fitted intensity of each ion was calculated by the below **equation 3** and tabulated in column 4-9, where fraction coefficient X_n is tabulated in column 2 (row 13-18). The values of the fractional coefficients X_n were found through excel solver as presented in **equation 5** by minimizing the sum of the square errors. The square error is calculated by **equation 4** and tabulated in column 10, where the smaller the deviation, the closer the fitted intensity to the actual labeled intensity. Based on solved X_n, the percent abundance of D_n is plotted.

$$\%Ion\ Intensity_{fitted} = X_n * \%Ion\ Intensity_{parent} \quad [3]$$

$$Square\ Error = (\sum_{0}^{5} \%Ion\ Intensity_{fitted} - \%Ion\ Intensity_{labeled})^2 \quad [4]$$

$$Solver : \left[minimize \sum_{0}^{5} Square\ error = by\ Varying\ X_n \right] \qquad [5]$$

$$\%Abundance\ (D_n) = \frac{X_n}{\sum_0^5 X_n} * 100 \qquad [6]$$

Supplementary Table 1. Para-methylated Diphenyl Ether (4-phenoxytoluene) H/D Exchange MS Fitting at 12 hrs

MS Frag. (m/z)	MS Intensity (parent)	MS Intensity (labeled)	Fitted Int. X_0*Parent	Fitted Int. X_1*Parent	Fitted Int. X_2*Parent	Fitted Int. X_3*Parent	Fitted Int. X_4*Parent	Fit. Int. X_5*P	Square Error
180	0.02	0	0.00340395						1.1587E-05
181	1.52	0.37	0.25869992	0.00769147					0.01073474
182	0.72	1.12	0.12254207	0.58455198	0.01638631				0.15722783
183	15.28	4.51	2.600615	0.27689304	1.24535969	0.01558025			0.1380509
184	100	24.61	17.0197317	5.87628564	0.58990722	1.18409914	0.00131766		0.00376276
185	15.34	54.25	2.61082684	38.4573668	12.5191421	0.56088906	0.10014198	0.0001927 3	2.075E-06
186	1.23	100	0.2093427	5.89936006	81.9315582	11.9033124	0.04743567	0.0146475 6	3.1997E-05
187	0.07	91.97	0.01191381	0.47302561	12.568301	77.901259	1.00669043	0.0069383 2	3.5035E-06
188	0	19.72	0	0.02692016	1.00775817	11.9500531	6.58828817	0.1472465 5	7.0851E-08
189	0	2.99	0	0	0.05735209	0.95818549	1.01064341	0.9636554 5	2.6755E-08
Deuterium number (D_n)	Fraction Coefficient X_n	% Abundance							∑ Square Errors
D0	0.17019732	7.63696119							0.30982549 7
D1	0.38457367	17.2562895							
D2	0.81931558	36.7636894							
D3	0.77901259	34.955245							
D4	0.06588288	2.95624525							
D5	0.00963655	0.43240395							

Analogous analyses applied to the fragments, together with NMR evidence, confirm the assigned locations of the D labels.

Fitting Results: % D_n Incorporation During H/D Exchange of Diphenyl Ether

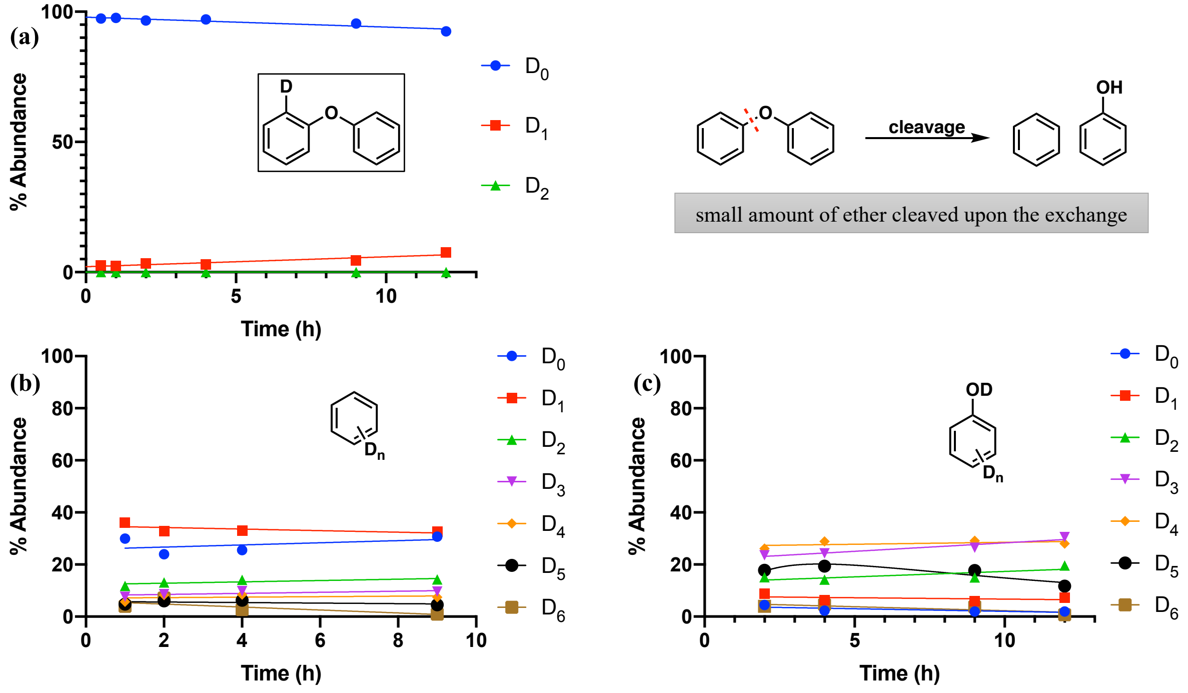

Supplementary Figure 3. Quantified % abundance of D_n in (a) diphenyl ether and cleavage products (b) benzene and (c) phenol, as determined by GC-MS for (MS Spectra shown below in *Supplementary Fig. 4*). The exchange locations in DPE were determined based on NMR evidence (*Supplementary Fig. 6* below). Experiment was run without current flowing, so only a small amount of diphenyl ether underwent cleavage during the exchange experiment.

The formation of di-deuterated benzene points to the proposed cleavage Route I via formation of a benzyne intermediate. The isotope labeling found in the benzene product showed ratios of di-, mono- and undeuterated products that were essentially fixed over the course of the reaction. Remembering that the skeletal nickel electrodes employed in these studies have been activated via etching in protic media, they are expected to retain substantial H on the surface, some of which exchange with deuterium in the D_2O reaction medium. Presumably, the H atom from the ortho C-H activated by oxidative insertion also remains nearby, so that when two surface hydrogens are donated to reduce the benzyne and release it as benzene, there is a high probability that one of them will still be protium. However, the dideuterated products appear as a substantial component of the mixture, in fixed proportion to the monolabeled material (especially in the methoxy and methyl substituted cases below). This clearly indicates the involvement of that ortho C-H site.

Labeling in the phenol is less informative; phenol itself undergoes H/D exchange that is fast on the Ni surface, especially at the ortho positions, and it is also rapidly reduced to cyclohexanol.

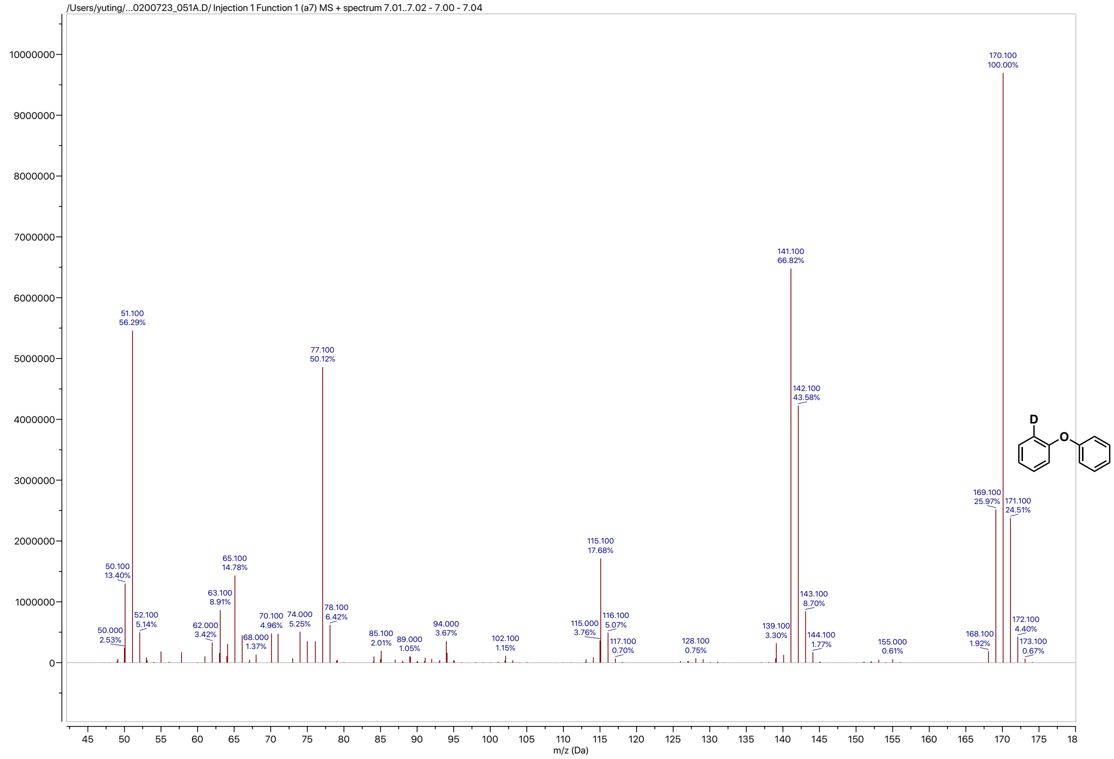

Supplementary Figure 4. Mass spectrum of **labeled** diphenyl ether after 12 hours H/D exchange at 60 °C. Compared to the unlabeled parent *Supplementary Fig. 5*), no significant increase of m/z 171, which suggested the low D incorporation of diphenyl ether. The location of the exchange was further confirmed by NMR.

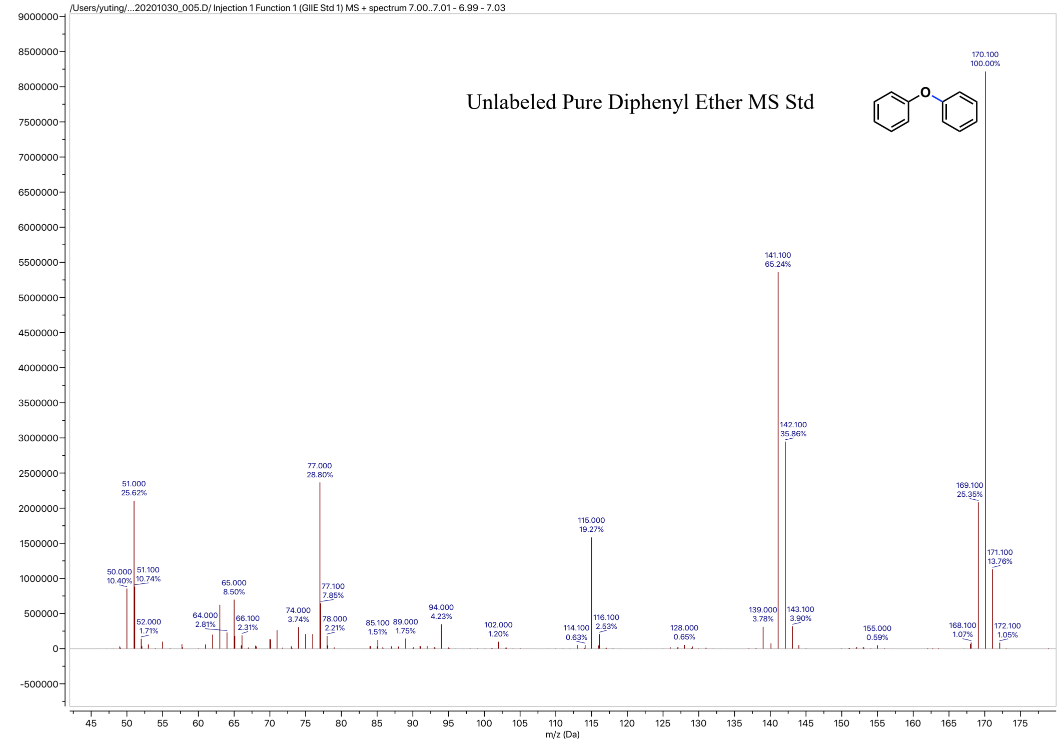

Supplementary Figure 5. Mass spectrum of **unlabeled** diphenyl ether.

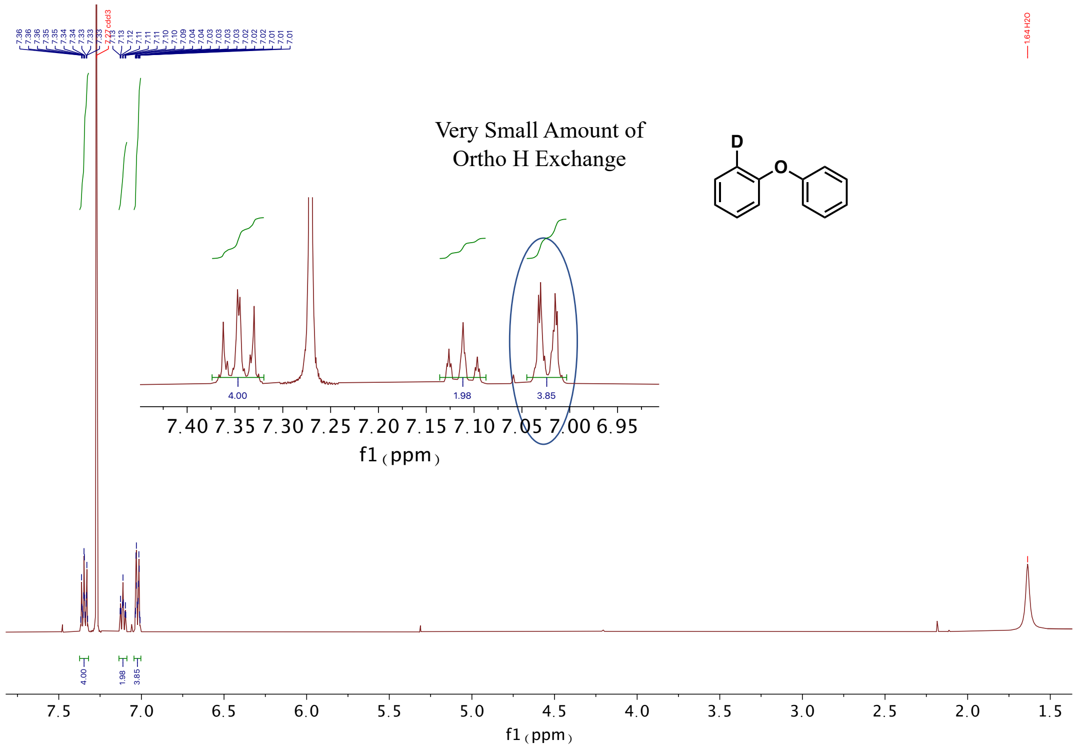

Supplementary Figure 6. NMR spectrum of **labeled** diphenyl ether after H/D exchange for 12 h. As indicated in the figure, only a small amount of the ortho hydrogens were exchanged, which also matched up with the quantified exchange abundance from the mass spectra result.

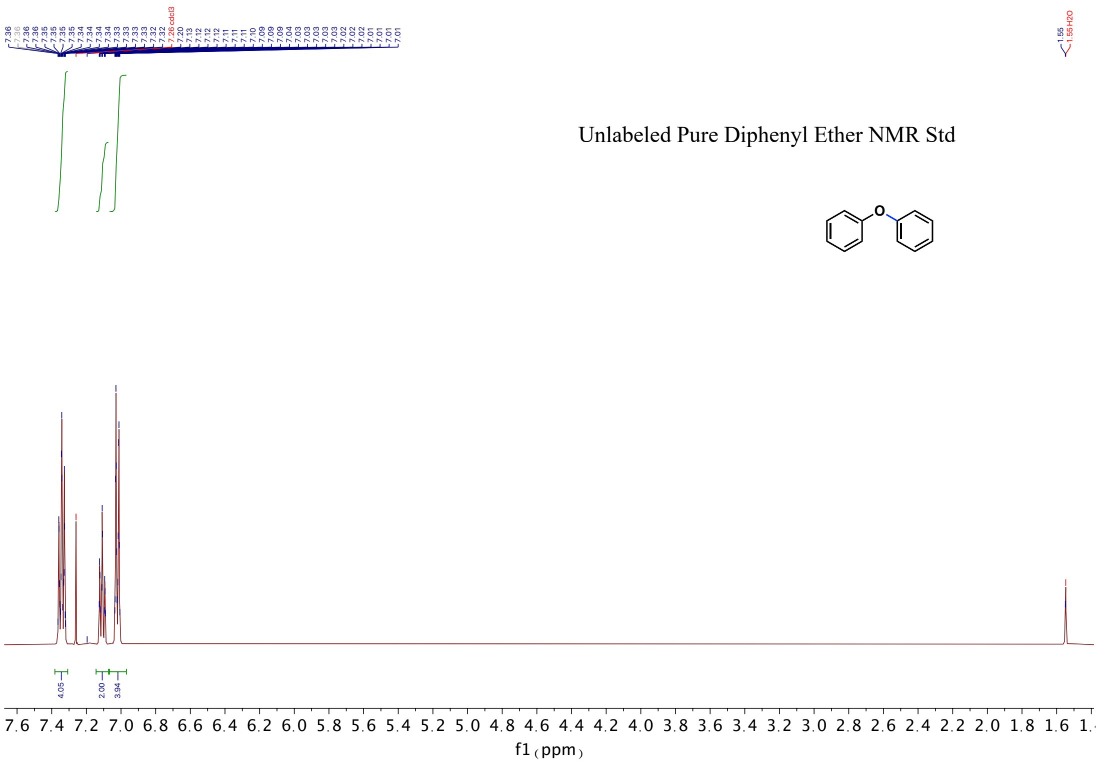

Supplementary Figure 7. NMR spectrum of **unlabeled** diphenyl ether as reference for the labeled spectra.

Fitting Results: % D_n Incorporation During H/D Exchange of Diphenyl Ether with <u>Applied Current (50 mA)</u>

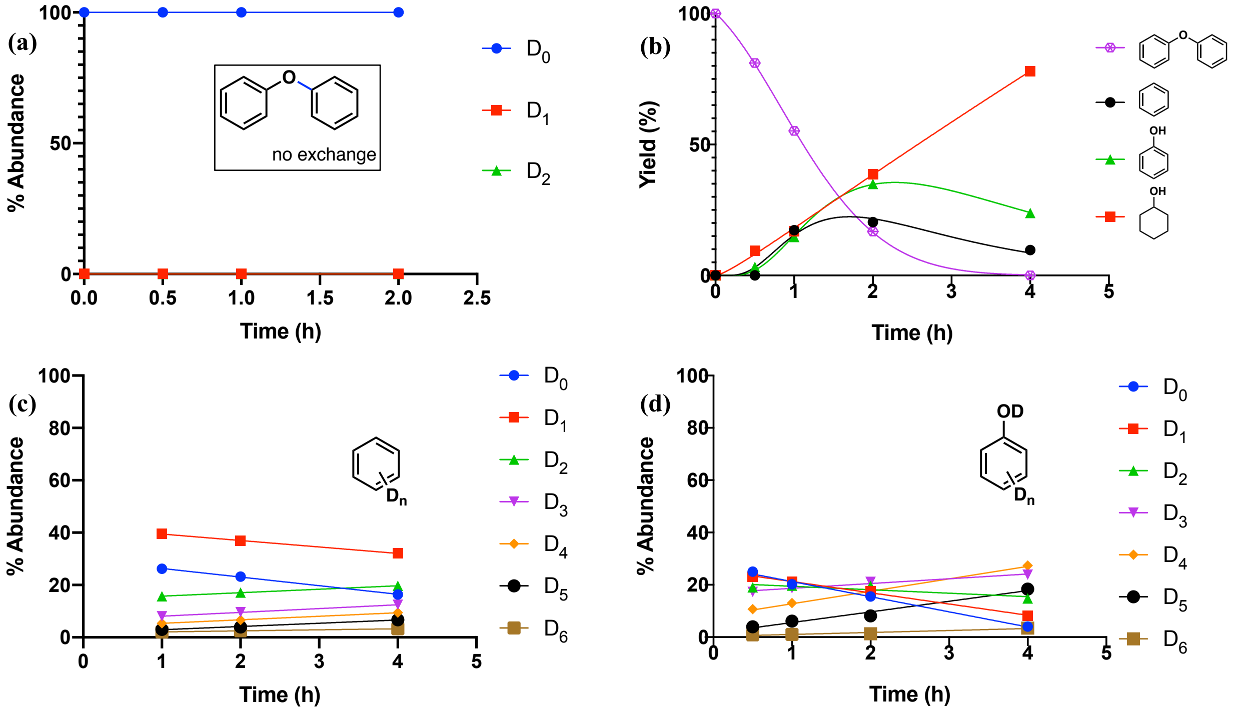

Supplementary Figure 8. (a) Quantified % abundance of D_n in diphenyl ether determined by GC-MS. The H/D exchange experiment with applied current (50 mA) was conducted following the ECH general procedure as described above. As shown in Figure (a), no deuterium was detected in the remaining diphenyl ether dimer. But as indicated in (b), diphenyl ether was constantly undergoing cleavage into the products, which did contain substantial amounts of D. This finding suggests that the C-O cleavage proceeded immediately once the ortho C-H site was activated. Since the exchange experiment was performed at a smaller scale (1/2 size of the normal ECH reaction), a smaller ECH cell set up was used, which somewhat affects the overall cleavage rate, since the smaller cell has a longer bridge between the two electrodes. (c) Quantified % abundance of D_n in the benzene product. (d) Quantified % abundance of D_n in phenol. As expected, substantial amounts of D were incorporated in the cleavage products during the fast cleavage under applied current. Free benzene does not undergo H/D exchange itself at significant rates under these reaction conditions; the upward slope in D_2 and more highly labeled products presumably reflects the increasing proportion of D formed on the cathode surface as residual H atoms are consumed. Isotope effects are unknown, though they would be expected to favor H vs D transfer to the departing benzene.

Full ECH Time Courses for Diphenyl Ether, 4-Phenoxytoluene, 4-Phenoxyanisole and 4-Phenoxyphenol

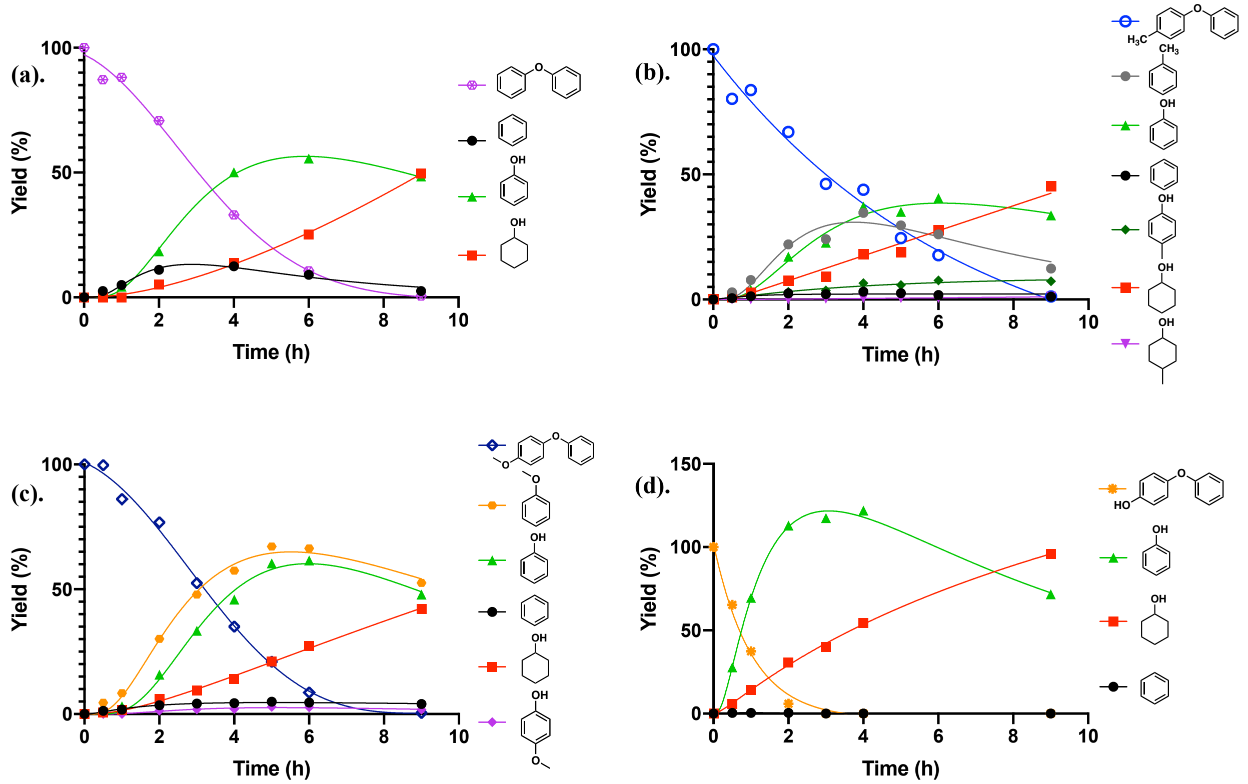

Supplementary Figure 9. Full ECH measurements of diphenyl ether (a), para methylated diphenyl ether (b), para methoxylated diphenyl ether (c) and also para hydroxylated diphenyl ether (d), under standard ECH conditions (50 mA, 60°C, 33%IPA). The yields of cyclohexanol and phenol were corrected for extraction losses (*Supplementary Fig. 1d*). The yield of 4-methoxyphenol was corrected for extraction loss (*Supplementary Fig. 58*). The yield of p-Cresol was corrected using the extraction efficiency of phenol (*Supplementary Fig. 1d*). No yield corrections were applied on the starting diphenyl ethers and the alkyl benzenes. Note that yield values exceeding 100% in (d) reflect the fact cleavage yields two phenol molecules per phenoxyphenol cleaved. No traces of benzene and dihydroxylated products were detected in these experiments.

Fitting Results: % D_n Incorporation During H/D Exchange of Para-Methoxylated Diphenyl Ether

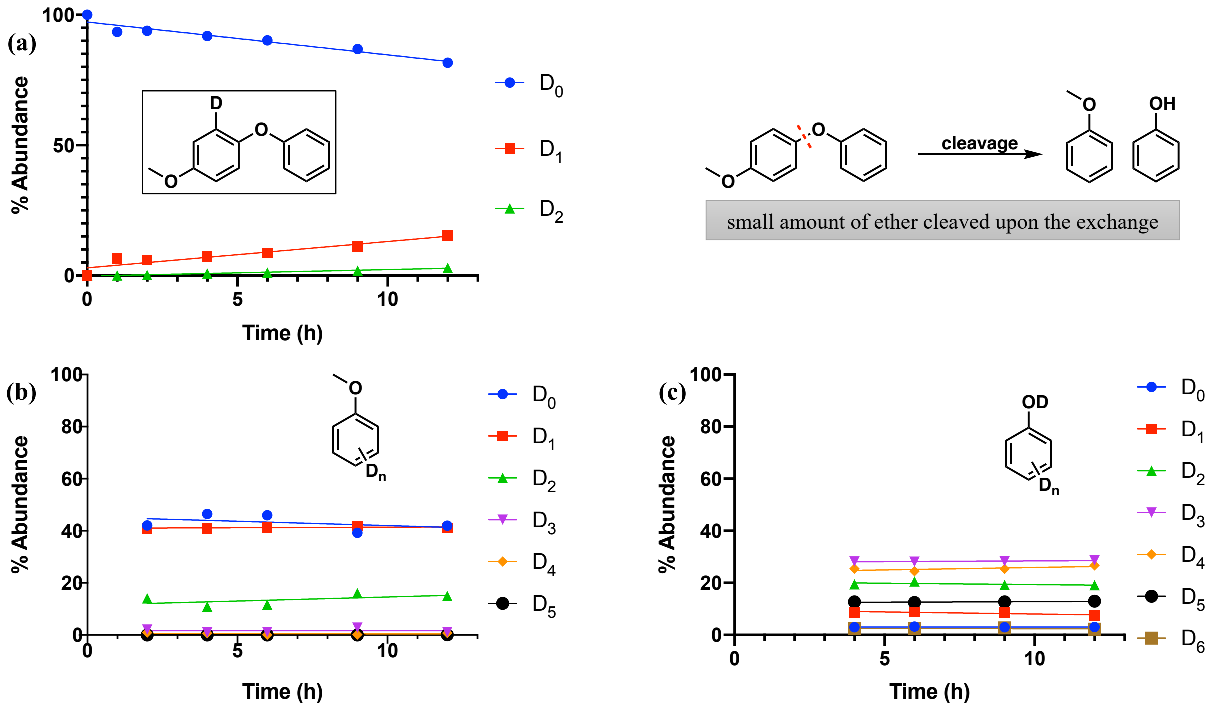

Supplementary Figure 10. (a) Quantified % abundance of D_n of 4-phenoxyanisole determined by GC-MS (MS Spectra shown below *Supplementary Fig. 11*). The exchange location was determined by NMR (*Supplementary Fig. 13* below). Though only a small amount of the ether substrate underwent cleavage during the exchange experiment, it was also possible to determine the %D of the cleaved products: (b) Quantified % abundance of D_n in anisole. (c) Quantified % abundance of D_n in phenol.

As in the plain DPE case, the existence of di-deuterated anisole in this case supports the cleavage proposed in route I via the 4-methoxybenzyne intermediate. The isotope labeling found in anisole also showed a fixed ratio of di-, mono- and undeuterated products in fixed proportions, with the dideuterated products representing a substantial component of the mixture. Again, this clearly indicates the involvement of that ortho C-H site.

As noted in *Supplementary Fig. S3* (page S9), the phenol product undergoes fast exchange on the Ni surface, and is also rapidly reduced to cyclohexanol.

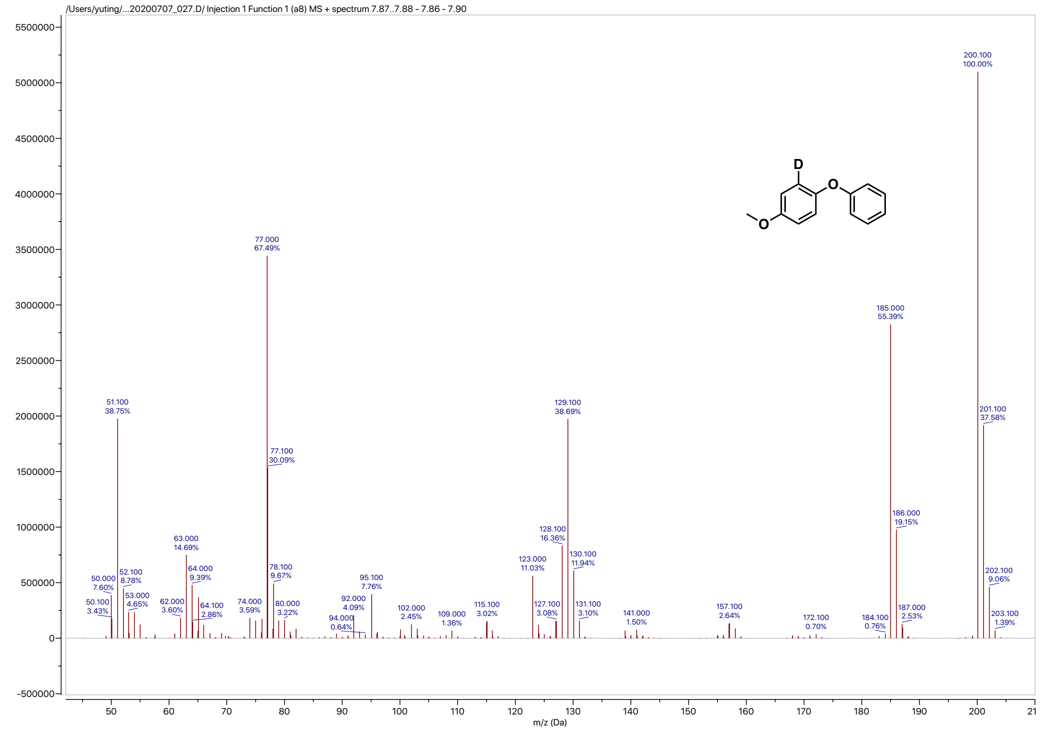

Supplementary Figure 11. Mass spectrum of **labeled** 4-phenoxyanisole after 12 hours H/D exchange at 60 °C. Compared to the unlabeled parent (*Supplementary Fig. 11*), small increases in m/z 201 and 202 peaks are visible, which suggested slow D incorporation. The location of the exchange was further confirmed by NMR.

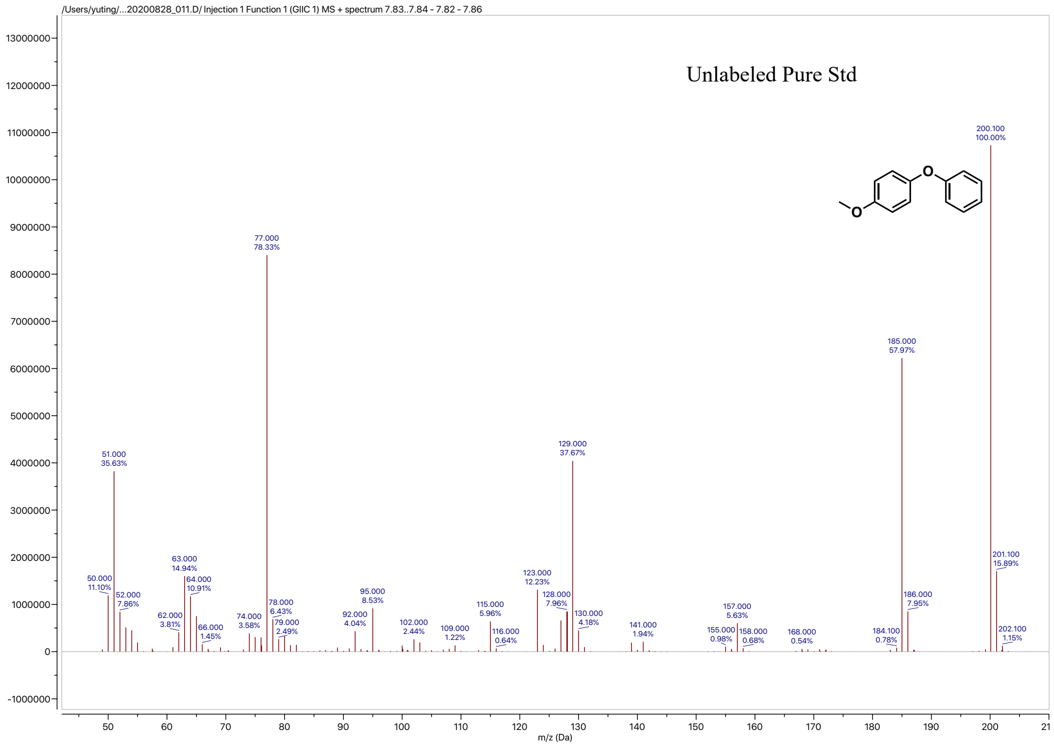

Supplementary Figure 12. Mass spectrum of **unlabeled** 4-phenoxyanisole.

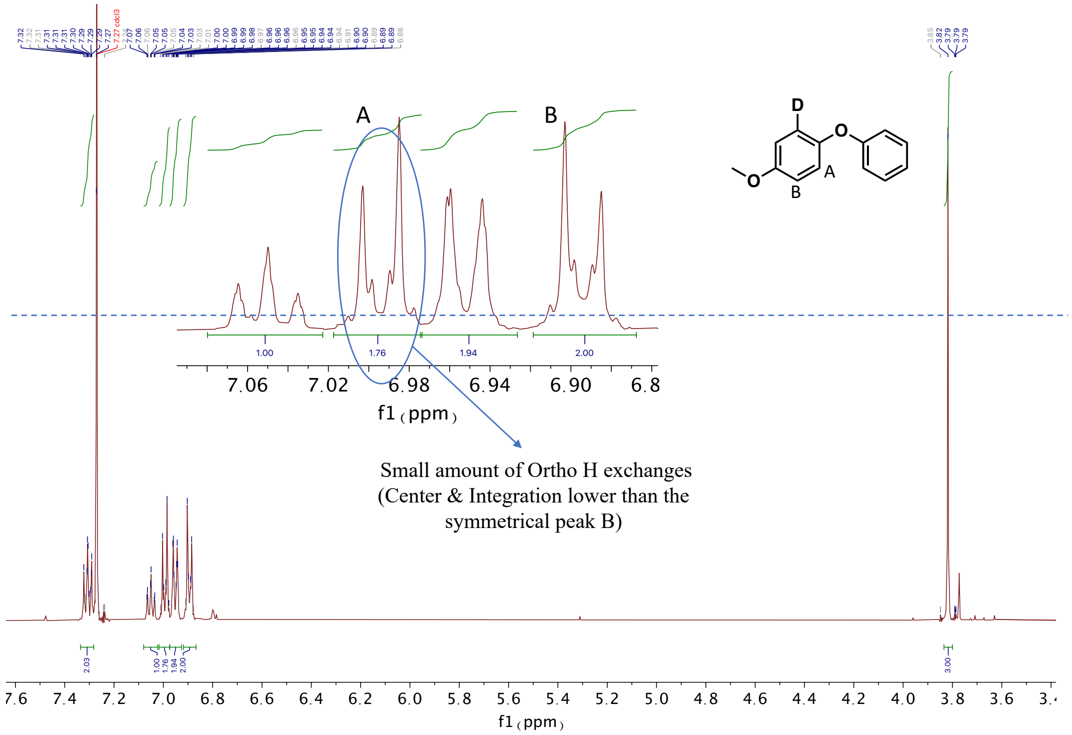

Supplementary Figure 13. NMR spectrum of **labeled** 4-phenoxyanisole after 12 hours exchange. As highlighted, the integrate of peak A had a mild decrease compared to its symmetrical partner peak B. Despite no significant shape difference between peaks A and B, as indicated by the dotted line, the center of peak A is lower than that of B.

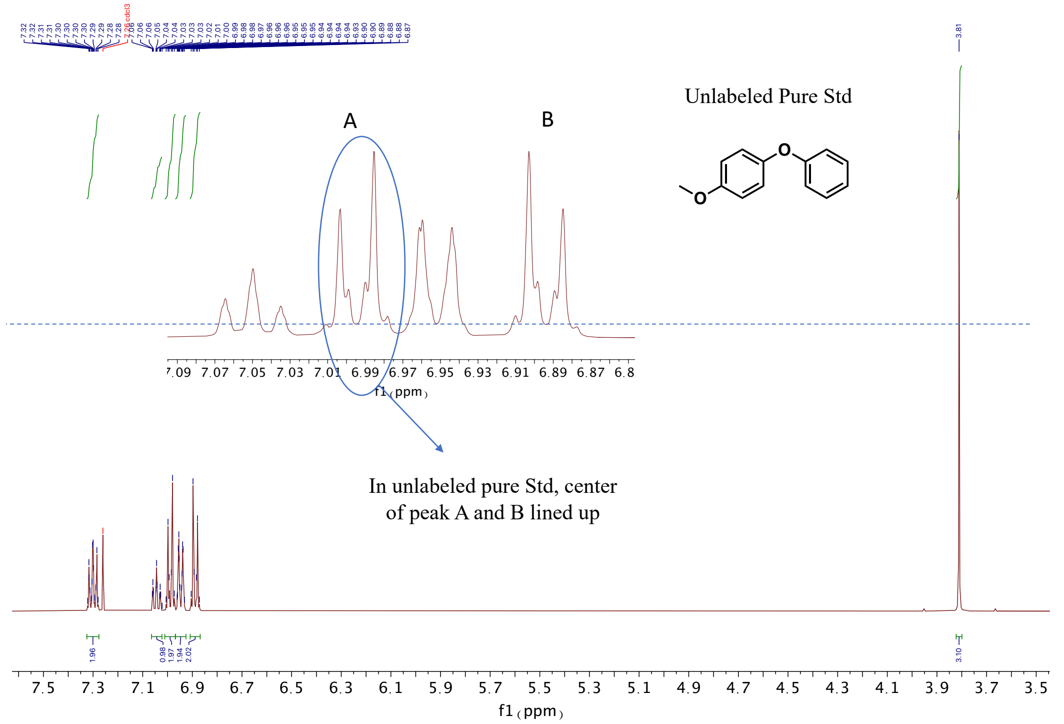

Supplementary Figure 14. NMR spectrum of **unlabeled** 4-phenoxyanisole. Note that in the unlabeled pure compound, the centers of peaks A and B do line up.

Fitted Kinetic Measurement of D_n in H/D Exchange of Para-Methylated Diphenyl Ether

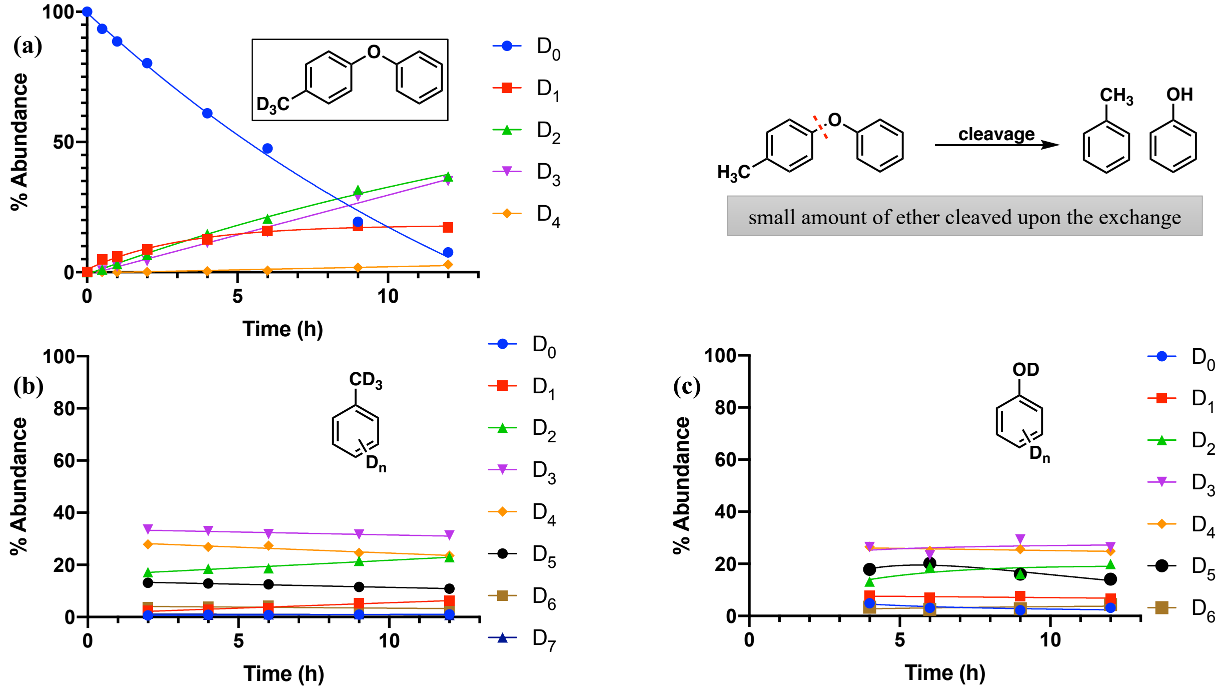

Supplementary Figure 15. (a) Quantified % abundance of D_n in 4-phenoxytoluene determined by GC-MS (MS Spectra shown as *Supplementary Fig. 16* below). The exchange location is determined by NMR (*Supplementary Fig. 18* below). Though only a small amount of the ether substrate underwent cleavage during the exchange experiment, it was also possible to determine the %D in the cleaved products. (b) Quantified % abundance of D_n in toluene. (c) Quantified % abundance of D_n in phenol.

As in the previously analyzed cases (*Supplementary Fig. 3*, page S9), the existence of penta-deuterated toluene in this case further supported the proposed cleavage in route I via a 4-methylbenzyne intermediate. The isotope labeling found in toluene also showed essentially fixed ratios among the tri-, tetra- and penta-deuterated products, with the penta-deuterated product appearing as a substantial component of the mixture, in almost fixed proportion to the tri- and tetra-deuterated material. Again, this clearly indicates the involvement of the ortho C-H site in the formation of the toluene product.

As noted earlier, the phenol product undergoes fast H/D exchange on Ni surface and is also rapidly reduced to cyclohexanol.

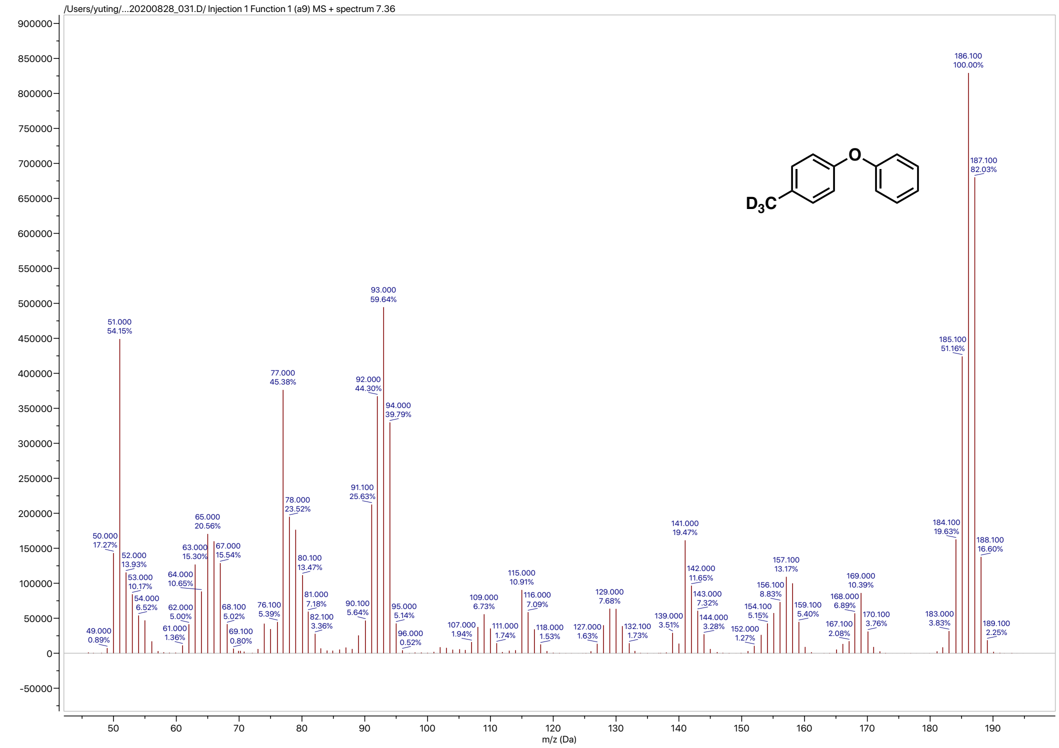

Supplementary Figure 16. Mass spectrum of **labeled** 4-phenoxytoluene after 12 hours H/D exchange. The location of the exchange was further confirmed by NMR.

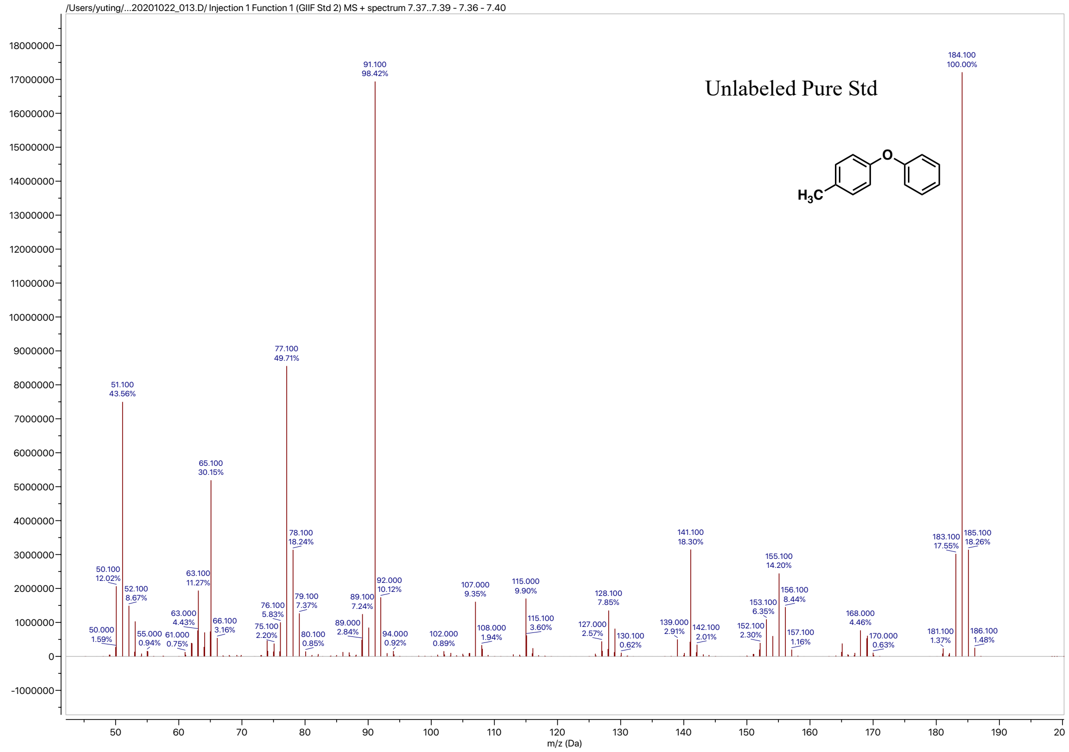

Supplementary Figure 17. Mass spectrum of **unlabeled** 4-phenoxytoluene.

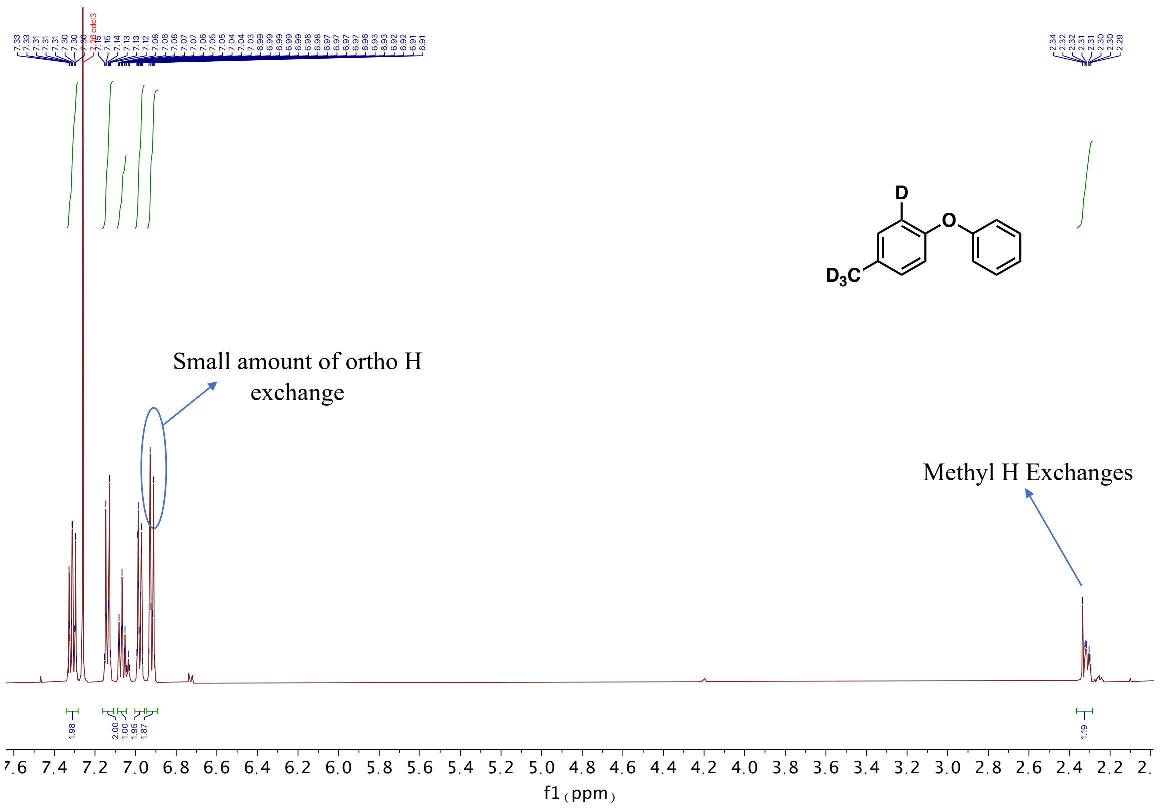

Supplementary Figure 18. NMR spectrum of **labeled** 4-phenoxytoluene after 12 hours exchange.

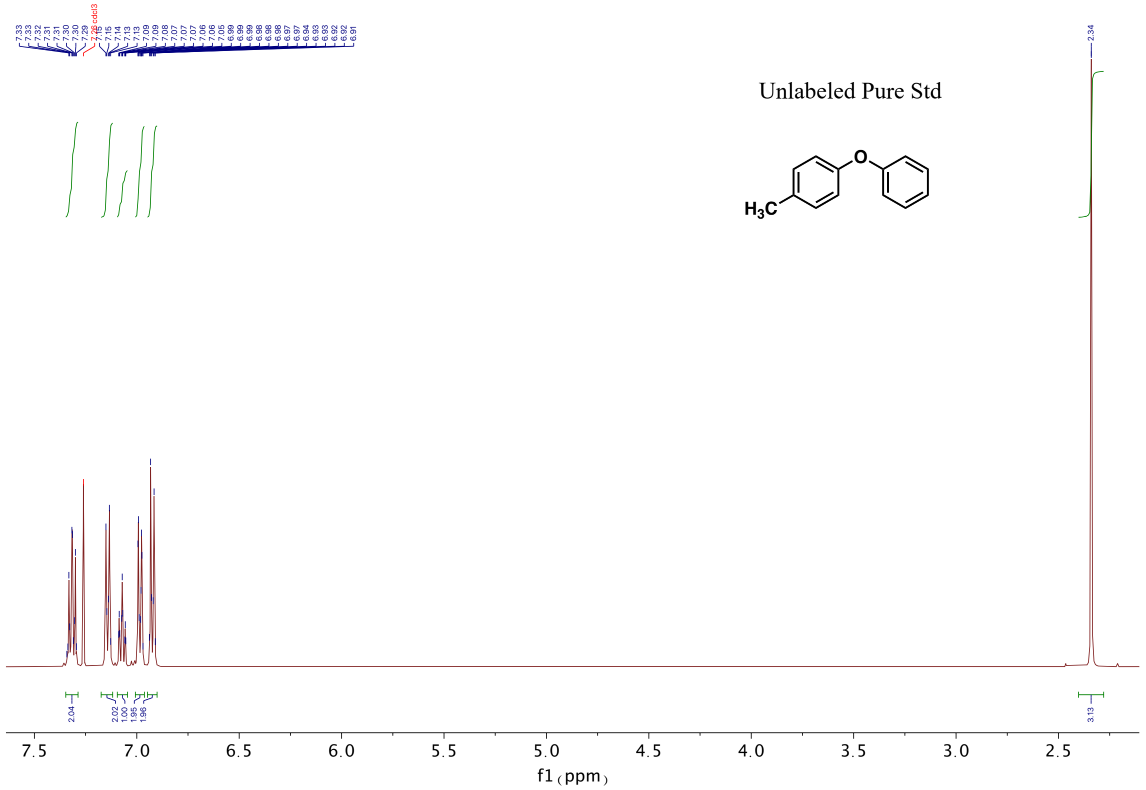

Supplementary Figure 19. NMR spectrum of **unlabeled** 4-phenoxytoluene.

Fitted Kinetic Measurement of D_n in H/D Exchange of Para-Hydroxylated Diphenyl Ether

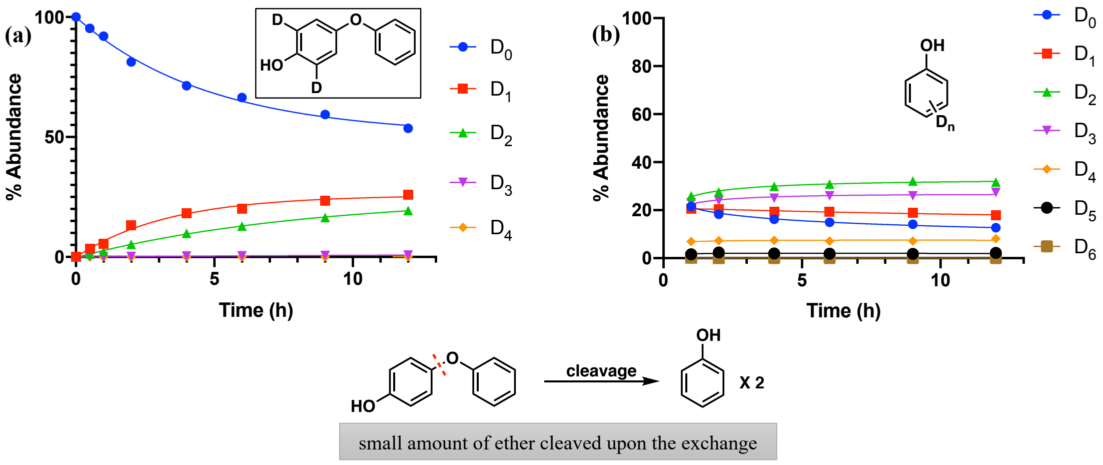

Supplementary Figure 20. (a) Quantified % abundance of D_n of 4-phenoxyphenol determined by GC-MS (MS Spectra shown as *Supplementary Fig. 21* below). (b) Quantified % abundance of D_n of phenol, which undergoes fast H/D exchange on the Ni surface. The D incorporated on the hydroxyl site is exchangeable, so the MS samples of 4-phenoxyphenol had an extra wash using non-deuterated water. The exchange locations in the 4-phenoxyphenol starting material here were determined by NMR (*Supplementary Fig. 23* below).

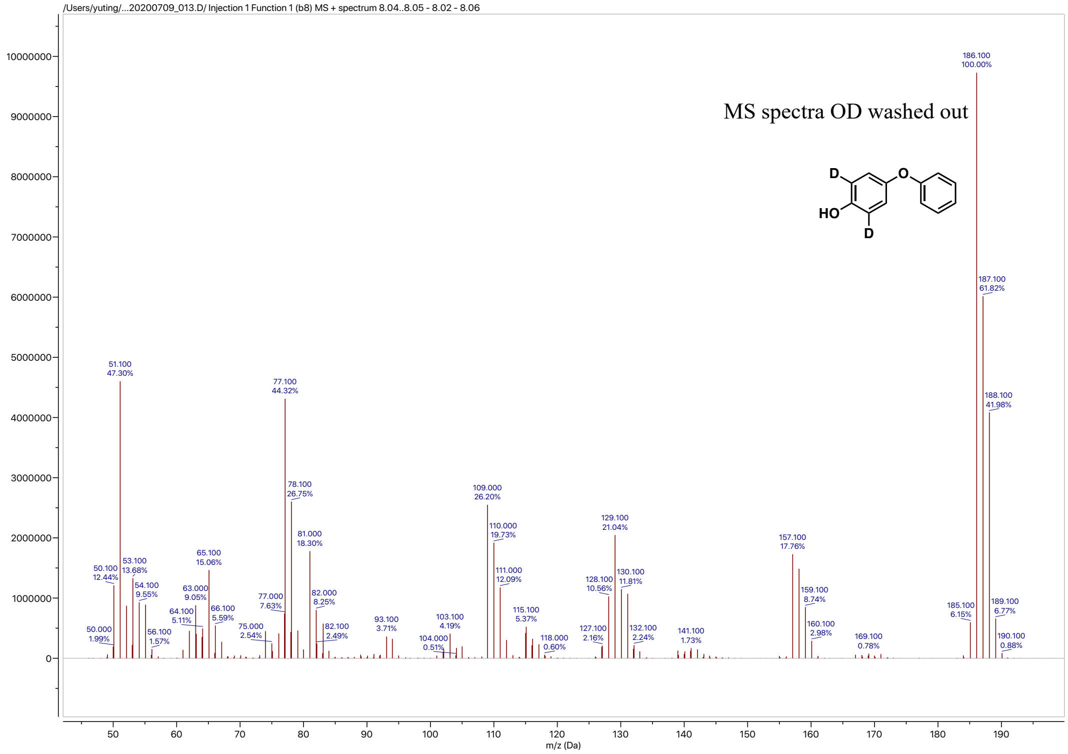

Supplementary Figure 21. Mass spectrum of **labeled** 4-phenoxyphenol after 12 hours H/D exchange. The deuterium in the hydroxyl group was washed off by non-deuterated DI water to properly count the D exchanged in the aromatic protons. The location of the exchange was further confirmed by NMR.

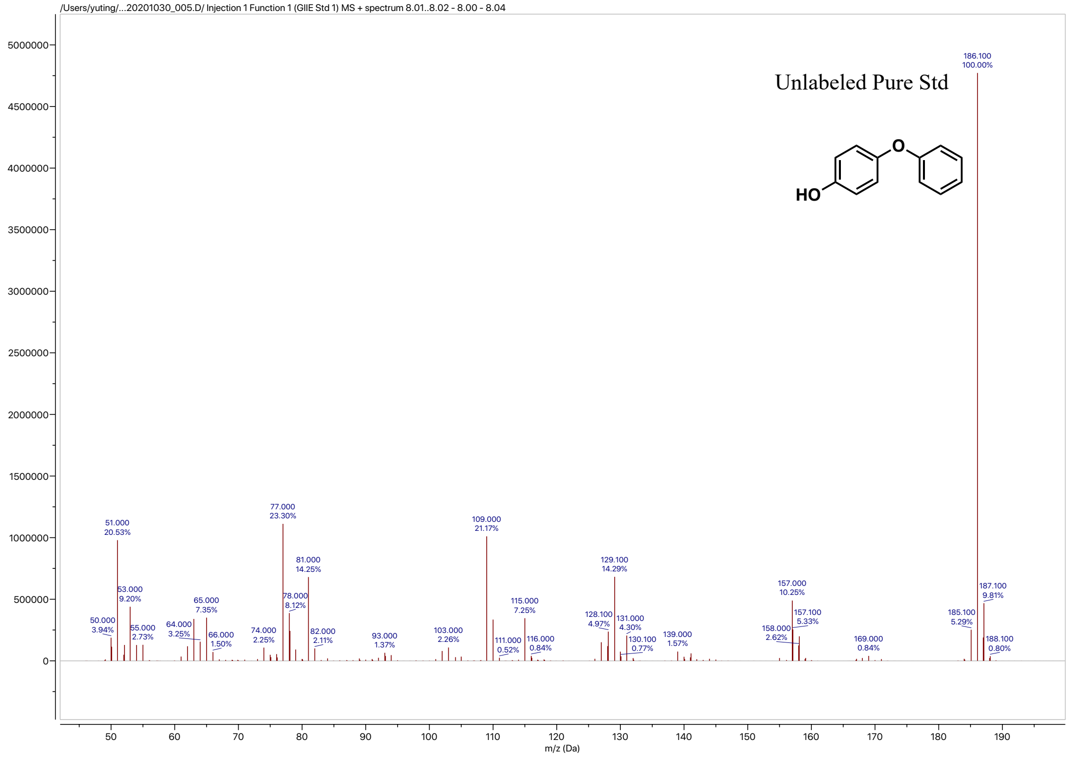

Supplementary Figure 22. Mass spectrum of **unlabeled** 4-phenoxyphenol.

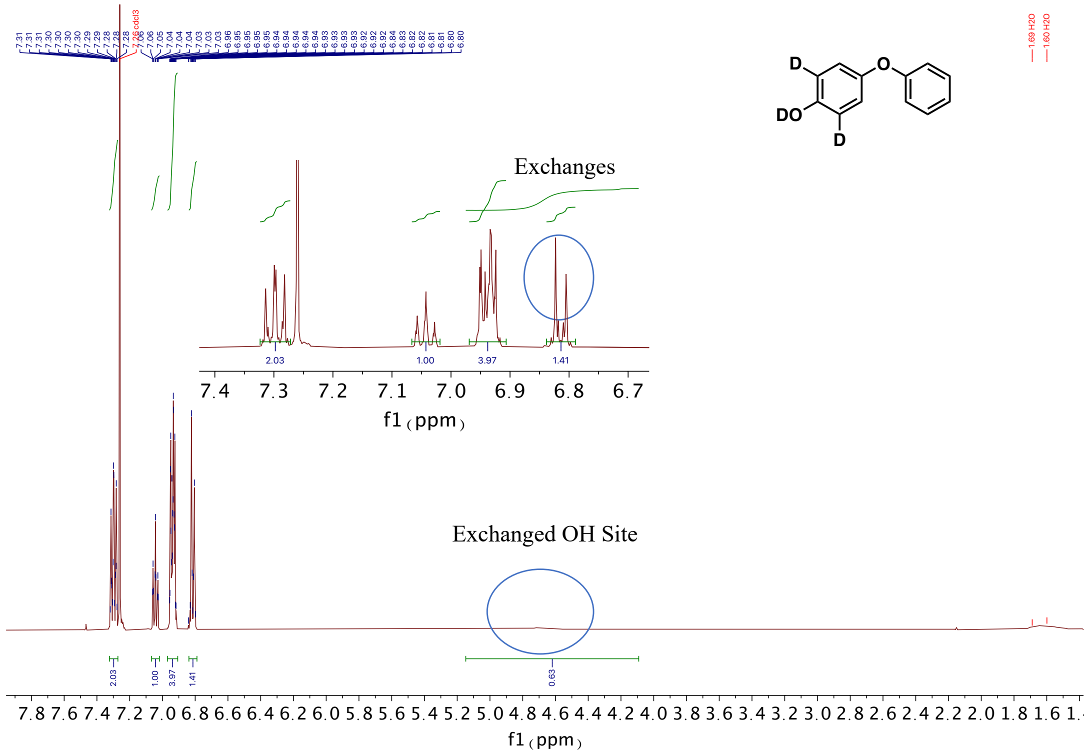

Supplementary Figure 23. NMR spectrum of **labeled** 4-phenoxyphenol after 12 hours exchange. In ^{1}H NMR analysis, OD peak was separated from the aromatic protons, no DI water wash was needed.

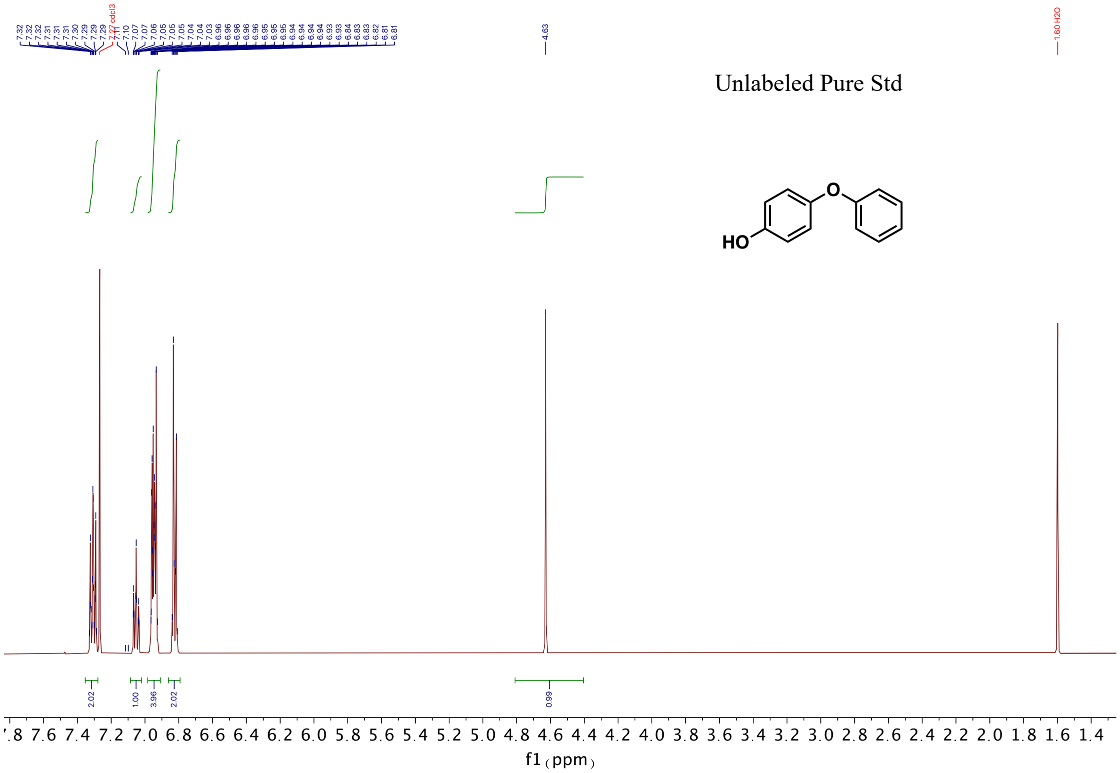

Supplementary Figure 24. NMR spectrum of **unlabeled** 4-phenoxyphenol.

Full ECH Time Courses for para, meta and ortho Methoxylated Diphenyl Ethers

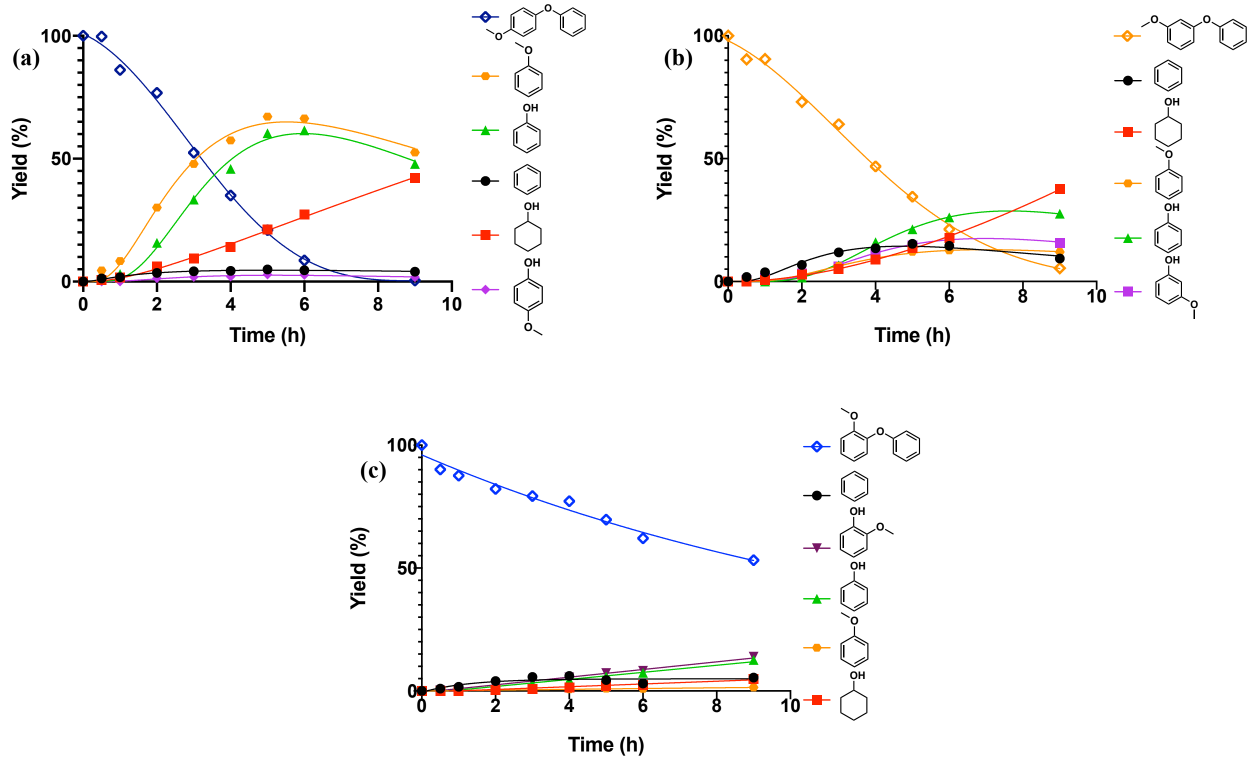

Supplementary Figure 25. Full ECH measurements of (a) para-methoxylated diphenyl ether, (b) meta-methoxylated diphenyl ether and (c) ortho-methoxylated diphenyl ether at standard ECH condition (50 mA, 60 °C and 33% IPA). The yields of cyclohexanol, anisole and phenol were corrected for extraction losses (*Supplementary Fig. 1d*), as were the yields of 4-methoxyphenol and 2-methoxyphenol (*Supplementary Fig. 58*). The yield of 3-methoxyphenol was corrected using the extraction efficiency of 4-methoxyphenol (*Supplementary Fig. 58*). No yield corrections were applied on the starting diphenyl ethers and the benzene.

Full ECH Time Courses for para, meta and ortho Methylated Diphenyl Ethers

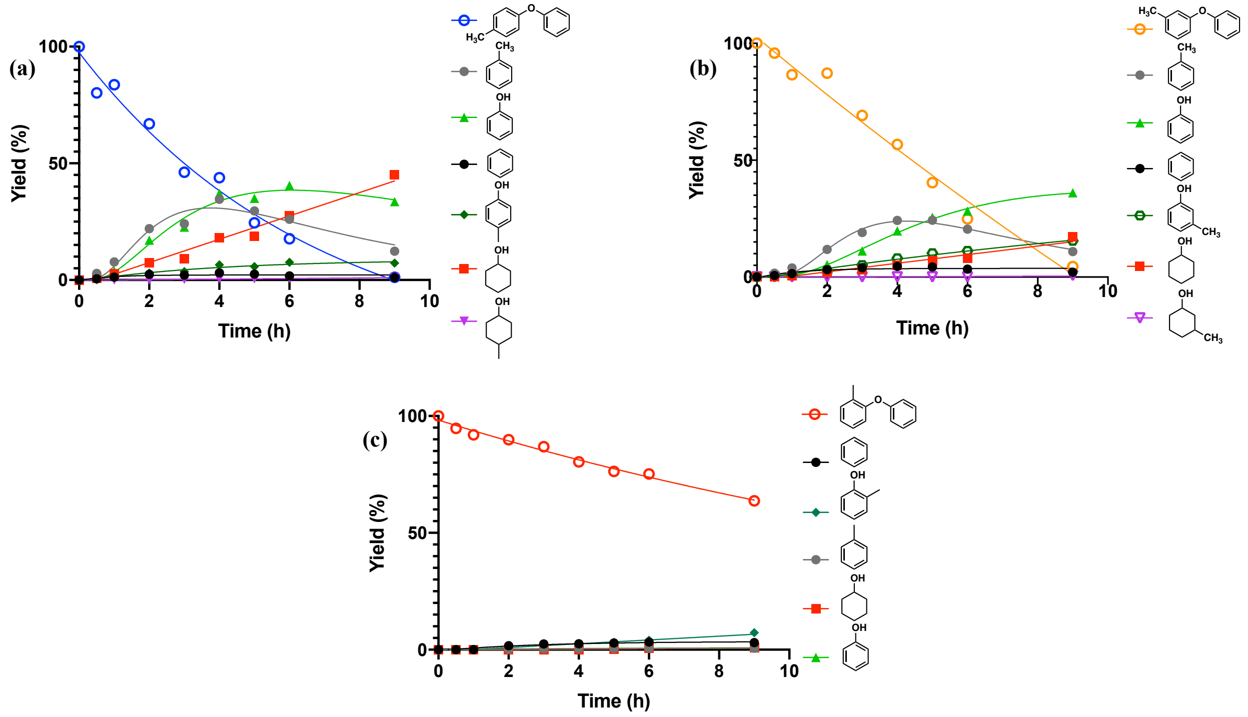

Supplementary Figure 26. Full ECH measurements of (a) para-methylated diphenyl ether, (b) meta-methylated diphenyl ether and (c) ortho-methylated diphenyl ether at standard ECH condition (50 mA, 60 °C and 33% IPA). The yields of cyclohexanol and phenol were corrected for extraction losses (*Supplementary Fig. 1d*). The yields of cresols were corrected using the extraction efficiency of phenol (*Supplementary Fig. 1d*). No yield corrections were applied on the starting diphenyl ethers, methyl cyclohexanols or alkyl benzenes.

Full ECH Time Courses for para, meta and ortho Hydroxylated Diphenyl Ethers

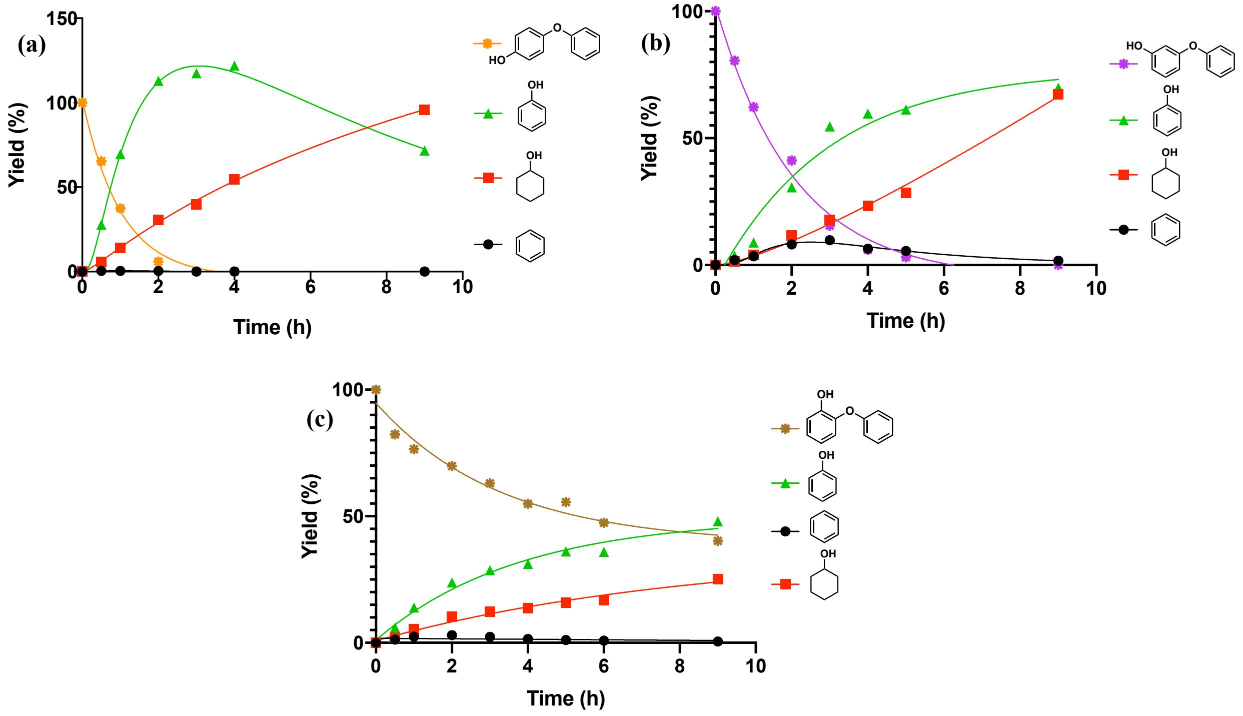

Supplementary Figure 27. Full ECH measurements of (a) para-hydroxylated diphenyl ether, (b) meta-hydroxylated diphenyl ether and (c) ortho-hydroxylated diphenyl ether at standard ECH condition (50 mA, 60 °C and 33%IPA). The yields of cyclohexanol and phenol were corrected for extraction losses (*Supplementary Fig. 1d*). No yield corrections were applied on the starting diphenyl ethers and the benzene. Note that for meta-hydroxylated DPE, small amounts of 1,3-dihydroxybenzene were formed (also implied by the benzene observed) as the reaction progressed, but due to its high hydrophilicity, it was difficult to extract that diol product into dichloromethane for GC-MS analysis.

Cleavage Regioselectivity of Asymmetric Diaryl Ethers

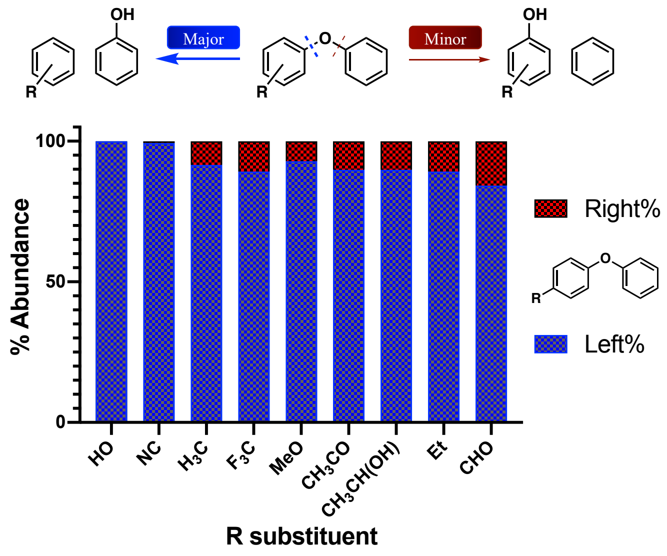

Supplementary Figure 28. Cleavage regioselectivity of various para-functionalized diphenyl ethers.

Example of Regioselectivity Calculation (4-phenoxytoluene):

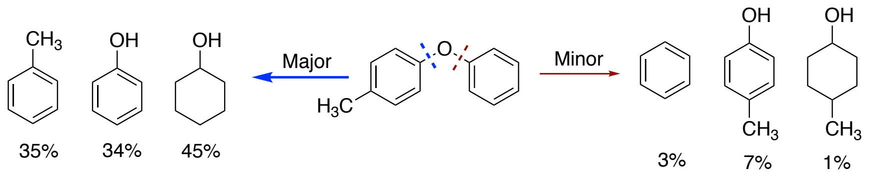

In both major and minor cleavage, the highly non-polar and volatile products benzene and toluene were gradually lost as the reactions progressed (*Supplementary Fig. 1c*). Thus, the regioselectivity % abundance was calculated based on the more stable polar products (cyclohexanol, phenol, p-cresol and 4-methylcyclohexanol).

$$Right\ Cleavage\ \% = \frac{\sum right\ polar\ products}{\sum total\ polar\ products} = \frac{7+1}{34+45+7+1} * 100 = 9.2\%$$

$$Left\ Cleavage\ \% = \frac{\sum left\ polar\ products}{\sum total\ polar\ products} = \frac{34+45}{34+45+7+1} * 100 = 90.8\%$$

Regioselectivity of all listed substituted diphenyl ethers were calculated using this general procedure.

Cleavage Regioselectivity Is Not Significantly Influenced by Applied Current/Potential or Temperature

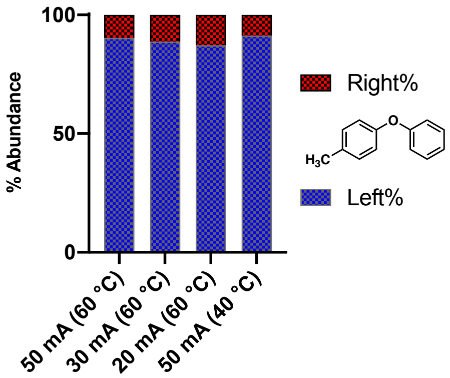

Supplementary Figure 29. Regioselectivity distribution of para methylated diphenyl ether cleavage under different currents and temperatures.

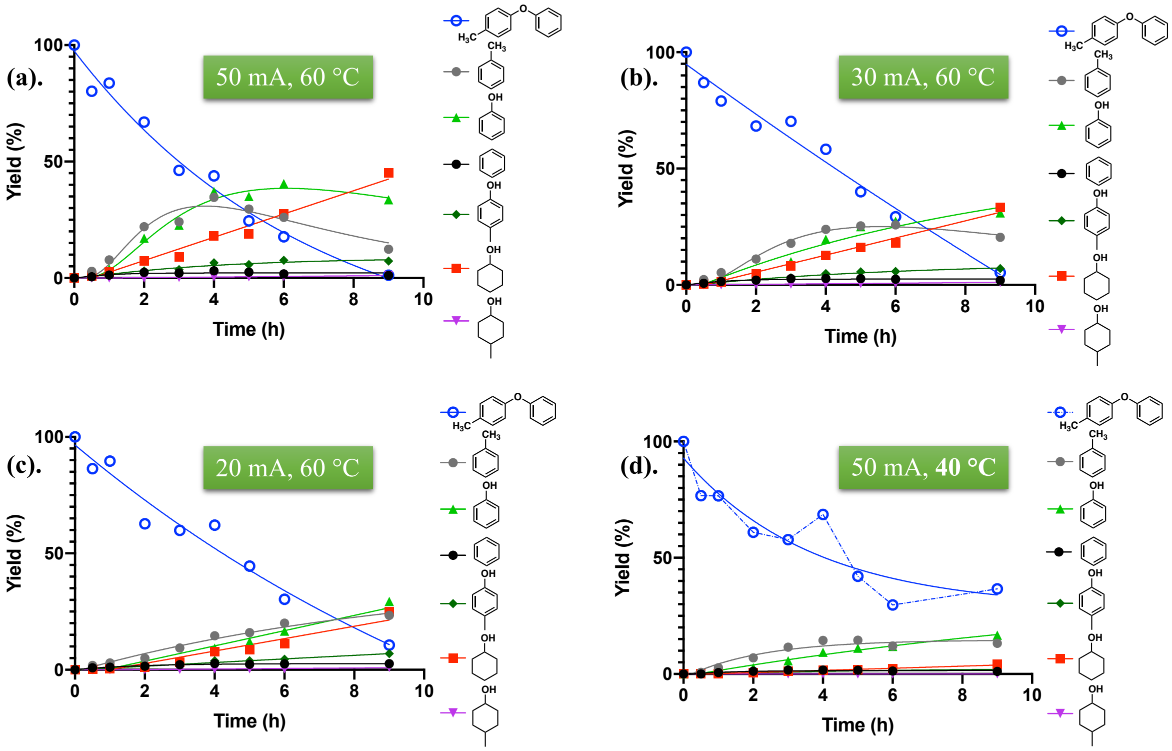

Supplementary Figure 30. Full ECH measurements of para-methylated diphenyl ether with different applied currents and temperatures (a) 50 mA (~10 V), 60 °C and 33% IPA (b) 30 mA (~7 V), 60 °C and 33% IPA (c) 20 mA (~5.7 V), 60 °C and 33% IPA (d) 50 mA, 40 °C (decreased DPE solubility at this lower temperature) and 33% IPA. The yields of cyclohexanol and phenol were corrected for extraction losses (*Supplementary Fig. 1d*). The yields of cresols were corrected using the extraction efficiency of phenol (*Supplementary Fig. 1d*). No yield corrections were applied on the starting diphenyl ethers, methyl cyclohexanols or alkyl benzenes.

Both the cleavage products and regioselectivity were not impacted by the lower current and applied potential; only the C-O cleavage rate was significantly slower under the decreased current and temperature conditions, presumably due to the lower rate of hydrogen formation on the Ni metal surface.

Hydroxylated DPE: The Outlier, Regioselectivity Was Not Altered by OH Position

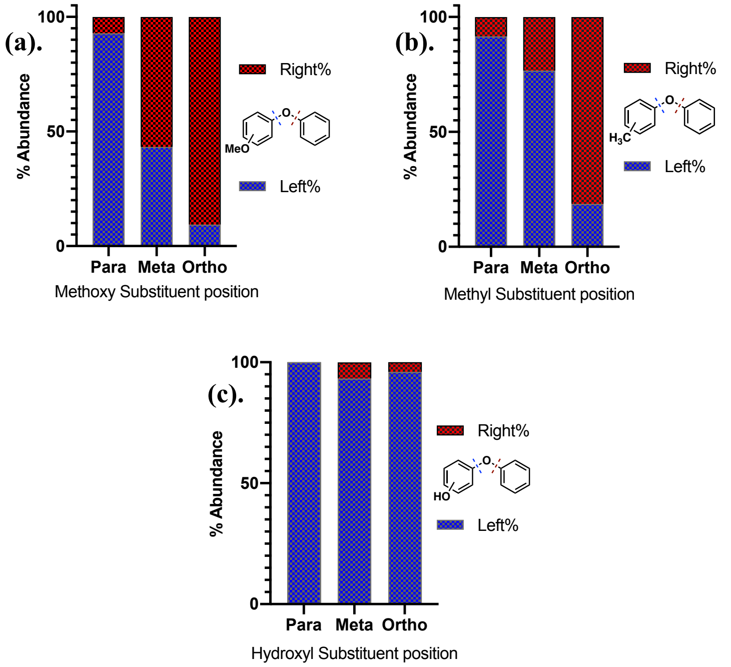

Supplementary Figure 31. Regioselectivity distribution of para, meta and ortho substituted structures of: (a) methoxylated diphenyl ether (b) methylated diphenyl ether (c) hydroxylated diphenyl ether. For additional time courses and product analyses see *Supplementary Fig. 25*, *26* and *27*.

Clearly different from both methoxylated and methylated models, the cleavage regioselectivity of hydroxylated diphenyl ether was not altered by changing the R group's position. This finding suggests different cleavage mechanisms between the hydroxylated DPE and the other two functionalized substrates.

H/D Exchange of Substituent Position Studies

Fitting Results: %D Incorporation of Methylated Diphenyl Ethers:

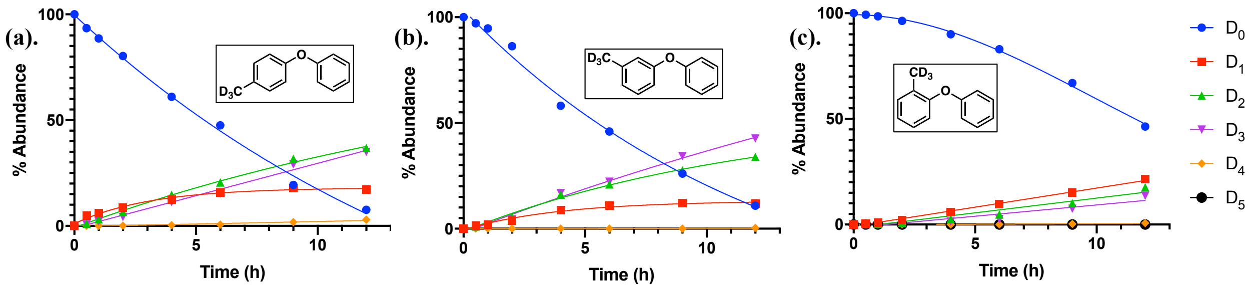

Supplementary Figure 32. Quantified % abundance of D_n of (a) para-methylated diphenyl ether, (b) meta-methylated diphenyl ether and (c) ortho-methylated diphenyl ether. Isotope distributions were determined by GC-MS. The exchange locations were determined by NMR. Spectra of the para isomer are shown above (*Supplementary Figs. 16, 18*); spectra of the meta (MS *Supplementary Fig. 33*, NMR *Supplementary Fig. 35*) and ortho (MS *Supplementary Fig. 37*, NMR *Supplementary Fig. 39*) isomers are shown below.

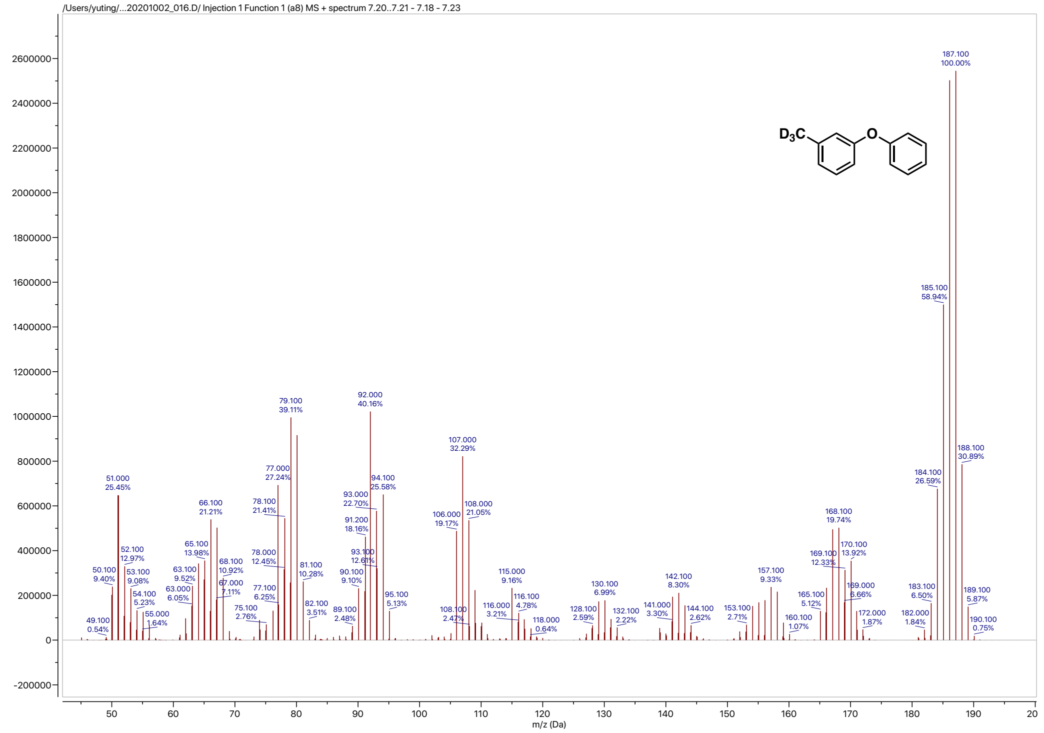

Supplementary Figure 33. Mass spectrum of **labeled** 3-phenoxytoluene after 12 hours H/D exchange. The location of the exchange was further confirmed by NMR.

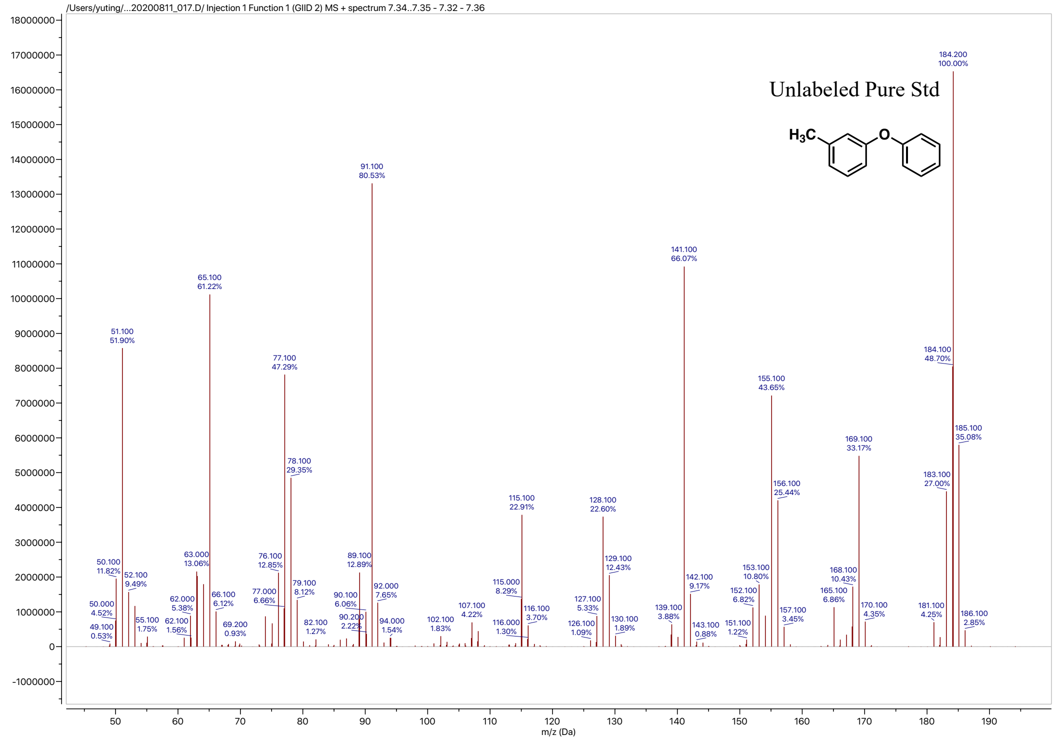

Supplementary Figure 34. Mass spectrum of **unlabeled** 3-phenoxytoluene.

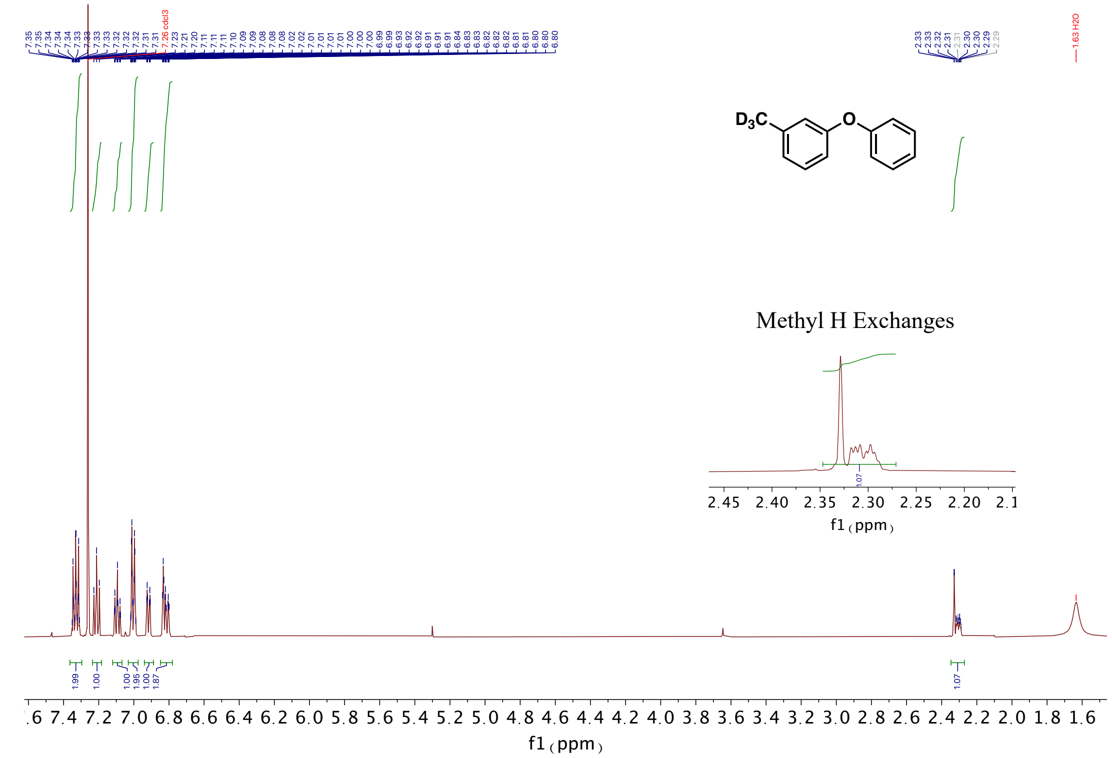

Supplementary Figure 35. NMR spectrum of **labeled** 3-phenoxytoluene after 12 hours exchange. As with the para methyl model, the benzylic methyl showed significant of D incorporation.

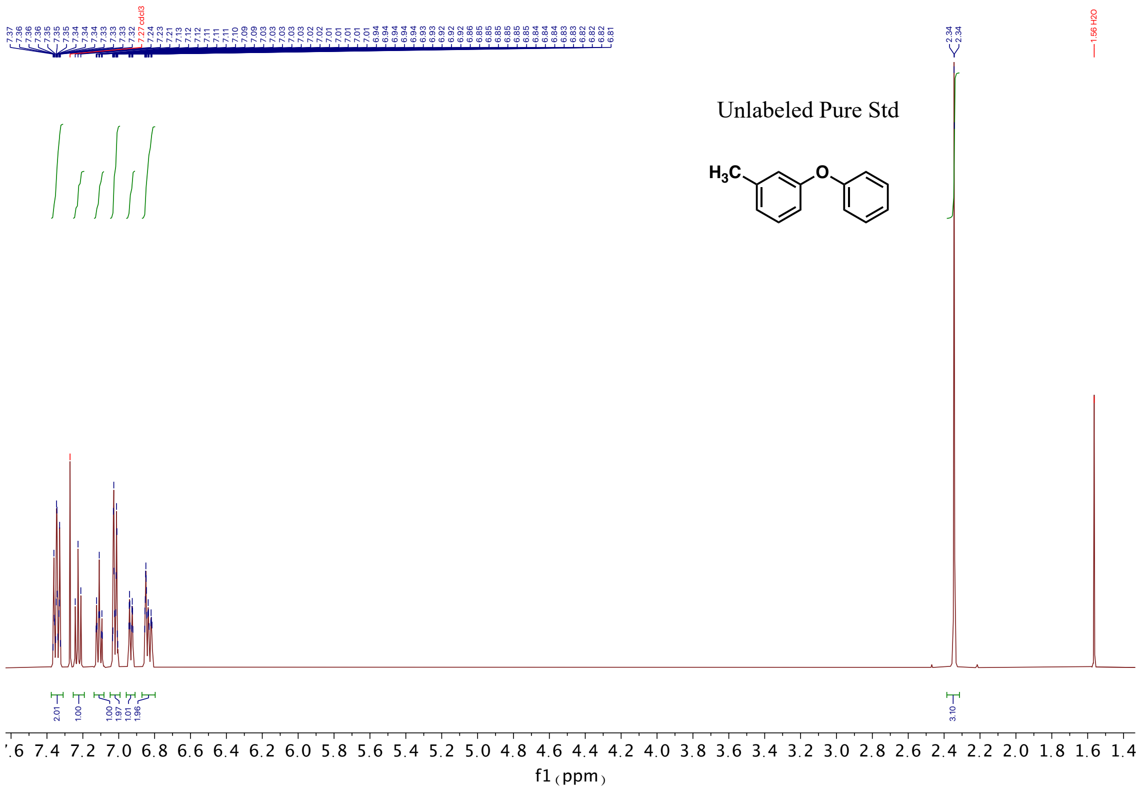

Supplementary Figure 36. NMR spectrum of **unlabeled** 3-phenoxytoluene.

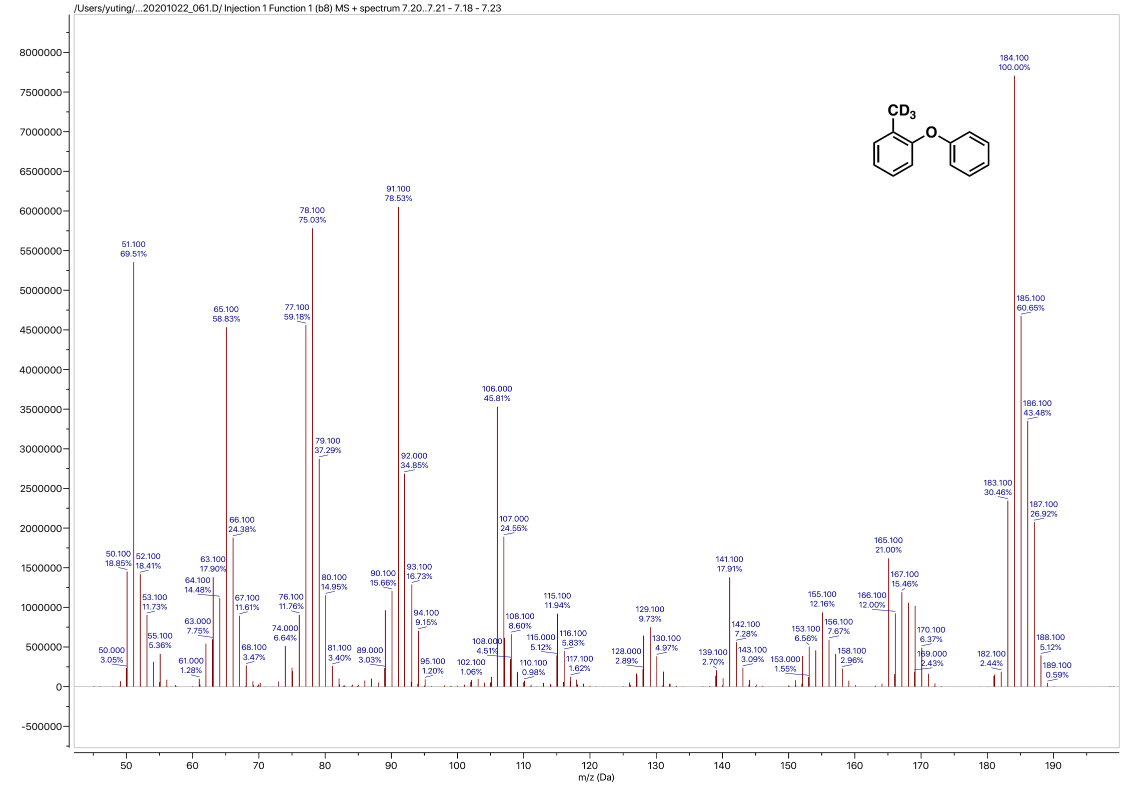

Supplementary Figure 37. Mass spectrum of **labeled** 2-phenoxytoluene after 12 hours H/D exchange. The location of the exchange was further confirmed by NMR.

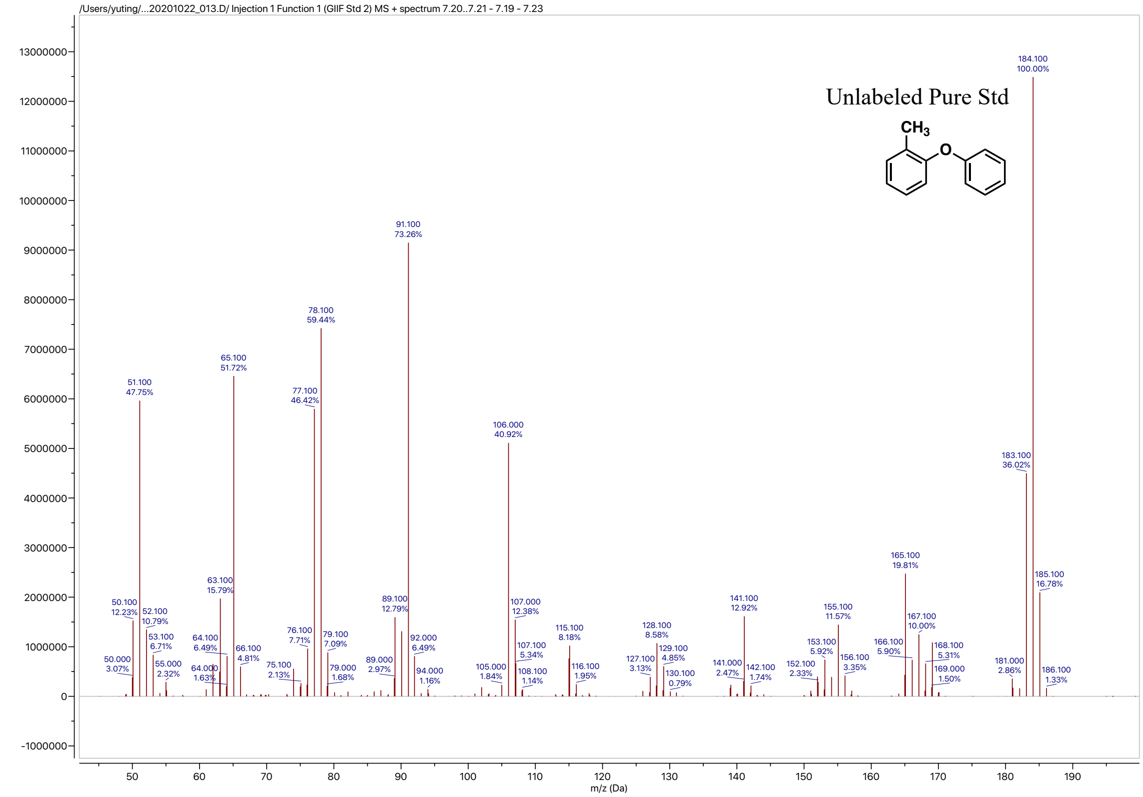

Supplementary Figure 38. Mass spectrum of **unlabeled** 2-phenoxytoluene.

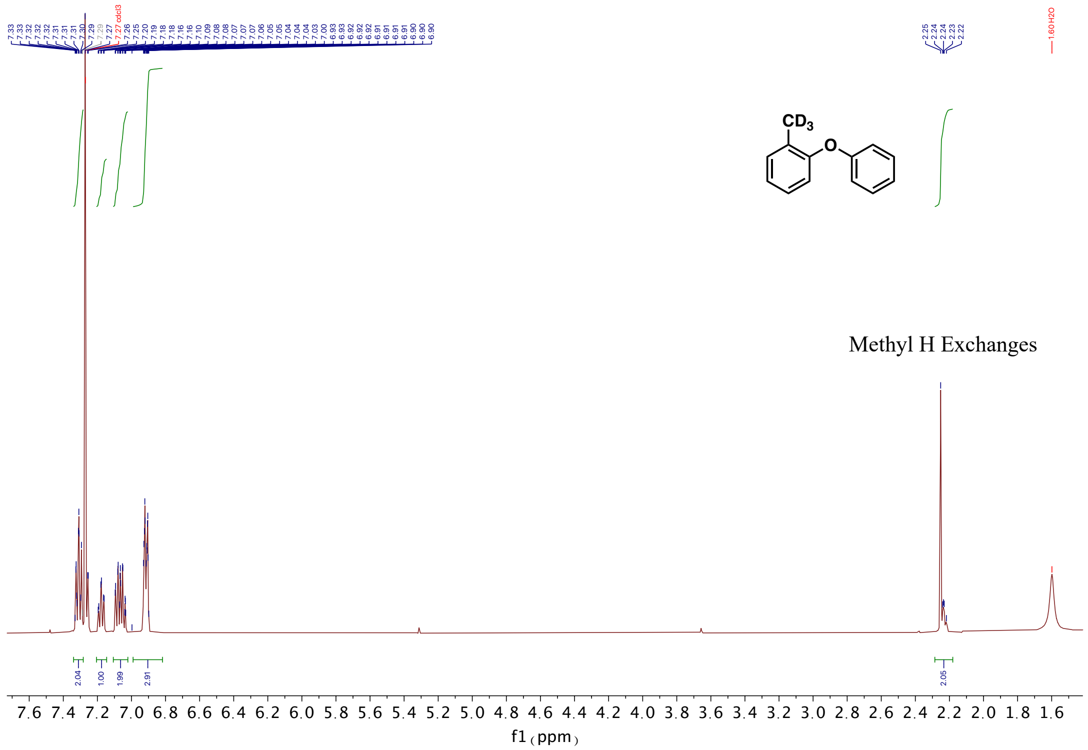

Supplementary Figure 39. NMR spectrum of **labeled** 2-phenoxytoluene after 12 hours exchange. Though the exchange is much slower than in the para and meta methyl models, the benzylic methyl still has observable D incorporation.

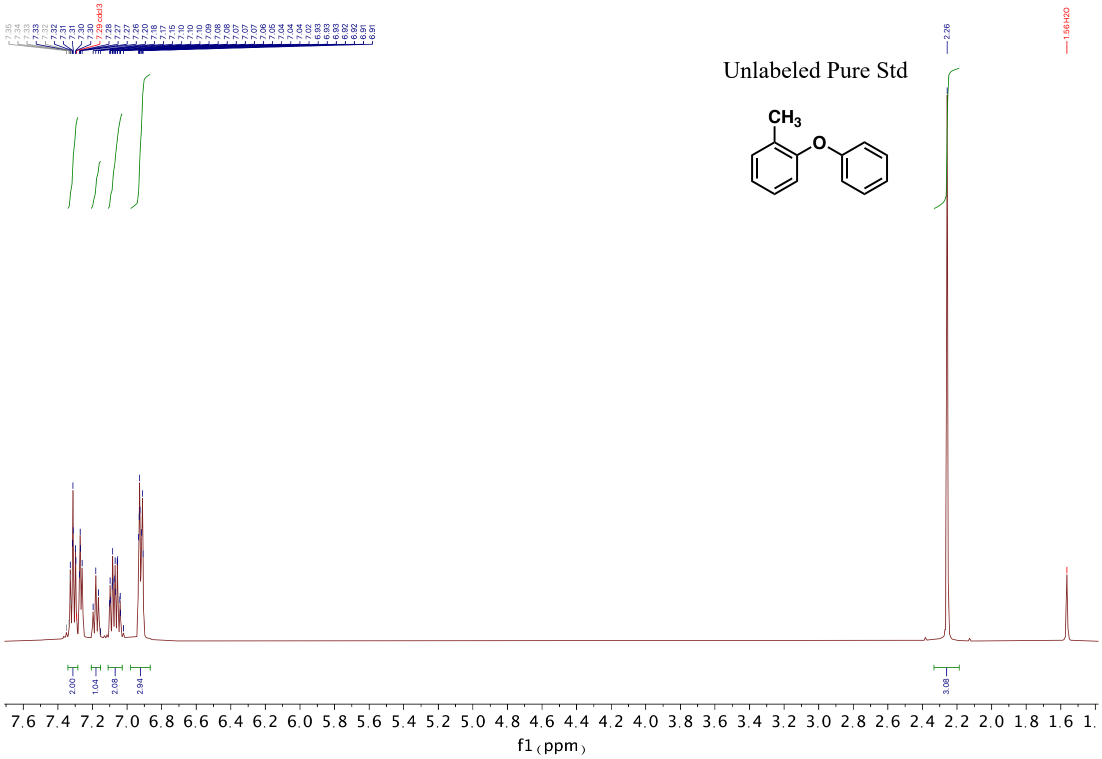

Supplementary Figure 40. NMR spectrum of **unlabeled** 2-phenoxytoluene.

Fitting Results: %D Incorporation of Methoxylated Diphenyl Ethers:

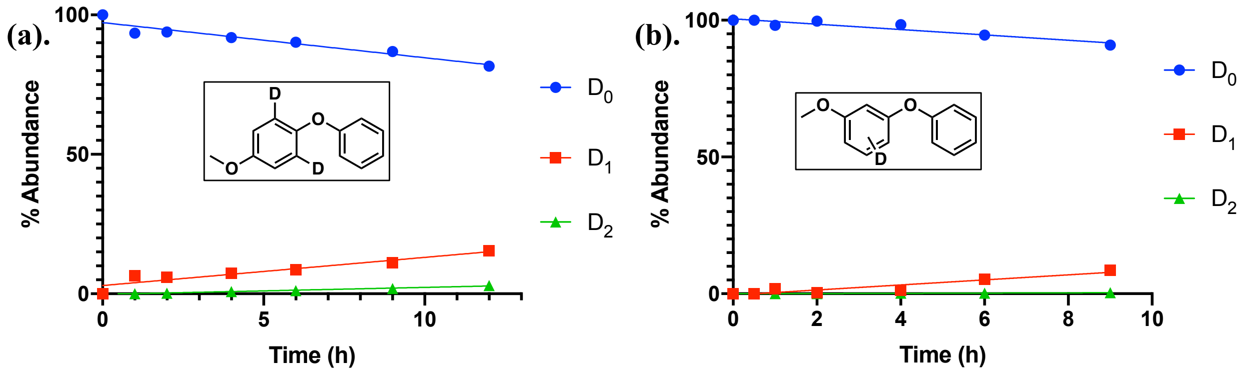

Supplementary Figure 41. Quantified % abundance of D_n of (a) para-methoxylated diphenyl ether and (b) meta-methoxylated diphenyl ether. Isotope distributions were determined by GC-MS. The exchange locations were determined by NMR. Spectra of the para isomer are shown above (*Supplementary Figs. 11*, *13*), spectra of the meta isomer are shown (MS *Supplementary Fig. 42*, NMR *Supplementary Fig. 44*) below.

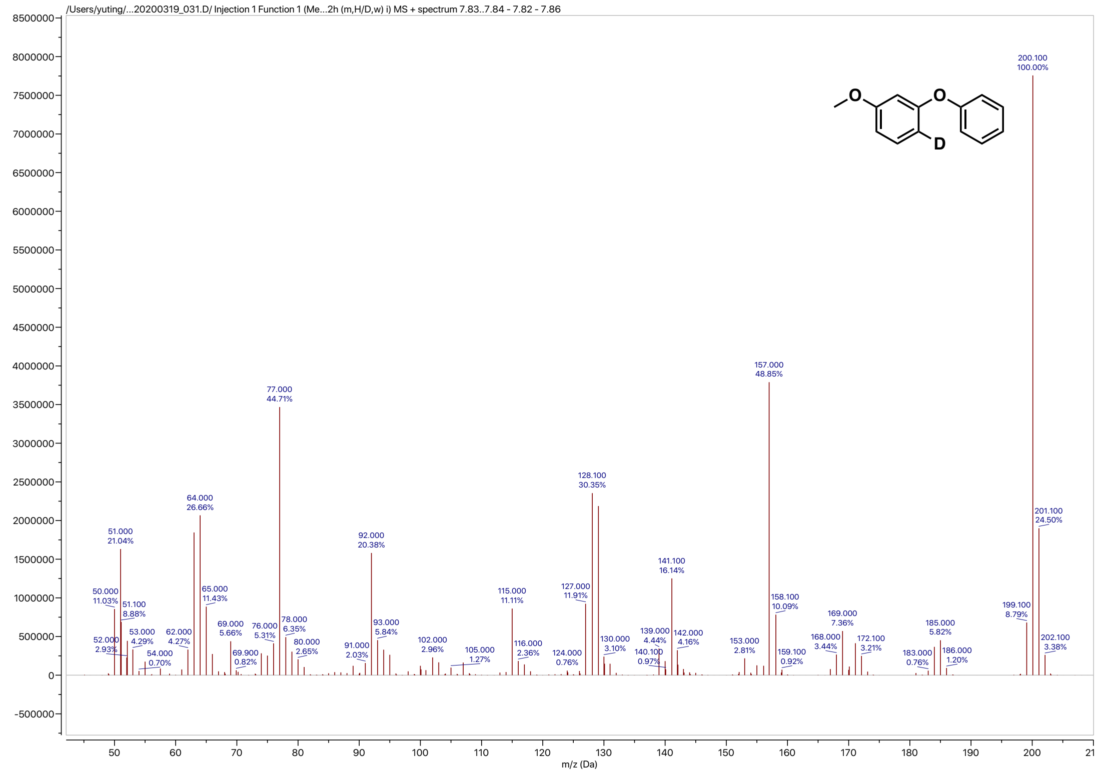

Supplementary Figure 42. Mass spectrum of **labeled** 3-phenoxyanisole after 12 hours H/D exchange. Compared to the spectra of the unlabeled standard (S39), only small increases of m/z 201 and 202, and the decreases in m/z 199 are visible. Same for the NMR spectra below.

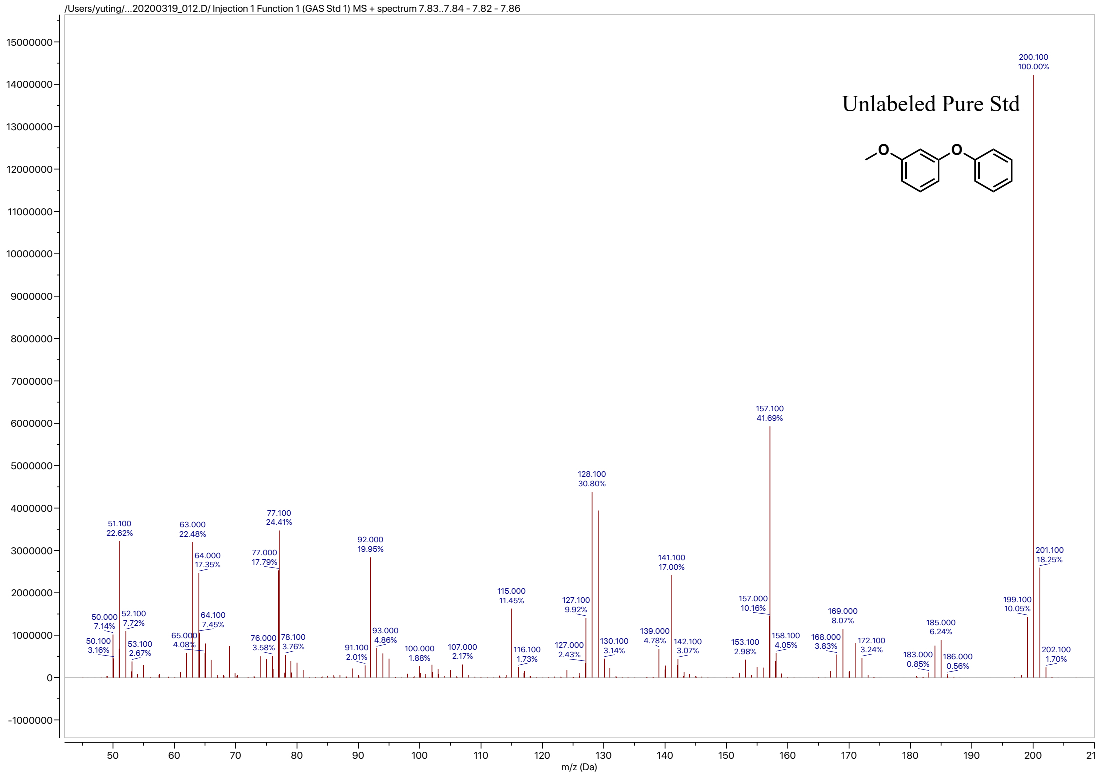

Supplementary Figure 43. Mass spectrum of **unlabeled** 3-phenoxyanisole.

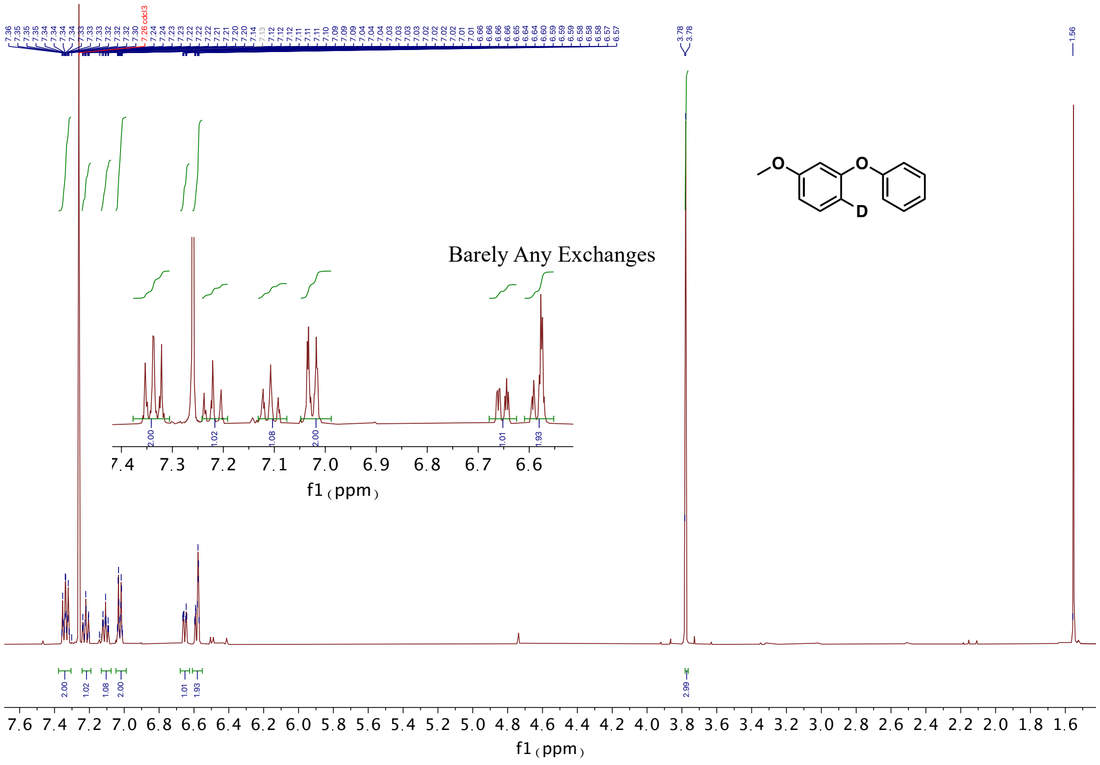

Supplementary Figure 44. NMR spectrum of **labeled** 3-phenoxyanisole after 12 hours exchange. Barely any difference from the unlabeled standard (*Supplementary Fig. 42*) were detected.

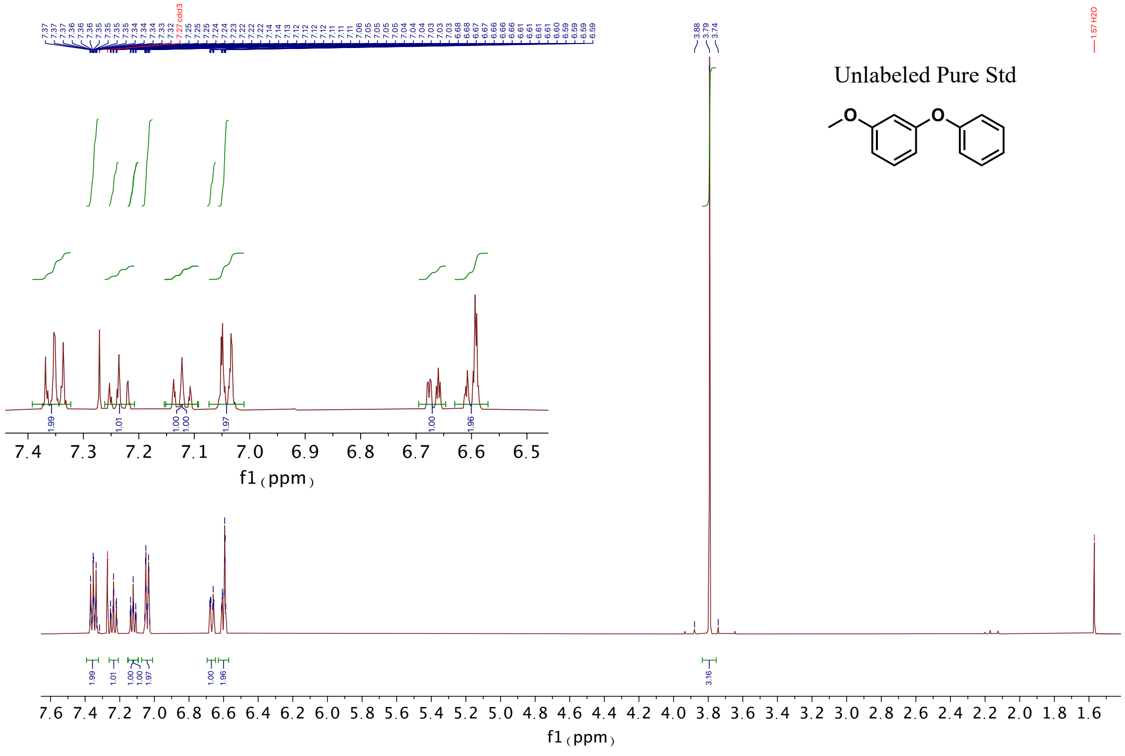

Supplementary Figure 45. NMR spectrum of **unlabeled** 3-phenoxyanisole.

Fitting Results: %D Incorporation of Hydroxylated Diphenyl Ethers:

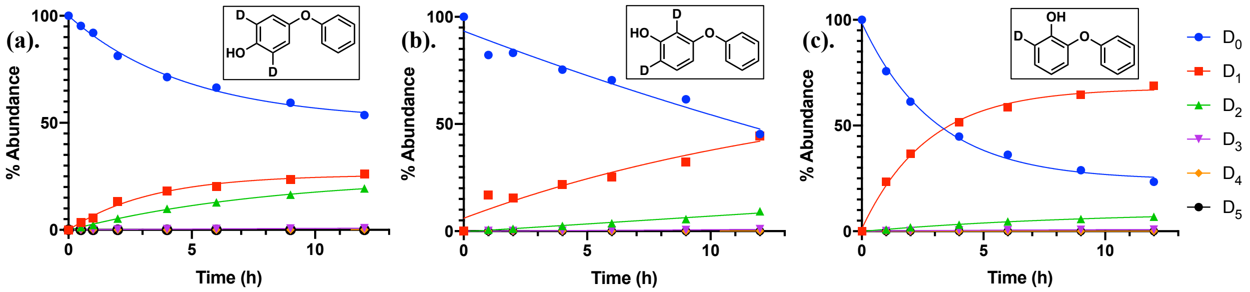

Supplementary Figure 46. Quantified % abundance of D_n of (a) para-hydroxylated diphenyl ether, (b) meta-hydroxylated diphenyl ether and (c) ortho-hydroxylated diphenyl ether. Isotope distributions were determined by GC-MS. The incorporated D on hydroxyl is easy to lose during the preparation of the analytical sample, therefore, the MS analysis samples had an extra wash using non-deuterated water to remove the OD. The exchange location is determined by NMR. Spectra of the para isomer are shown above (*Supplementary Figs. 21, 23*), spectra of the meta (MS *Supplementary Fig. 47*, NMR *Supplementary Fig. 49*) and ortho (MS *Supplementary Fig. 51*, NMR *Supplementary Figs. 53, 55*) isomers are shown below.

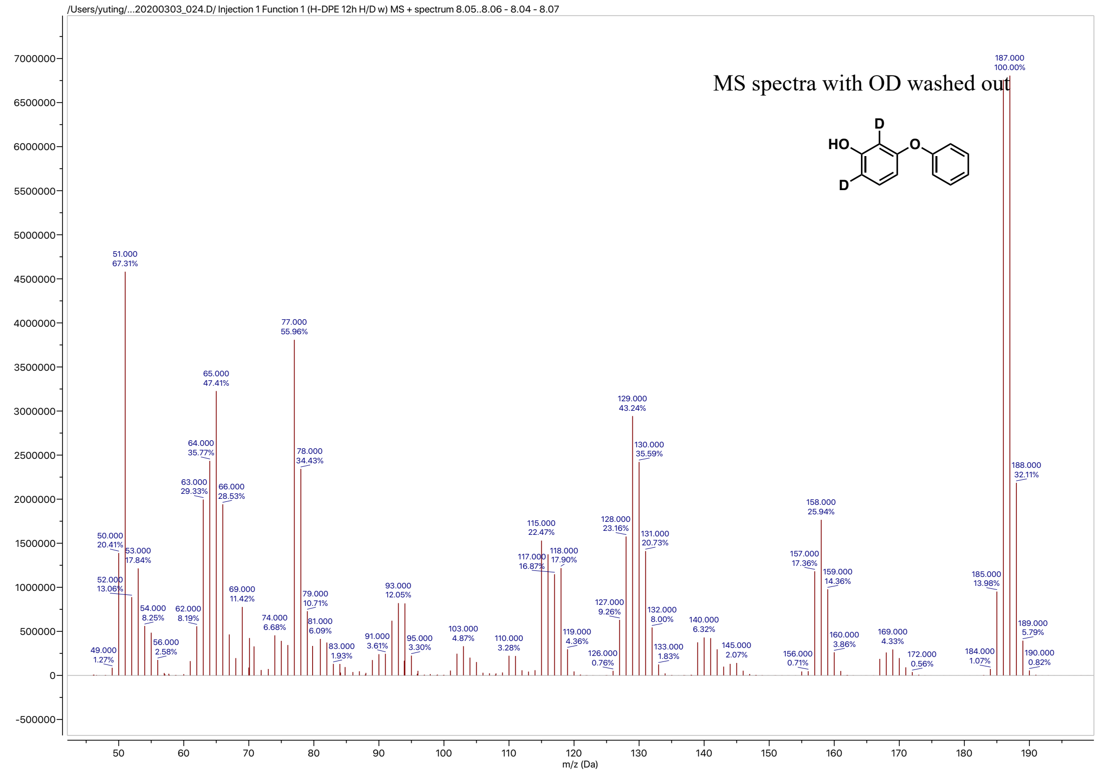

Supplementary Figure 47. Mass spectrum of **labeled** 3-phenoxyphenol after 12 hours H/D exchange. The deuterium on the hydroxyl group was washed off by non-deuterated DI water. The location of the exchange was further confirmed by NMR.

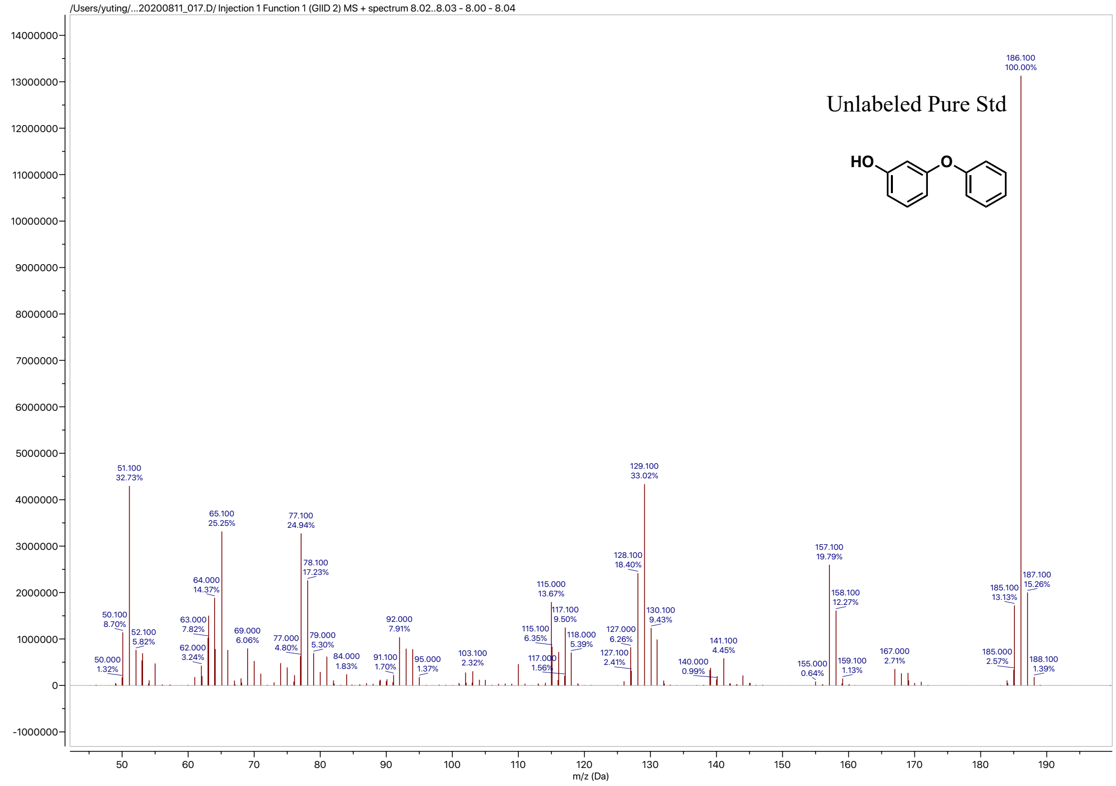

Supplementary Figure 48. Mass spectrum of **unlabeled** 3-phenoxyphenol.

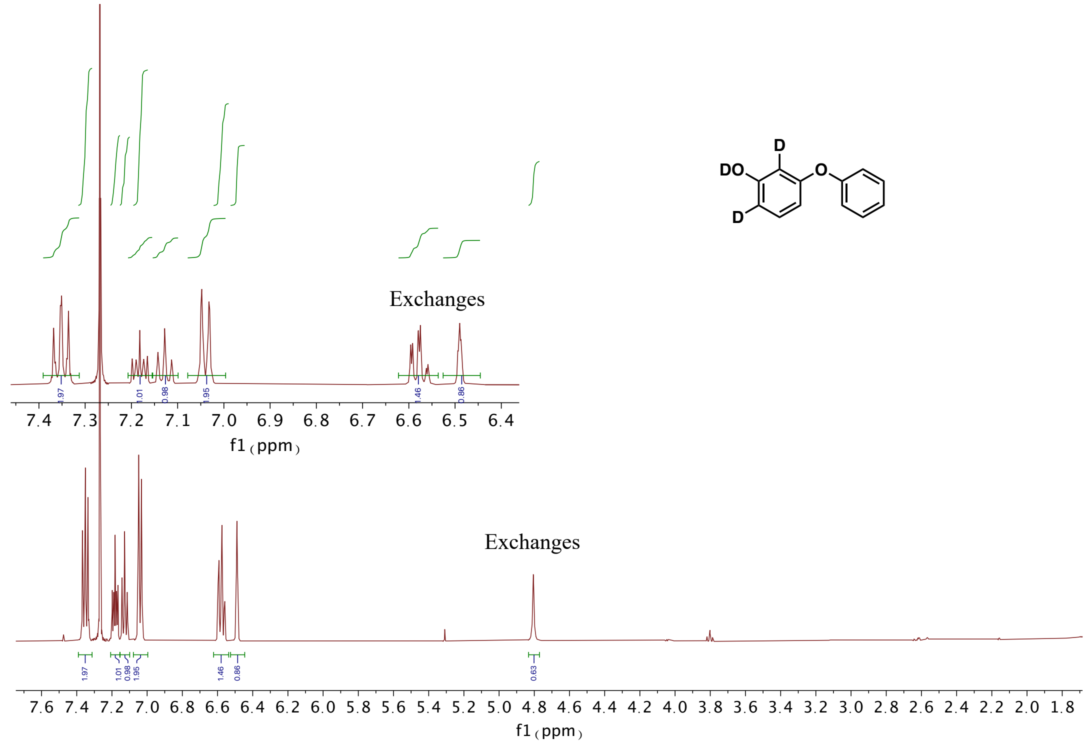

Supplementary Figure 49. NMR spectrum of **labeled** 3-phenoxyphenol after 12 hours exchange. In ^{1}H NMR analysis, OD peak was separated from the aromatic protons, no DI water wash was needed.

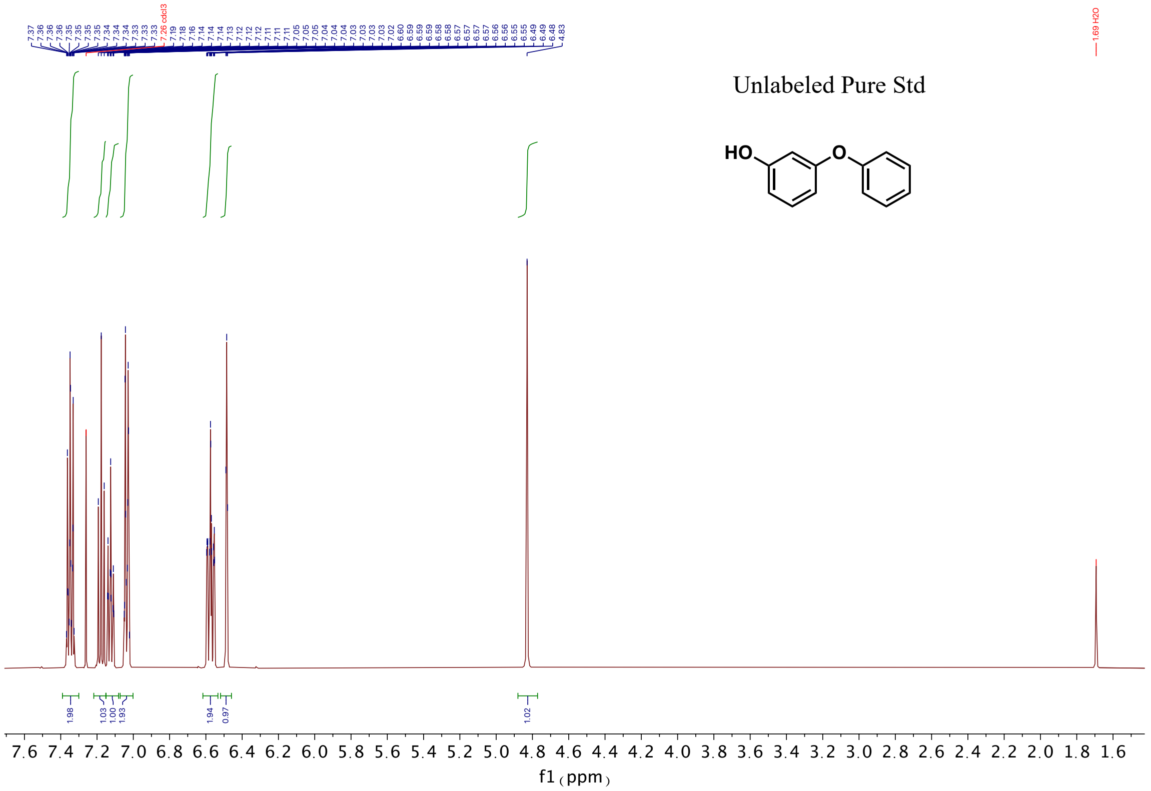

Supplementary Figure 50. NMR spectrum of **unlabeled** 3-phenoxyphenol.

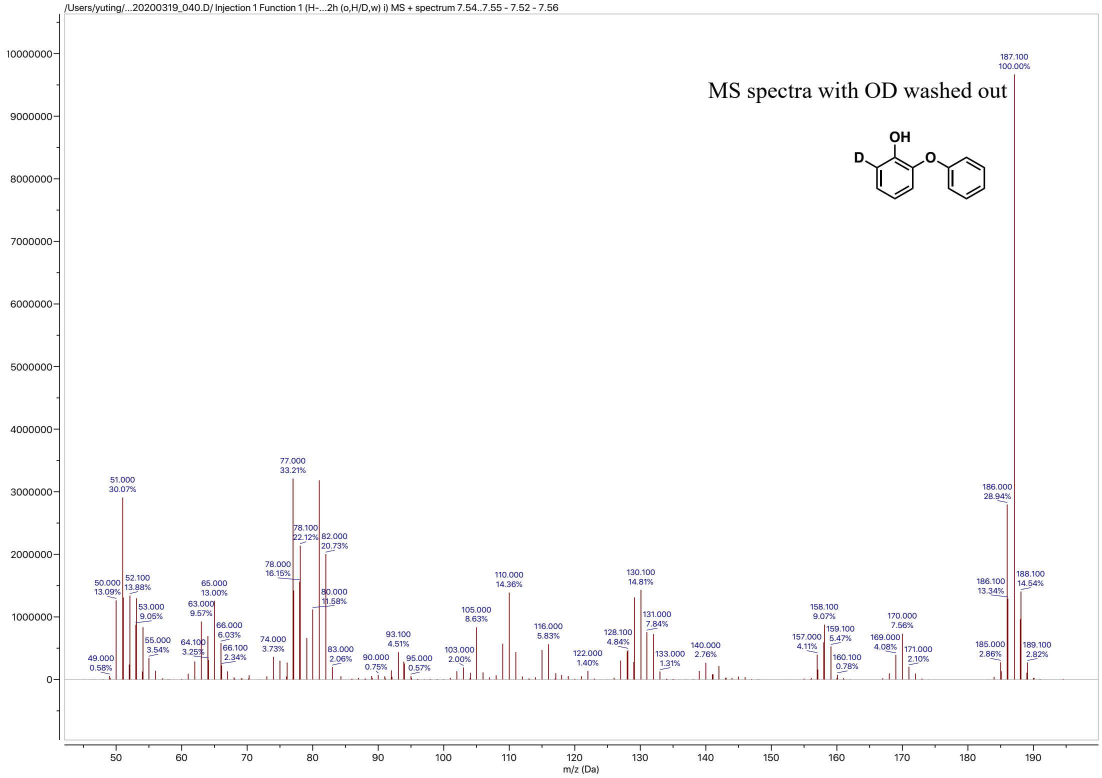

Supplementary Figure 51. Mass spectrum of **labeled** 2-phenoxyphenol after 12 hours H/D exchange. The deuterium on the hydroxyl group was washed off by non-deuterated DI water. The location of the exchange was further confirmed by NMR.

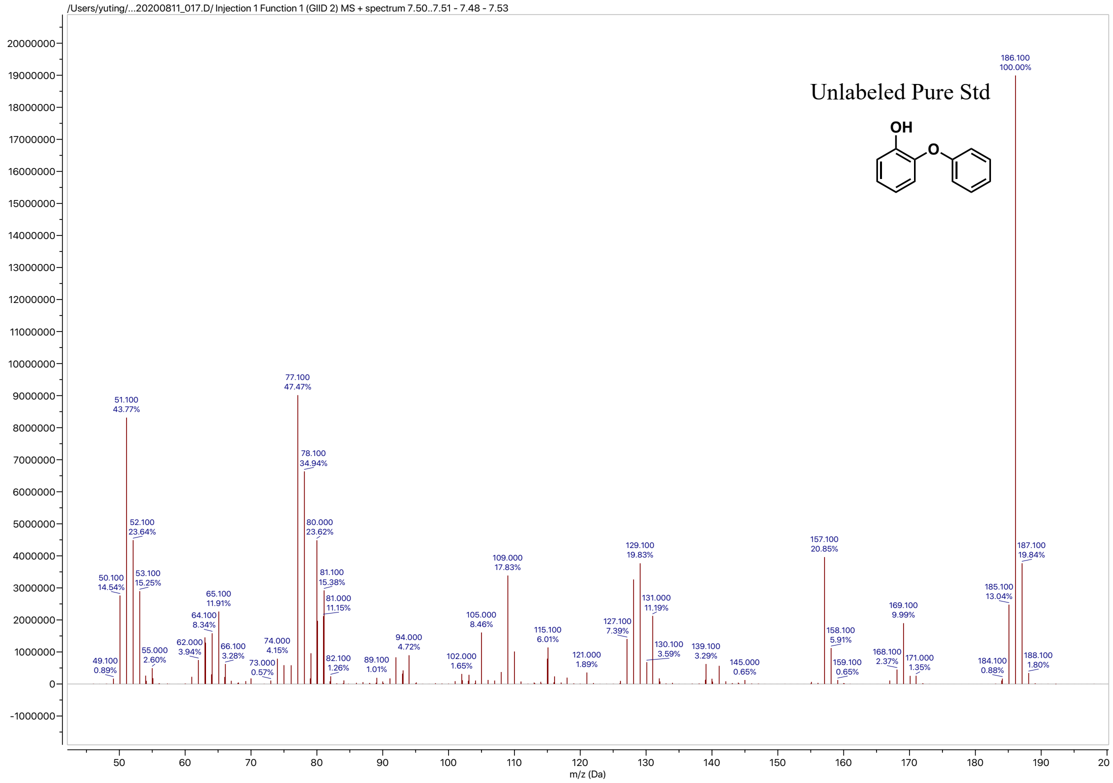

Supplementary Figure 52. Mass spectrum of **unlabeled** 2-phenoxyphenol.

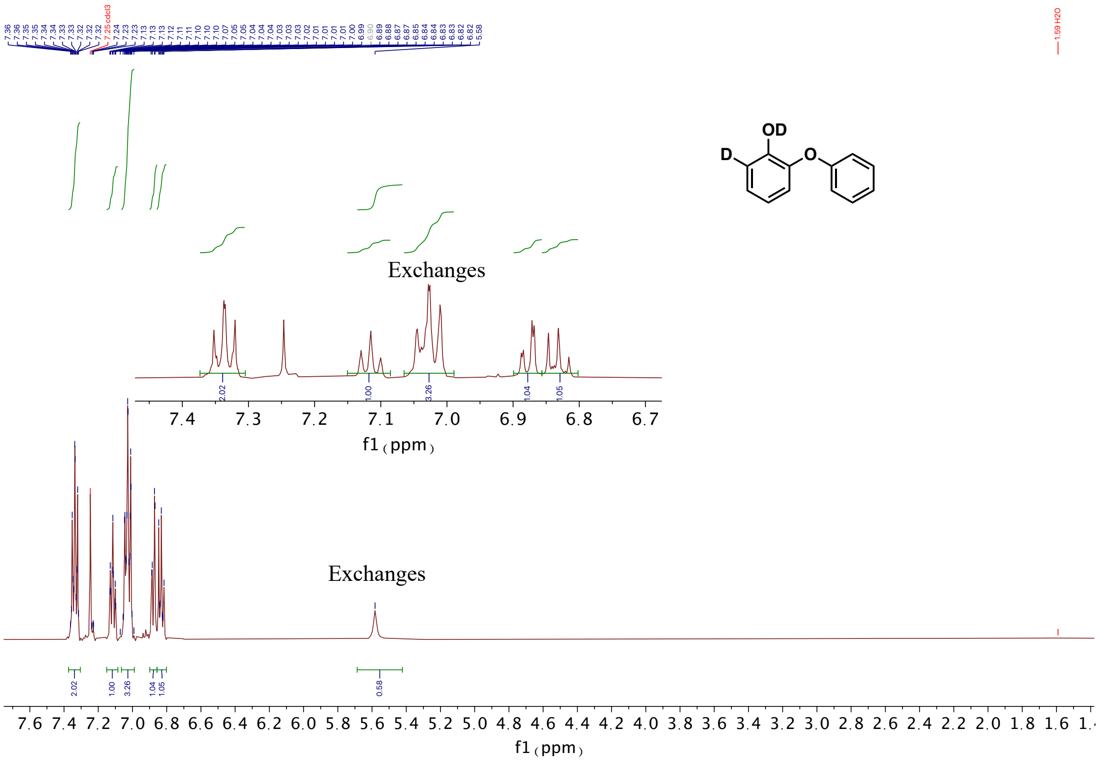

Supplementary Figure 53. NMR spectrum of **labeled** 2-phenoxyphenol after 12 hours exchange. In ^{1}H NMR analysis, OD peak was separated from the aromatic protons, no DI water wash was needed. The exchanged aromatic peak contains different Hs overlapping, to further identify the exchange location, ^{13}C NMR were taken.

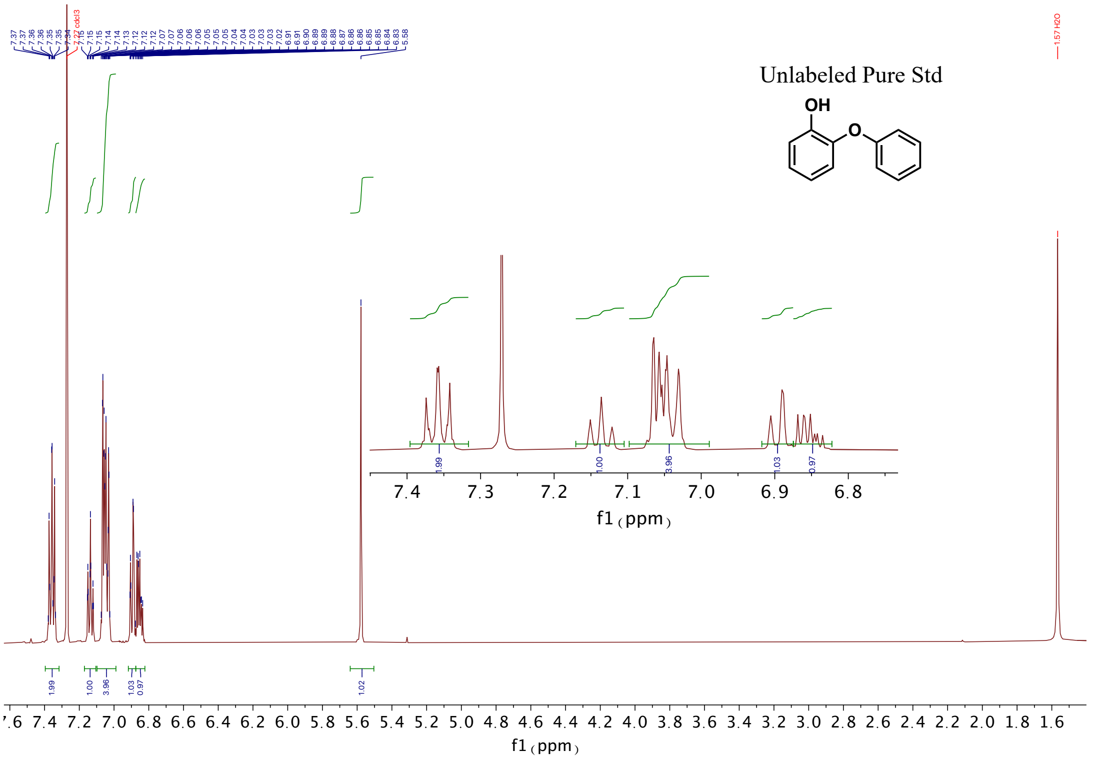

Supplementary Figure 54. NMR spectrum of **unlabeled** 2-phenoxyphenol.

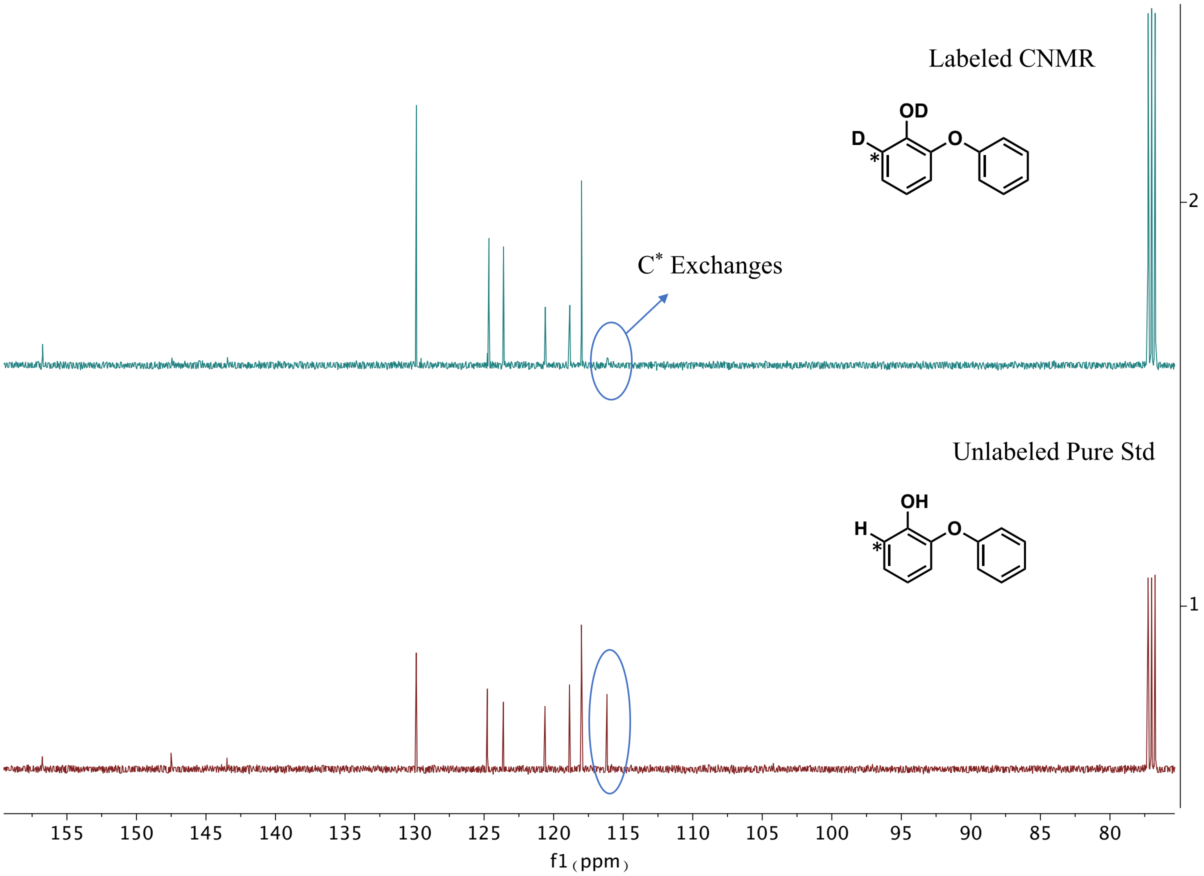

Supplementary Figure 55. Carbon NMR spectrum of **labeled** 2-phenoxyphenol after 12 hours exchange, and the pure unlabeled standard as reference. As indicated in the spectra, the most upfield carbon had a significant decrease of its intensity. Assignments of ^{13}C resonances were made with the aid of NMR chemical shifts computed at the EDF2/6-31+G* level of theory[4] as implemented in the Spartan '18 code.[16] This DFT method has been specifically optimized to cost effectively predict spectroscopic results.

Full ECH Time Courses for Diphenyl Ether Cleavage using Different Organic Co-Solvents

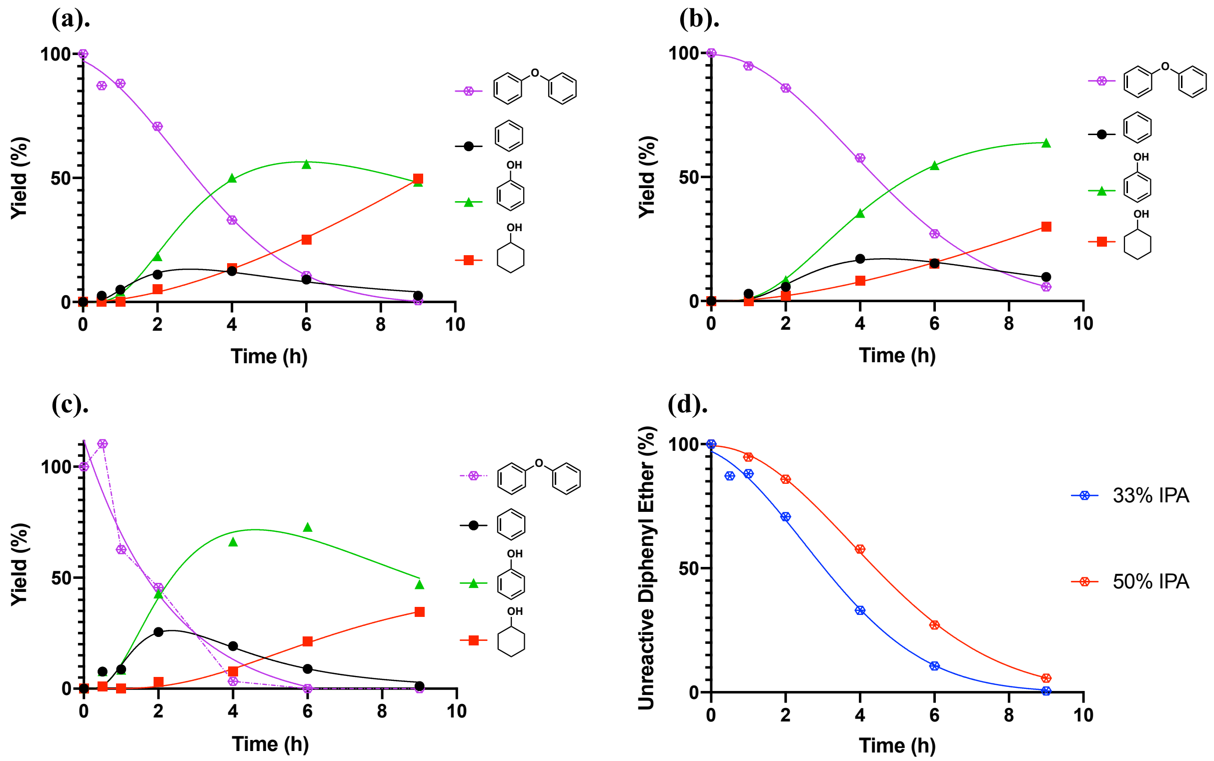

Supplementary Figure 56. Full ECH measurements of (a) diphenyl ether under standard conditions (50 mA, 60 °C) with 33% v/v IPA as co-solvent, (b) diphenyl ether under reaction condition (50 mA, 60 °C) with 50% v/v IPA as co-solvent, (c) diphenyl ether under reaction condition (50 mA, 60 °C) with 33% v/v EtOH as co-solvent, (d) plot of diphenyl ether cleavage rate comparison between 33% v/v IPA and 50% v/v IPA. The yields of cyclohexanol and phenol were corrected for extraction losses (Fig. S1d). No yield corrections were applied on benzene and the starting materials. Notice, 33% v/v EtOH buffer mixture can also achieve complete cleavage, but it's less amphiphilic than IPA, making the starting material diphenyl ether less soluble, which led to fluctuations in the quantification at early time points during the analysis (as highlighted by the dotted line).

Full Time Courses for ECH of 4-phenoxyphenol (Hydroxylated DPE) using Different Organic Co-Solvents at Different Time Scales

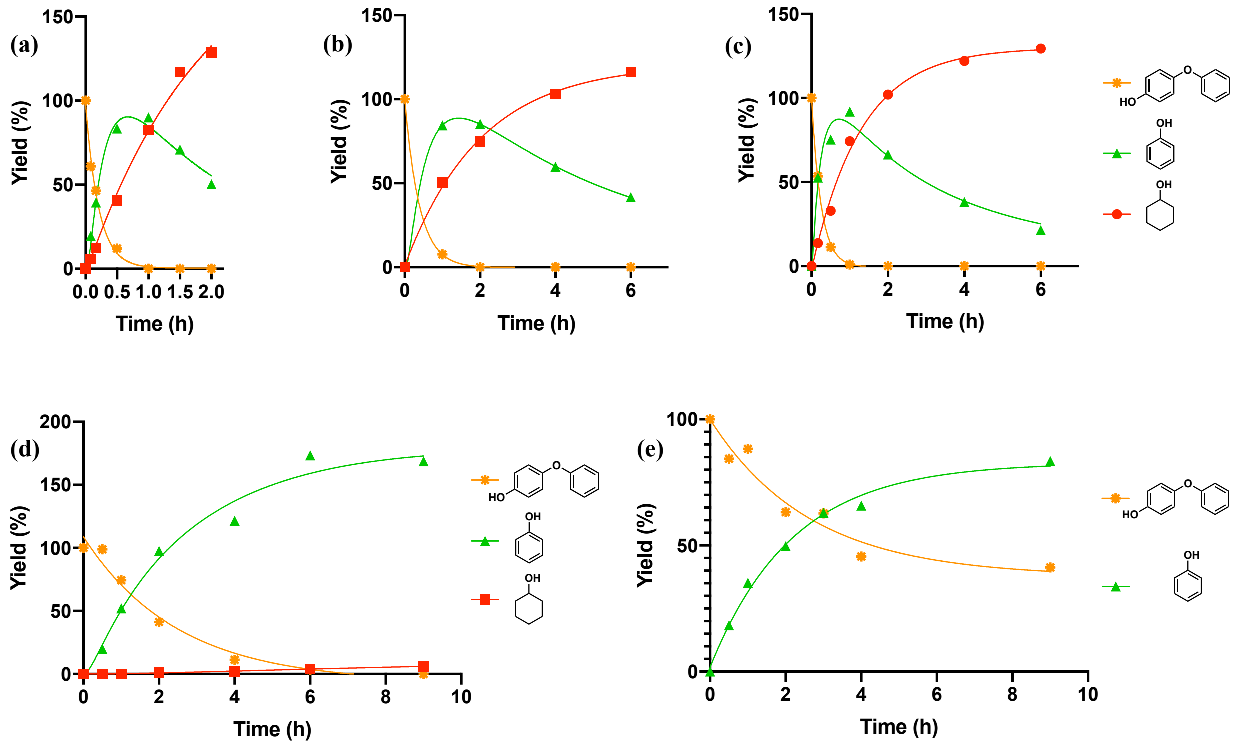

Supplementary Figure 57. Full ECH measurements of 4-phenoxyphenol under standard conditions (50 mA, 60 °C) with (a) 100% pure buffer, (b) 10% v/v IPA as co-solvent, (c) 10% v/v EtOH as co-solvent, (d) 5% acetone as co-solvent and (e) 10% acetone as co-solvent. Low percentage of organic co-solvent were used, no yield correction was applied.

Plotted Comparison of Acetone Inhibitory Effects: DPE vs. HO-DPE

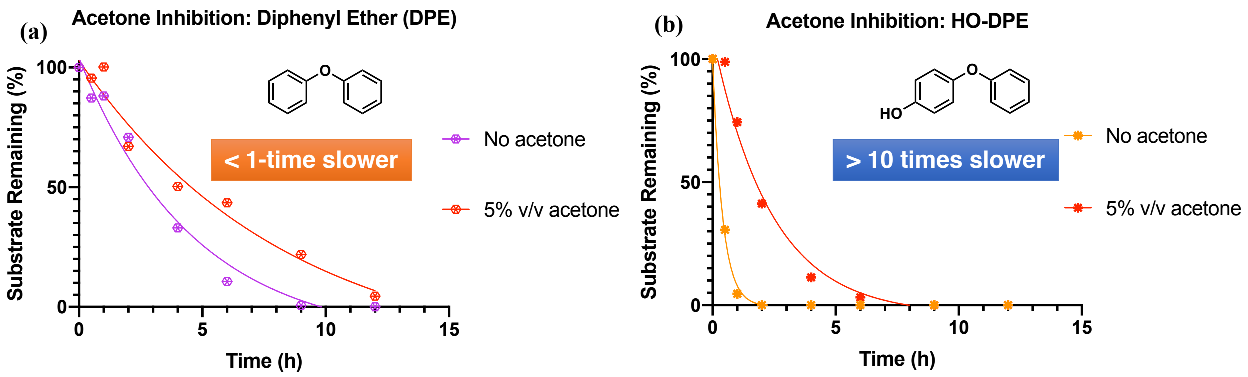

Supplementary Figure 58. (a) C-O cleavage rate inhibition of diphenyl ether by 5% v/v acetone as co-solvent (b) C-O cleavage rate inhibition of para-hydroxylated diphenyl ether by 5% v/v acetone as co-solvent.

Extraction Efficiency of Other Aromatic Products

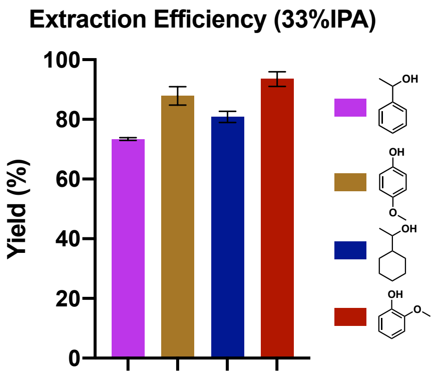

Supplementary Figure 59. Extraction efficiency of other polar aromatic products.

ECH of Diphenyl Ether Using Reticulated Vitreous Carbon (RVC) Electrode

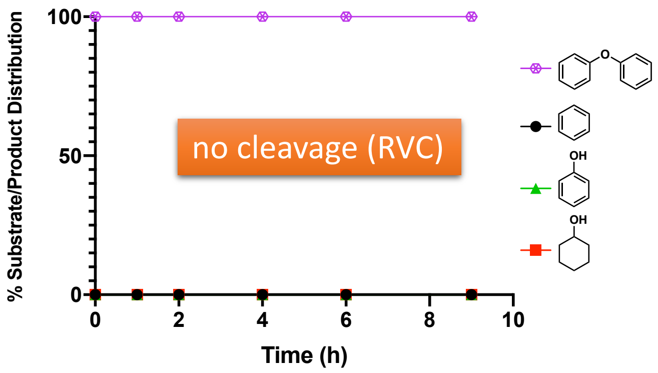

Supplementary Figure 60. ECH measurement of diphenyl ether followed the general ECH experimental procedure in S4 with standard condition (50 mA, ~10 V, 60 °C and 33%IPA) using a reticulated vitreous carbon electrode (8 mA/cm^2). Hydrogen evolution was observed, but none of the diphenyl ethers were cleaved.

Full Time Course for 1-(4-phenoxyphenyl)ethan-1-one ECH

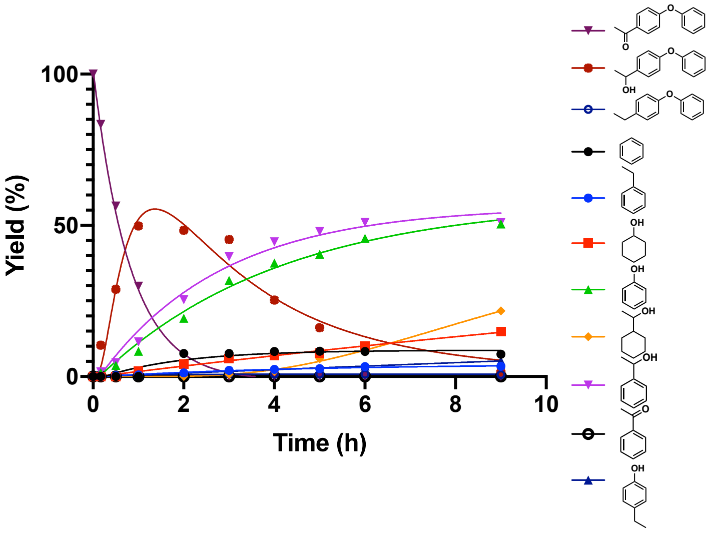

Supplementary Figure 61. ECH measurement of 1-(4-phenoxyphenyl)ethan-1-one at standard condition (50 mA, 60 °C and 33% IPA). The yields of phenol, cyclohexanol, 1-phenylethanol and 1-cyclohexylethanol were corrected for extraction losses (*Supplementary Figs. 1d, 58*). The yield of 4-ethylphenol was corrected using the extraction efficiency of phenol (*Supplementary Fig. 1d*). No yield correction was applied for benzene, ethylbenzene or the starting material 1-(4-phenoxyphenyl)ethan-1-one.

Full Time Course for 1-(4-phenoxyphenyl)ethan-1-ol ECH

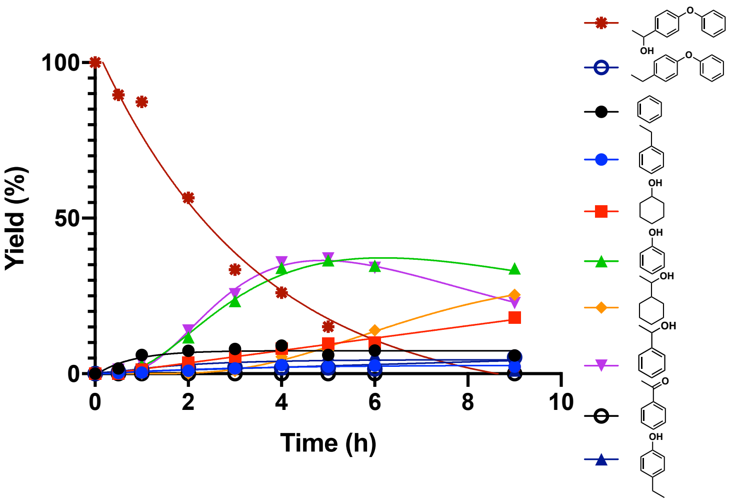

Supplementary Figure 62. ECH measurement of 1-(4-phenoxyphenyl)ethan-1-ol at standard condition (50 mA, 60 °C and 33% IPA). The yields of phenol, cyclohexanol, 1-phenylethanol and 1-cyclohexylethanol were corrected for extraction losses (*Supplementary Figs. 1d, 58*). The yield of 4-ethylphenol was corrected using the extraction efficiency of phenol (*Supplementary Fig. 1d*). No yield correction was applied for benzene, ethylbenzene or the starting material 1-(4-phenoxyphenyl)ethan-1-ol.

Full Time Course for 4-phenoxybenzonitrile ECH

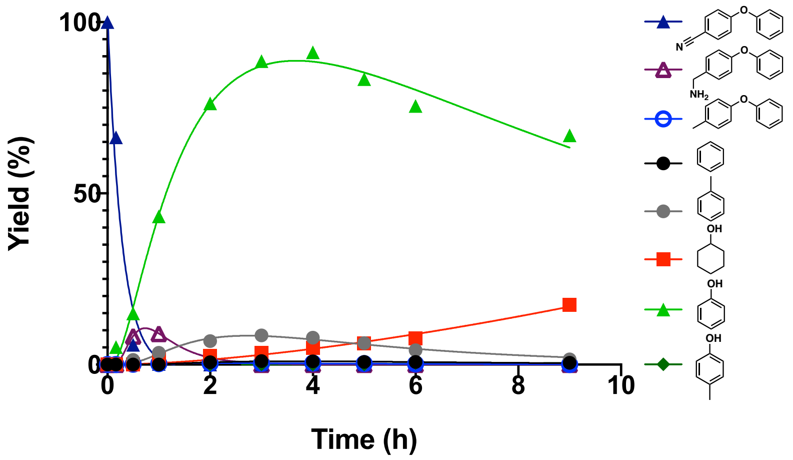

Supplementary Figure 63. ECH measurement of 4-phenoxybenzonitrile at standard condition (50 mA, 60 °C and 33% IPA). The yields of phenol and cyclohexanol were corrected for extraction losses (*Supplementary Fig. 1d*). No yield correction was applied for benzene, toluene, p-cresol or the starting material 4-phenoxybenzonitrile. The more polar primary amine product phenylmethanamine was not quantified due to limitations of the GC column. Importantly, however, no s was detected.

Full Time Course for 4-phenoxybenzaldehyde ECH

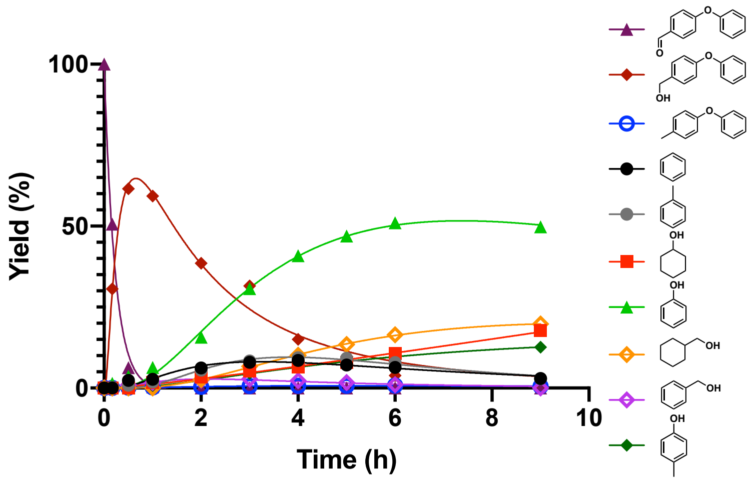

Supplementary Figure 64. ECH measurement of 4-phenoxybenzaldehyde at standard condition (50 mA, 60 °C and 33% IPA). The yields of phenol and cyclohexanol were corrected for extraction losses (*Supplementary Fig. 1d*). The yield of cyclohexylmethanol was corrected using the extraction efficiency of cyclohexanol (*Supplementary Fig. 1d*). The yields of phenylmethanol and p-cresol were corrected using the extraction efficiency of phenol (*Supplementary Fig. 1d*). No yield correction was applied for benzene, toluene or the starting material 4-phenoxybenzaldehyde.

Full Time Course for 1-fluoro-4-phenoxybenzene ECH

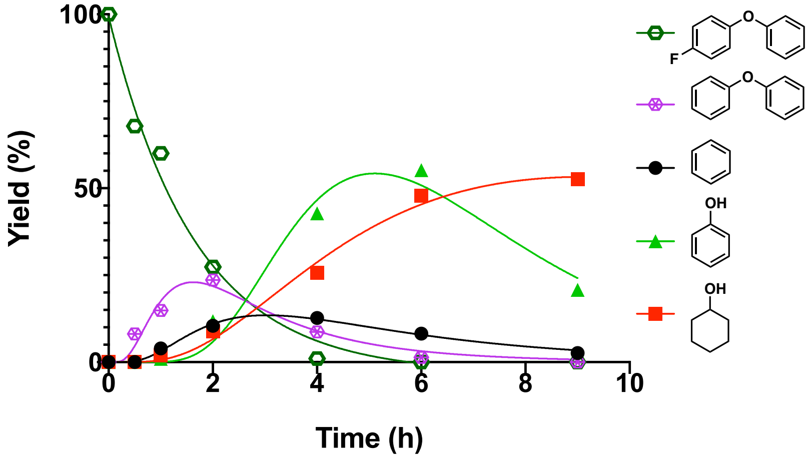

Supplementary Figure 65. ECH measurement of 1-fluoro-4-phenoxybenzene at standard condition (50 mA, 60 °C and 33% IPA). The yields of phenol and cyclohexanol were corrected for extraction losses (*Supplementary Fig. 1d*). No yield correction was applied for benzene, diphenyl ether or the starting material 1-fluoro-4-phenoxybenzene.

Full Time Course for 1-phenoxy-4-(trifluoromethyl)benzene ECH

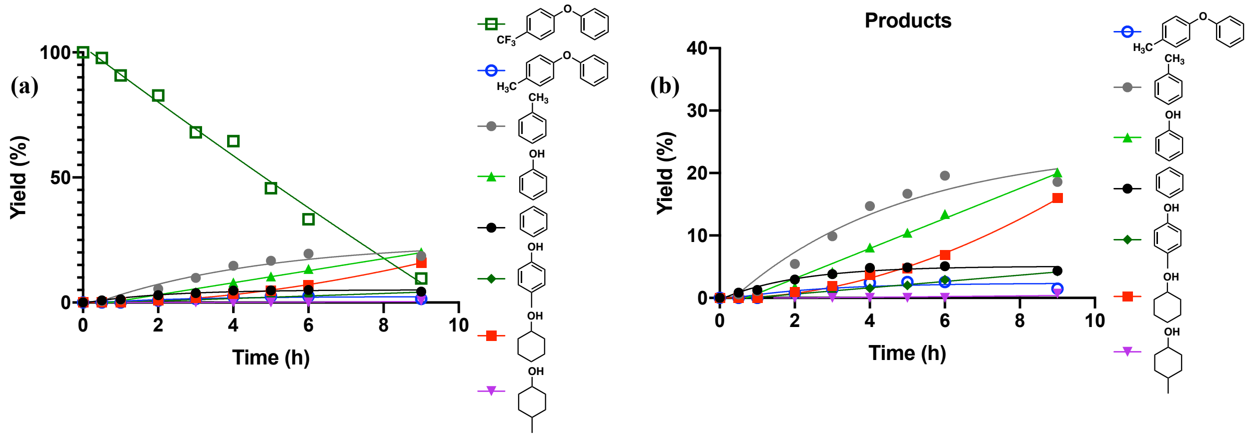

Supplementary Figure 66. (a) ECH measurement of 1-phenoxy-4-(trifluoromethyl)benzene at standard condition (50 mA, 60 °C and 33% IPA). (b) Time courses of product formation. As shown in figure (b), none of the cleavage products contained any F, confirming that defluorination proceeded prior to the C-O cleavage. Yields of phenol and cyclohexanol were corrected for extraction losses (*Supplementary Fig. 1d*). The yield of p-cresol was corrected using the extraction efficiency of phenol (*Supplementary Fig. 1d*). The yield of 4-methylcyclohexanol was corrected using the extraction efficiency of cyclohexanol (*Supplementary Fig. 1d*). No yield corrections were applied for benzene, toluene or the starting material 1-phenoxy-4-(trifluoromethyl)benzene.

Full Time Course for 1-ethyl-4-phenoxybenzene ECH

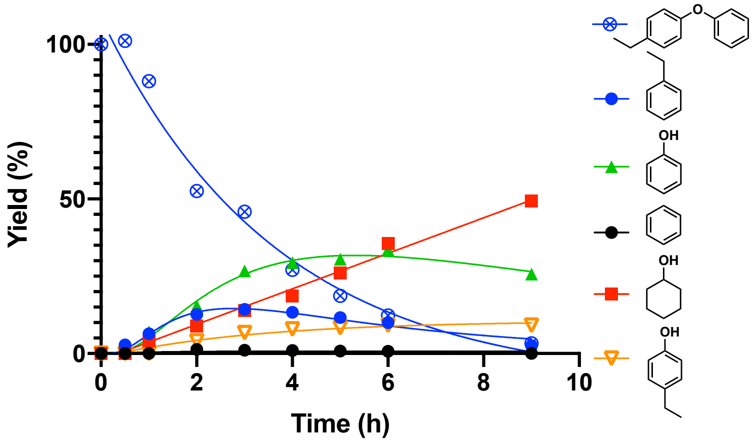

Supplementary Figure 67. ECH measurement of 1-ethyl-4-phenoxybenzene at standard condition (50 mA, 60 °C and 33% IPA). The yields of phenol and cyclohexanol were corrected for extraction losses (*Supplementary Fig. 1d*). The yield of 4-ethylphenol was corrected using the extraction efficiency of phenol (*Supplementary Fig. 1d*). No yield correction was applied for benzene, ethylbenzene or the starting material 1-ethyl-4-phenoxybenzene.

Full Time Course for 1-phenoxy-4-(trifluoromethyl)benzene ECH

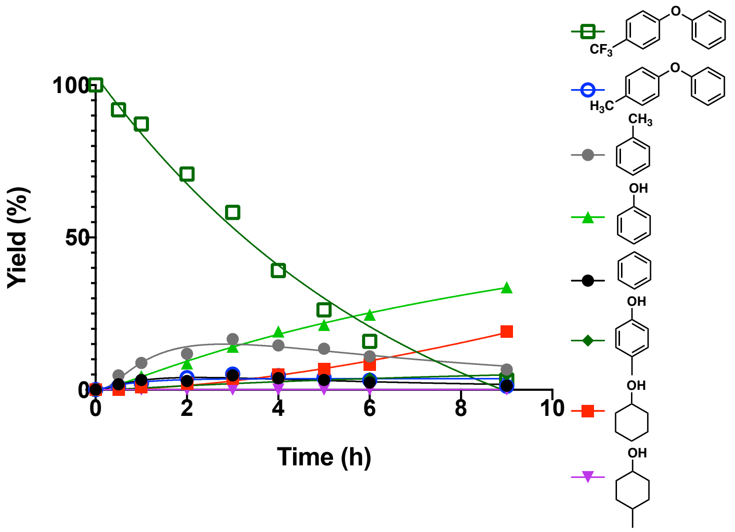

Supplementary Figure 68. ECH measurement of 1-phenoxy-4-(trifluoromethyl)benzene at **80 mA**, 60 °C and 33% IPA. The yields of phenol and cyclohexanol were corrected for extraction losses (*Supplementary Fig. 1d*). The yield of p-cresol was corrected using the extraction efficiency of phenol (*Supplementary Fig. 1d*). The yield of 4-methylcyclohexanol was corrected using the extraction efficiency of cyclohexanol (*Supplementary Fig. 1d*). No yield corrections were applied for benzene, toluene or the starting material 1-phenoxy-4-(trifluoromethyl)benzene. None of the potential fluorinated toluenes were observed.

Full Time Course for 4,4'-oxybis(methoxybenzene) ECH

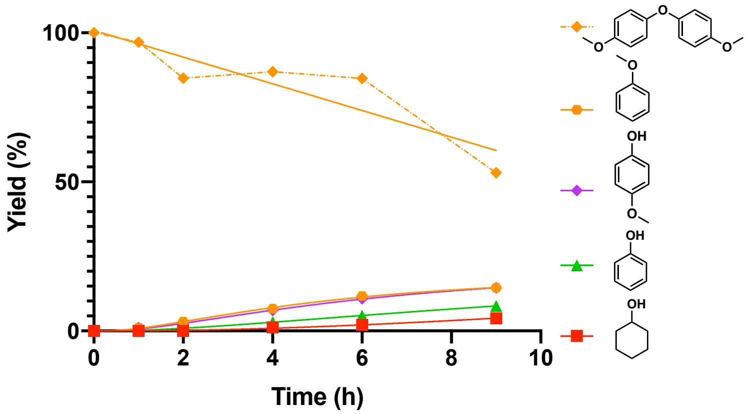

Supplementary Figure 69. ECH measurement of 4,4'-oxybis(methoxybenzene) at standard condition (50 mA, 60 °C and 33% IPA). The yields of phenol, cyclohexanol and anisole were corrected for extraction losses (*Supplementary Fig. 1d*). The yield of 4-methoxyphenol was corrected for extraction losses (*Supplementary Fig. 58*). No yield correction was applied for the starting material 4-phenoxybenzaldehyde.

Kinetic Cleavage Rate Comparison of Different Para-functionalized Diphenyl Ethers

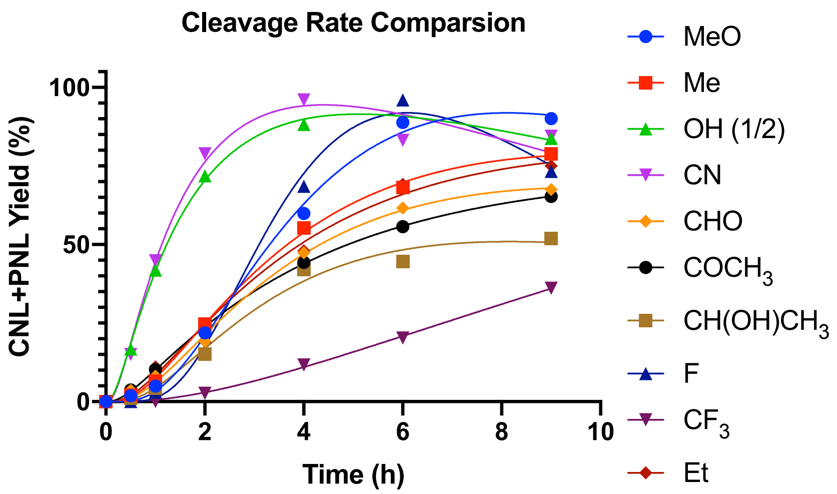

Supplementary Figure 70. Cleavage rate of various para-functionalized diphenyl ethers, under standard ECH condition (50 mA, 60 °C, 33% IPA) was used for all substrates. Note, diphenyl ether with electron withdrawing functional groups undergoes substituent reduction prior to the C-O cleavage; thus, the sum of cleavage products (cyclohexanol (CNL) and phenol (PNL)) was used to represent the actual rate of the cleavage. And since hydroxylated diphenyl ether generates two equivalents of phenol and cyclohexanol per cleavage, 1/2 of the sum of phenol and cyclohexanol was plotted for this rate comparison.

As indicated in the figure, functionalities (OH, CN) which helps enhance aromatic binding to the catalyst surface showed the highest cleavage rate. Fluorinated (F) diphenyl ether undergoes fast defluorination to become diphenyl ether, which had a similar cleavage rate as diphenyl ether (DPE) and the methoxylated (MeO) DPE. The alkyl DPE had a cleavage rate slightly slower than the listed four, but still faster than the other models that experienced functional group reduction before the C-O cleavage. And among those, CF_3 had the slowest cleavage rate. As discussed in the paper, the perfluoralkyl DPE only begins its C-O cleavage after all of the benzylic fluorine were reduced; making it the slowest among those shown.

Substituted Diphenyl Ether Synthesis Procedures

General Synthesis Procedure for Electron-Donating Diphenyl Ethers:

The ether dimers were prepared following a literature procedure with modifications.[5] A 250 mL round bottom flask equipped with water condenser was charged with mixture of 9.8 mmol of iodobenzene, 12.7 mmol of aryl alcohol (1.3 equiv), 0.98 mmol of N,N-dimethylglycine (10 mol%), 0.98 mmol of CuI (10 mol%), 21.5 mmol of Cs_2CO_3 (2.2 equiv) and 100 mL of 1,4-dioxane. The reaction mixture was held at the reflux temperature of 1,4-dioxane for 24 hours under nitrogen atmosphere. Upon completion, the cooled reaction mixtures were filtered to remove the inorganic salt, the filtrate was then poured into 100 mL of DI water and extracted with 2x100 mL of dichloromethane. The combined organic layers were extracted with 2x50 mL of 0.6 M NaOH solution to wash out the excess aryl alcohol, then washed with brine, dried using Na_2SO_4 and concentrated under vacuum. The crude product was purified by column chromatography using DCM/Hexanes (4:1) to give the pure product.

4-phenoxytoluene (Yield 63%): ^{1}H NMR (500 MHz, $CDCl_3$) δ 7.35 – 7.28 (m, 2H), 7.14 (d, *J* = 8.1 Hz, 2H), 7.07 (tt, *J* = 7.4, 1.1 Hz, 1H), 7.02 – 6.96 (m, 2H), 6.96 – 6.88 (m, 2H), 2.34 (s, 3H). Spectral data are in accordance with those previously reported.[5]

3-phenoxytoluene (Yield 35%): ^{1}H NMR (500 MHz, $CDCl_3$) δ 7.38 – 7.32 (m, 2H), 7.23 (t, *J* = 7.8 Hz, 1H), 7.11 (tt, *J* = 7.4, 1.2 Hz, 1H), 7.05 – 7.00 (m, 2H), 6.93 (ddt, *J* = 7.5, 1.6, 0.8 Hz, 1H), 6.87 – 6.80 (m, 2H), 2.34 (d, *J* = 0.7 Hz, 3H). Spectral data are in accordance with those previously reported.[5]

2-phenoxytoluene (Yield 43%): ^{1}H NMR (500 MHz, $CDCl_3$) δ 7.34 – 7.28 (m, 2H), 7.18 (t, *J* = 7.7 Hz, 1H), 7.11 – 7.01 (m, 2H), 6.97 – 6.86 (m, 3H), 2.26 (s, 3H). Spectral data are in accordance with those previously reported.[6]

4-phenoxyanisole (Yield 69%): ^{1}H NMR (500 MHz, $CDCl_3$) δ 7.34 – 7.28 (m, 2H), 7.08 – 7.02 (m, 1H), 7.02 – 6.97 (m, 2H), 6.97 – 6.93 (m, 2H), 6.93 – 6.86 (m, 2H), 3.82 (s, 3H). Spectral data are in accordance with those previously reported.[5]

2-phenoxyanisole (Yield 53%): ^{1}H NMR (500 MHz, $CDCl_3$) δ 7.34 – 7.28 (m, 2H), 7.14 (ddd, *J* = 8.1, 7.3, 1.7 Hz, 1H), 7.06 (tt, *J* = 7.4, 1.1 Hz, 1H), 7.02 (dd, *J* = 8.2, 1.5 Hz, 1H), 7.00 – 6.91 (m, 4H), 3.85 (s, 3H). Spectral data are in accordance with those previously reported.[5]

3-phenoxyanisole (Yield 57%): ^{1}H NMR (500 MHz, $CDCl_3$) δ 7.39 – 7.32 (m, 2H), 7.26 – 7.21 (m, 1H), 7.15 – 7.09 (m, 1H), 7.07 – 7.01 (m, 2H), 6.70 – 6.65 (m, 1H), 6.63 – 6.57 (m, 2H), 3.79 (s, 3H). Spectral data are in accordance with those previously reported.[7]

1-fluoro-4-phenoxybenzene (Yield 63%): ^{1}H NMR (500 MHz, $CDCl_3$) δ 7.37 – 7.29 (m, 2H), 7.09 (tt, *J* = 7.6, 1.1 Hz, 1H), 7.06 – 6.94 (m, 6H). Spectral data are in accordance with those previously reported.[8]

1-ethyl-4-phenoxybenzene (Yield 35%): ^{1}H NMR (500 MHz, $CDCl_3$) δ 7.38 – 7.29 (m, 2H), 7.21 – 7.14 (m, 2H), 7.13 – 7.05 (m, 1H), 7.00 (dd, *J* = 8.7, 1.1 Hz, 2H), 6.95 (d, *J* = 8.5 Hz, 2H), 2.65 (q, *J* = 7.6 Hz, 2H), 1.25 (t, *J* = 7.6 Hz, 3H). Spectral data are in accordance with those previously reported.[9]

General Synthesis Procedure for Electron-Withdrawing Diphenyl Ethers:

The ether dimers were prepared following a literature procedure with modifications.[5] A 250 mL round bottom flask equipped with water condenser was charged with mixture of 9.8 mmol of aryl bromide, 12.7 mmol of phenol (1.3 equiv), 0.98 mmol of N,N-dimethylglycine (10 mol%), 0.98 mmol of CuI (10 mol%), 21.5 mmol of Cs_2CO_3 (2.2 equiv) and 100 mL of 1,4-dioxane. The reaction mixture was held at the reflux temperature of 1,4-dioxane for 24 hours under nitrogen atmosphere. Upon completion, the cooled reaction mixtures were filtered to remove the inorganic salt, the filtrate was then poured into 100 mL of DI water and extracted with 2x100 mL of dichloromethane. The combined organic layers were extracted with 2x50 mL of 0.6 M NaOH solution to wash out the excess phenol, then washed with brine, dried using Na_2SO_4 and concentrated under vacuum. The crude product was purified by column chromatography using DCM/Hexanes (4:1) to give the pure product.

4-phenoxybenzonitrile (Yield 55%): ^{1}H NMR (500 MHz, $CDCl_3$) δ 7.60 (d, *J* = 8.8 Hz, 2H), 7.42 (dd, *J* = 8.5, 7.4 Hz, 2H), 7.25 – 7.21 (m, 1H), 7.07 (dd, *J* = 8.6, 1.1 Hz, 2H), 7.01 (d, *J* = 8.8 Hz, 2H). Spectral data are in accordance with those previously reported.[8]

1-phenoxy-4-(trifluoromethyl)benzene (Yield 67%): ^{1}H NMR (500 MHz, $CDCl_3$) δ 7.61 – 7.53 (m, 2H), 7.43 – 7.36 (m, 2H), 7.19 (ddd, *J* = 8.5, 6.8, 1.1 Hz, 1H), 7.11 – 6.99 (m, 4H). Spectral data are in accordance with those previously reported.[10]

4-phenoxybenzaldehyde (Yield 38%): ^{1}H NMR (500 MHz, $CDCl_3$) δ 9.92 (s, 1H), 7.88 – 7.79 (m, 2H), 7.45 – 7.37 (m, 2H), 7.23 (ddt, *J* = 8.6, 7.4, 1.2 Hz, 1H), 7.12 – 7.03 (m, 4H). Spectral data are in accordance with those previously reported.[11]

1-(4-phenoxyphenyl)ethan-1-one (Yield 68%): ^{1}H NMR (500 MHz, $CDCl_3$) δ 7.94 (d, *J* = 8.8 Hz, 2H), 7.43 – 7.36 (m, 2H), 7.22 – 7.18 (m, 1H), 7.07 (dt, *J* = 7.8, 1.1 Hz, 2H), 7.00 (d, *J* = 8.8 Hz, 2H), 2.58 (s, 3H). Spectral data are in accordance with those previously reported.[8]

1-(4-phenoxyphenyl)ethan-1-ol: This compound was prepared following a literature procedure.[12] 1-(4-phenoxyphenyl)ethan-1-one (1.02 g, 4.8 mmol) was added to 30 mL MeOH. The mixture was hold at 0 °C in ice bath, $NaBH_4$ (0.18 g, 4.8 mmol) was added in small portions within 1 hour. The reaction was then stirred overnight at room temperature. After the completion of the reaction, saturated NH_4Cl (50 mL) solution was added to the mixture and was extracted with 2x50 mL dichloromethane. The combined organic layers were dried over anhydrous Na_2SO_4 and concentrated under vacuum, giving 68% yield of the pure product. ^{1}H NMR (500 MHz, $CDCl_3$) δ 7.39 – 7.30 (m, 4H), 7.10 (tt, *J* = 7.5, 1.1 Hz, 1H), 7.04 – 6.96 (m, 4H), 4.90 (qd, *J* = 6.4, 3.2 Hz,

1H), 1.77 (d, J = 3.5 Hz, 1H), 1.51 (d, J = 6.5 Hz, 3H). Spectral data are in accordance with those previously reported.[13]

2-phenoxyphenol: This compound was prepared following a literature procedure with modifications.[14] A 250 mL round bottom flask equipped with water condenser was charged with 1.44 g of 1-methoxy-2-phenoxybenzene, 24 mL of HBr (48%) solution and 48 mL of glacial acetic acid. The solution mixture was stirred at reflux temperature for 24 hours and allowed to cool to room temperature. The reaction mixture was extracted with 2x60 mL of dichloromethane, and the combined organic layer was washed with DI water (2x50 mL), then dried over anhydrous Na_2SO_4, and concentrated under vacuum. Both thin layer chromatography and ^{1}H NMR indicated no further purification was needed, which given 1.07 g of 2-phenoxyphenol (82% yield). ^{1}H NMR (500 MHz, $CDCl_3$) δ 7.40 – 7.32 (m, 2H), 7.14 (tt, J = 7.5, 1.2 Hz, 1H), 7.09 – 7.01 (m, 4H), 6.92 – 6.81 (m, 2H), 5.58 (s, 1H). Spectral data are in accordance with those previously reported.[15]

4,4'-oxybis(methoxybenzene):This compound was prepared following a literature procedure with modifications.[5] A 250 mL round bottom flask equipped with water condenser was charged with mixture of 12.8 mmol of 4-bromoanisole, 17.9 mmol of 4-methoxyphenol (1.5 equiv), 0.97 mmol of N,N-dimethylglycine (8 mol%), 2.1 mmol of CuI (16 mol%), 27.5 mmol of Cs_2CO_3 (2.15 equiv) and 100 mL of 1,4-dioxane. The reaction mixture was held at reflux temperature of 1,4-dioxane for 24 hours under nitrogen atmosphere. Upon the completion, the cooled reaction mixtures were filtered to remove the inorganic salt, the filtrate was then poured into 100 mL of DI water and extracted with 2x100 mL of dichloromethane. The combined organic layers were extracted with 2x50 mL of 0.6 M NaOH solution to wash out the excess 4-methoxyphenol, then washed with brine, dried using Na_2SO_4 and concentrated under vacuum. The crude product was purified by column chromatography using DCM/Hexanes (4:1 to 100% DCM) to give 1.26 g of 4,4'-oxybis(methoxybenzene) in 43% yield. ^{1}H NMR (500 MHz, $CDCl_3$) δ 6.92 (d, J = 9.1 Hz, 4H), 6.85 (d, J = 9.1 Hz, 4H), 3.79 (s, 6H). Spectral data are in accordance with those previously reported.[16]

Supplementary Figure 71. ^{1}H NMR (500 MHz) of **4-phenoxytoluene** measured in $CDCl_3$ at room temperature.

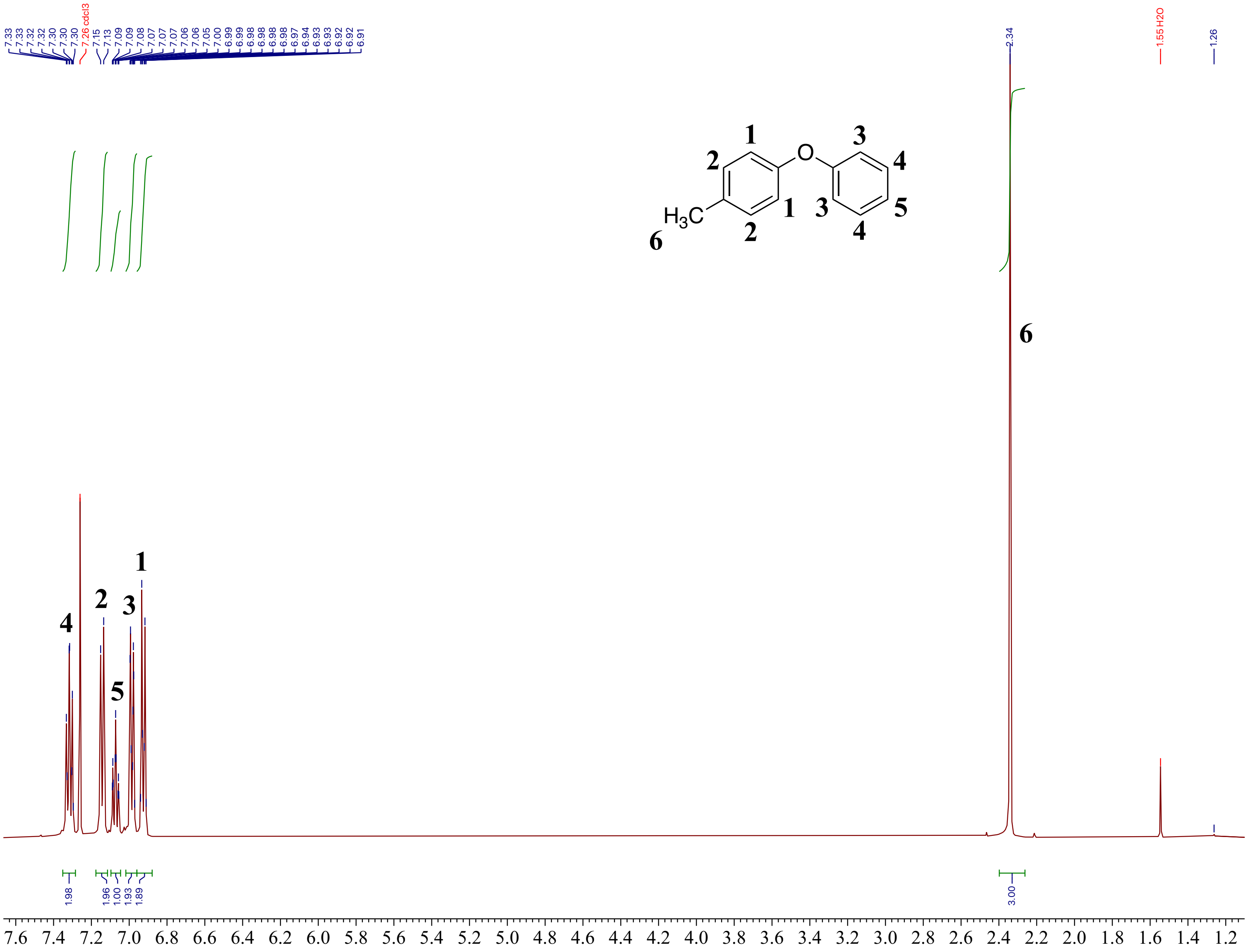

Supplementary Figure 72. ^{1}H NMR (500 MHz) of **3-phenoxytoluene** measured in $CDCl_3$ at room temperature.

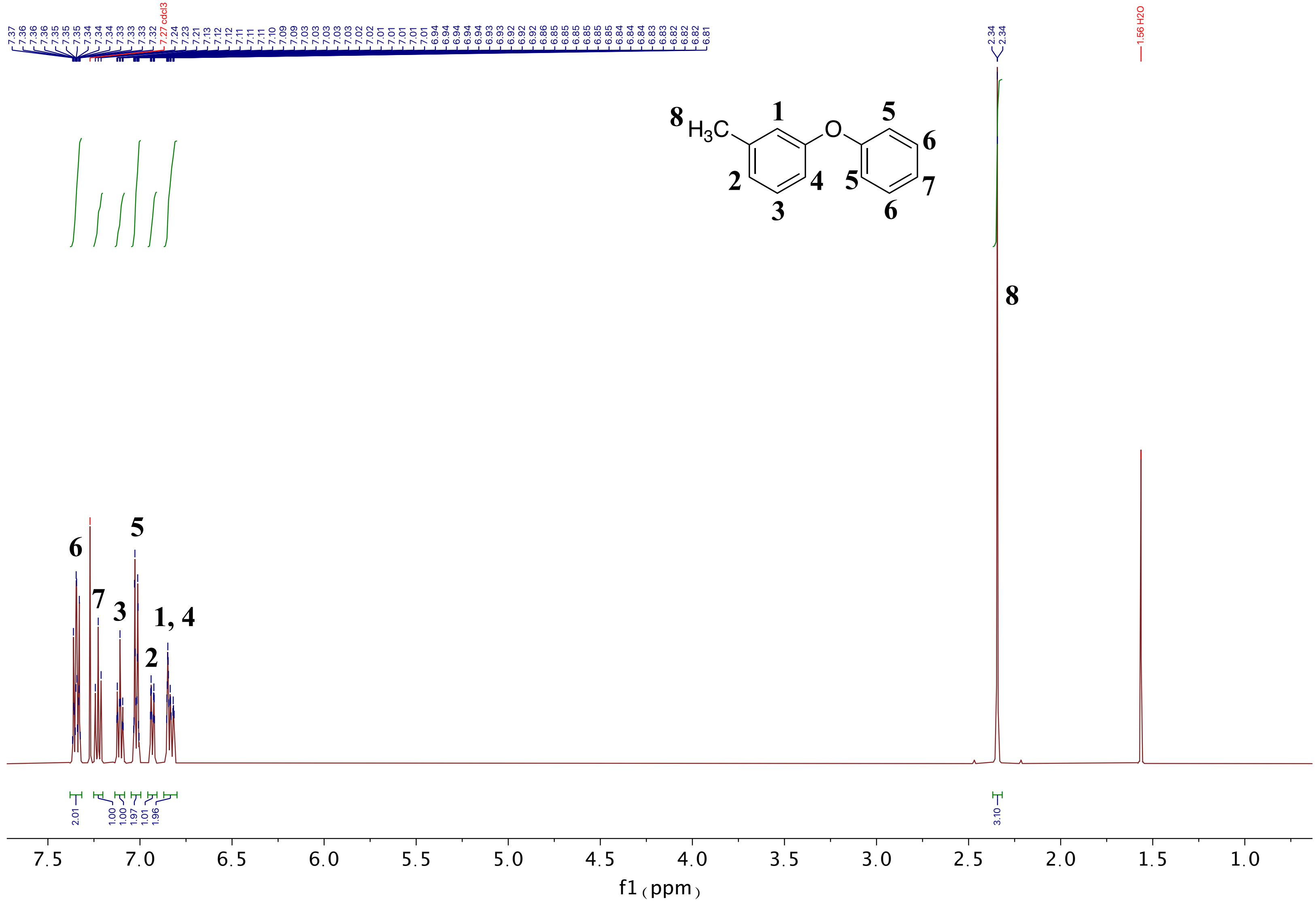

Supplementary Figure 73. ^{1}H NMR (500 MHz) of **2-phenoxytoluene** measured in $CDCl_3$ at room temperature.

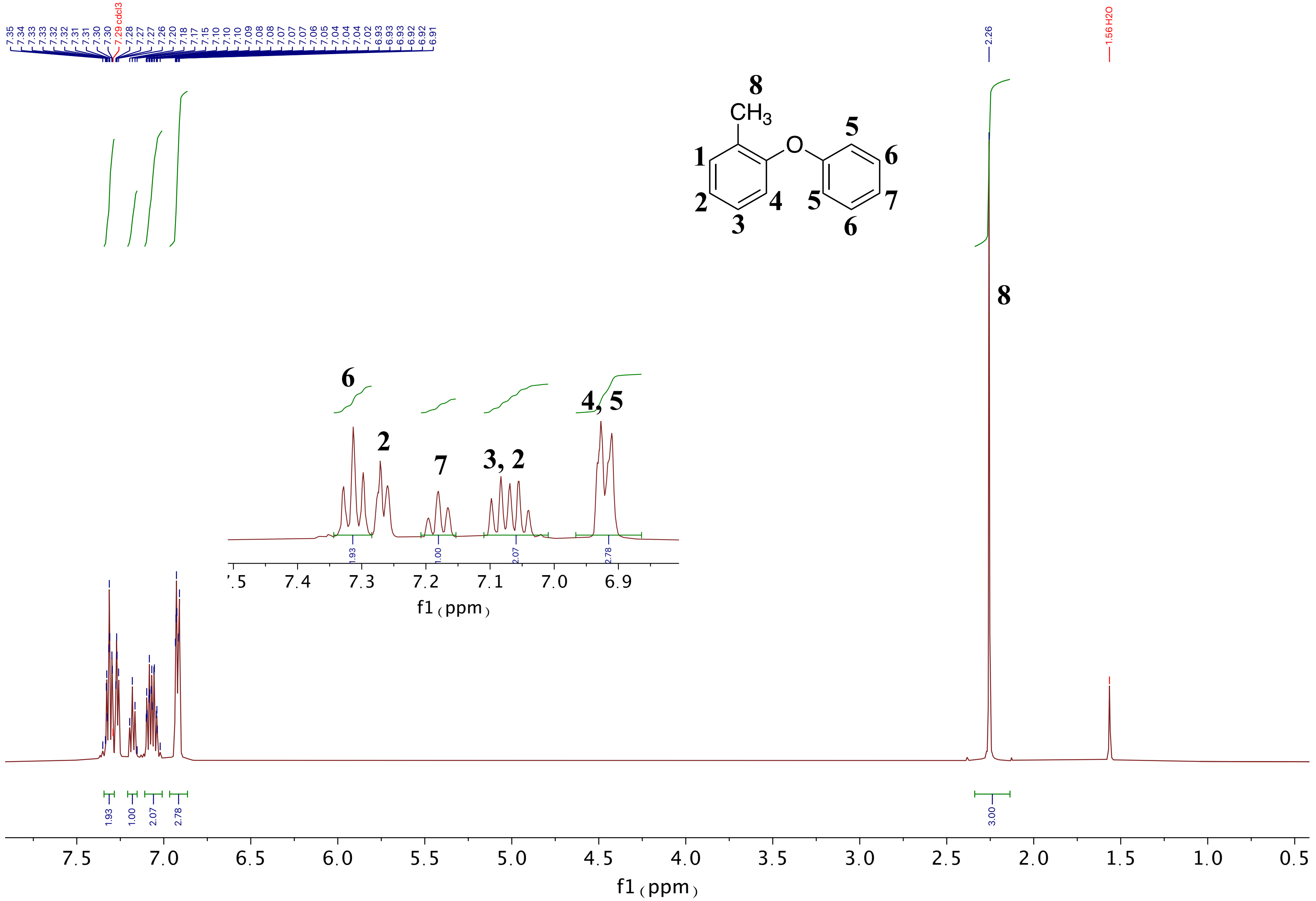

Supplementary Figure 74. ^{1}H NMR (500 MHz) of **4-phenoxyanisole** measured in $CDCl_3$ at room temperature.

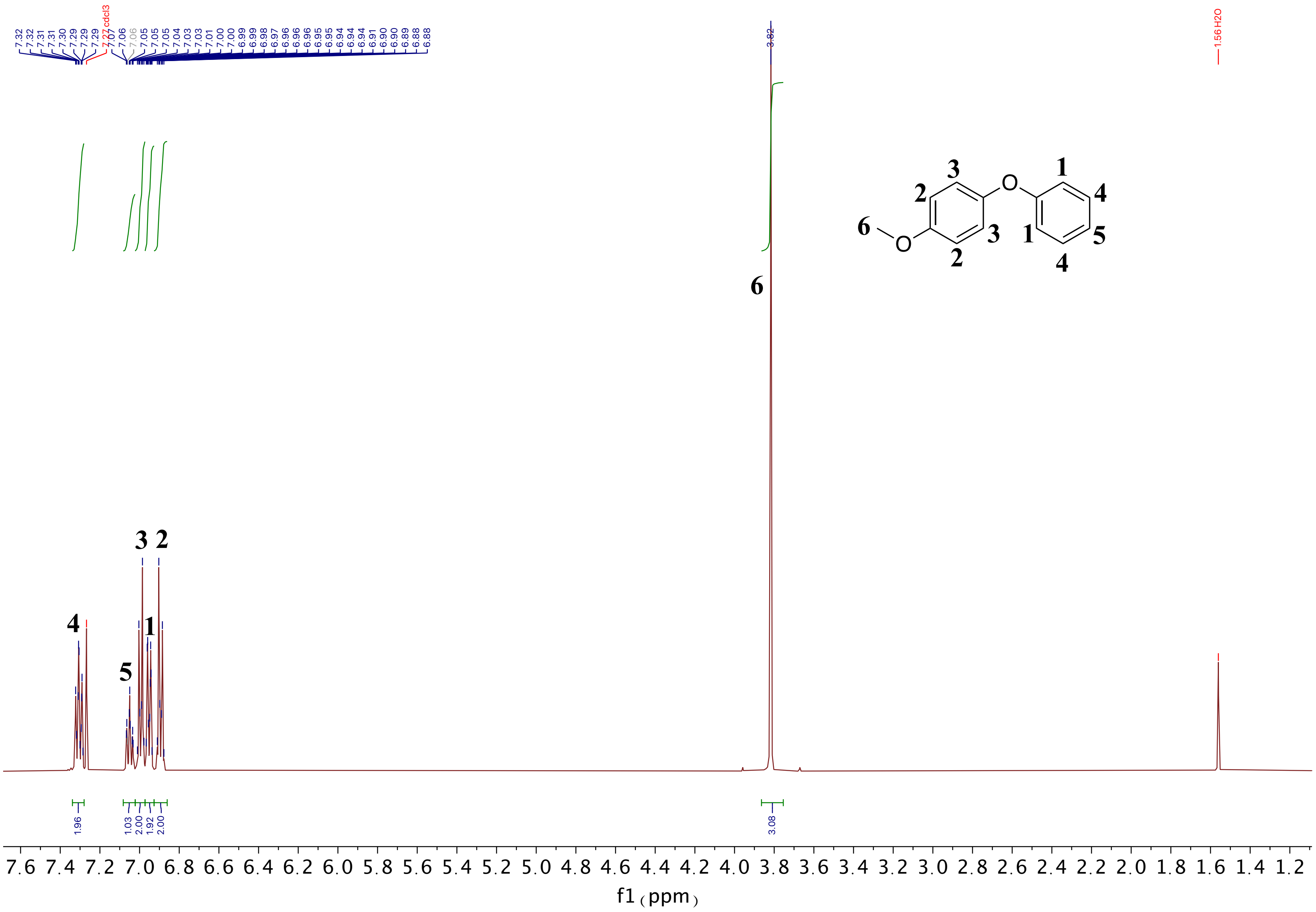

Supplementary Figure 75. ^{1}H NMR (500 MHz) of **3-phenoxyanisole** measured in $CDCl_3$ at room temperature.

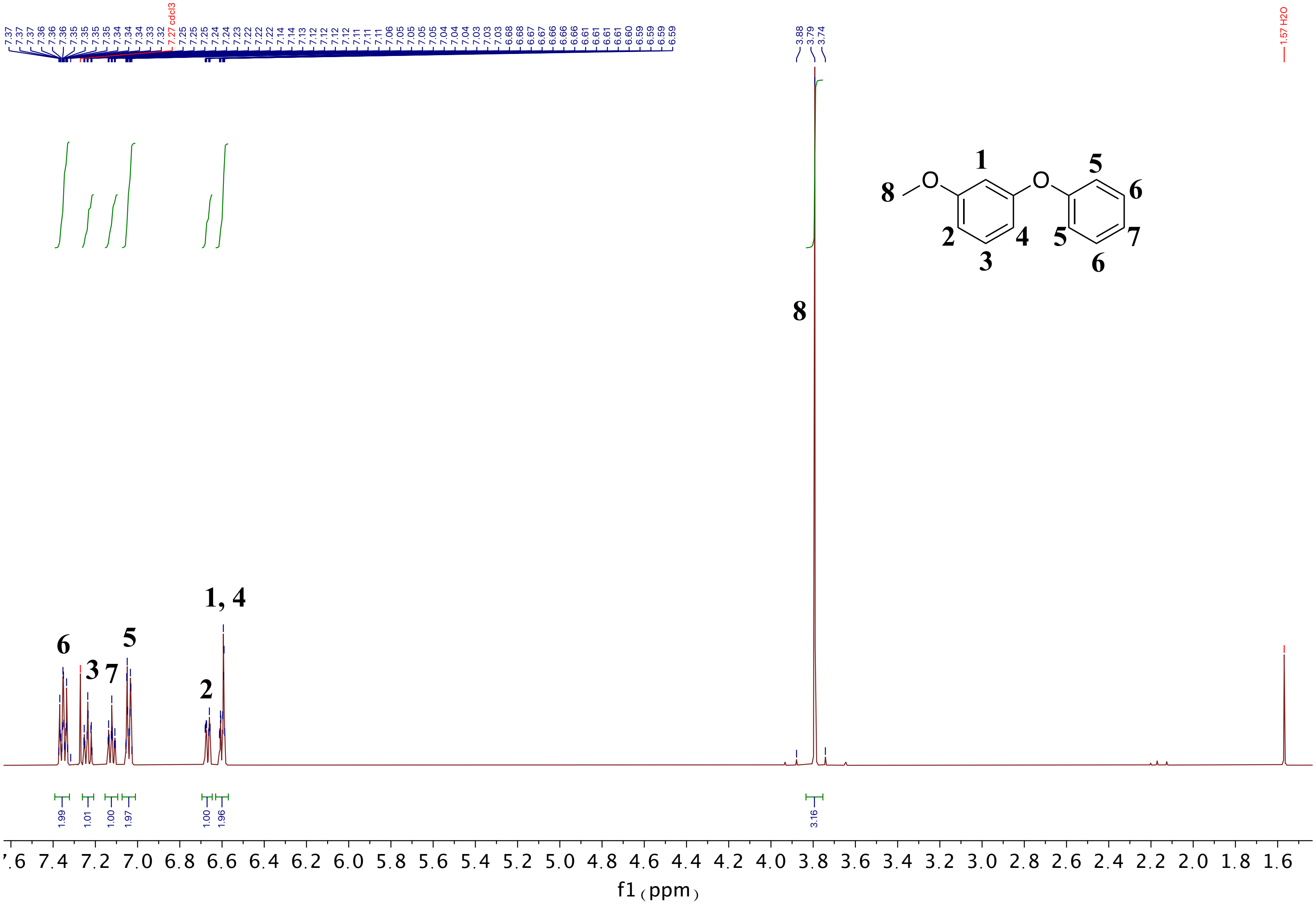

Supplementary Figure 76. ^{1}H NMR (500 MHz) of **2-phenoxyanisole** measured in $CDCl_3$ at room temperature.

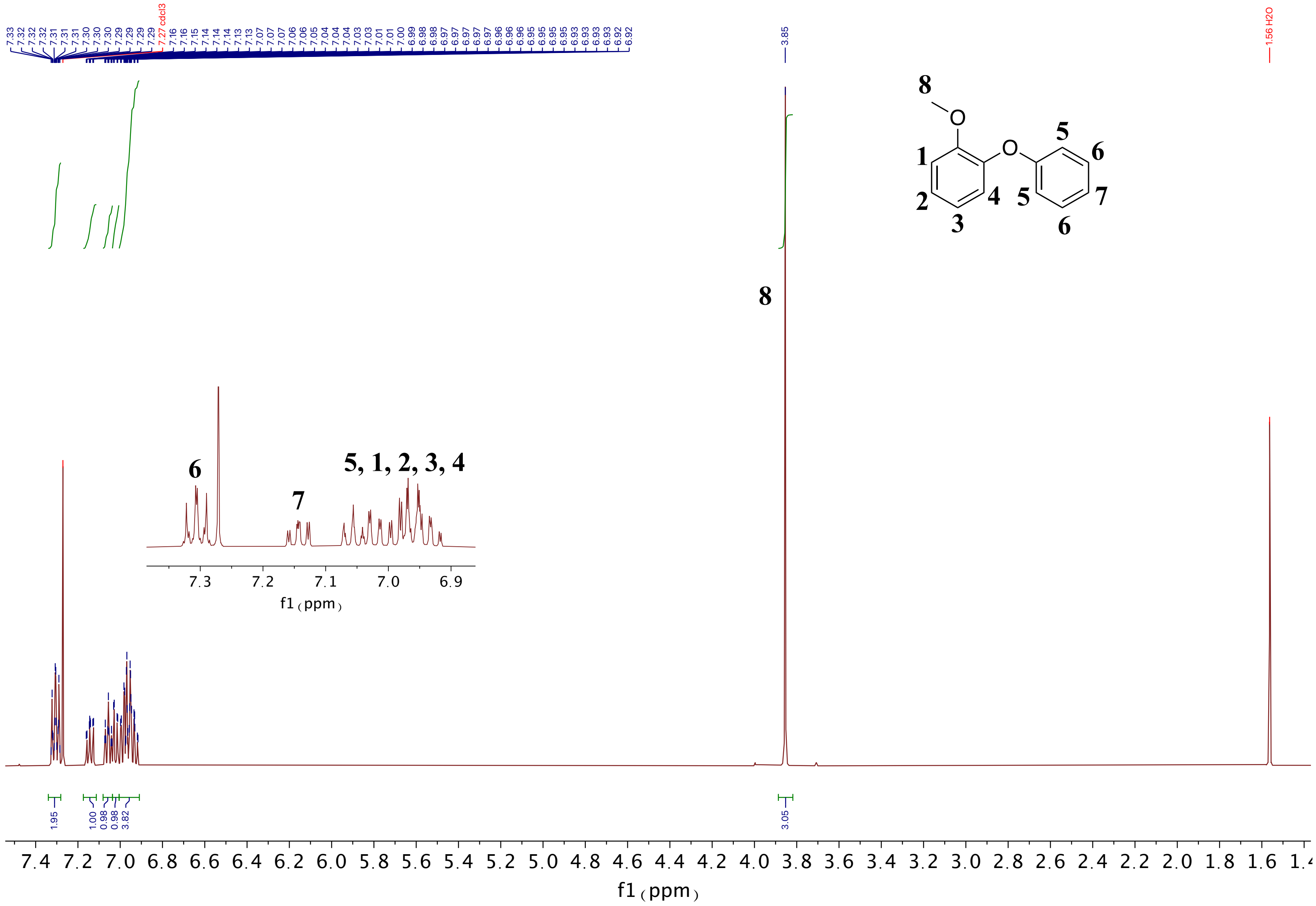

Supplementary Figure 77. ^{1}H NMR (500 MHz) of **1-fluoro-4-phenoxybenzene** measured in $CDCl_3$ at room temperature.

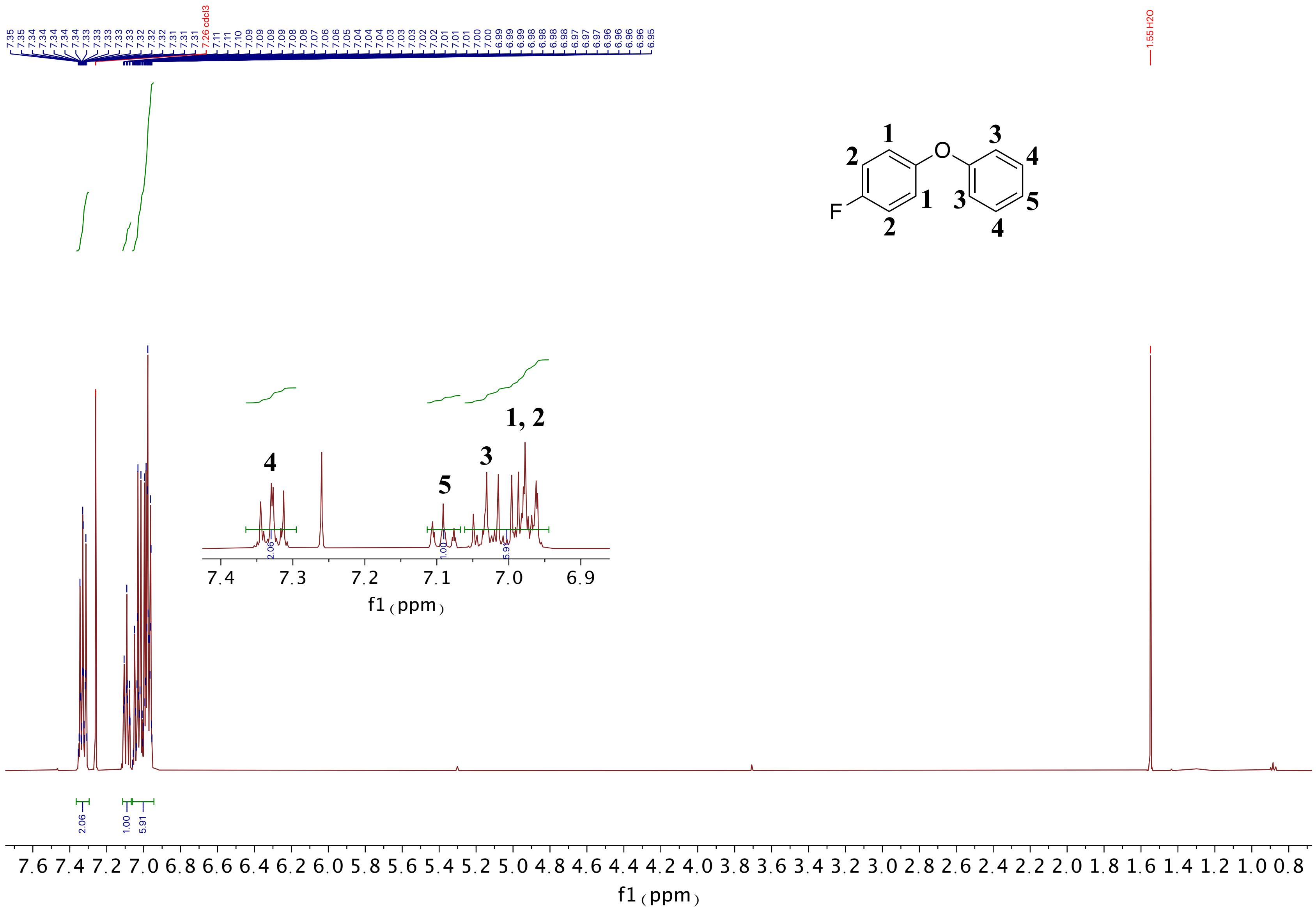

Supplementary Figure 78. ^{1}H NMR (500 MHz) of **4-phenoxybenzonitrile** measured in $CDCl_3$ at room temperature.

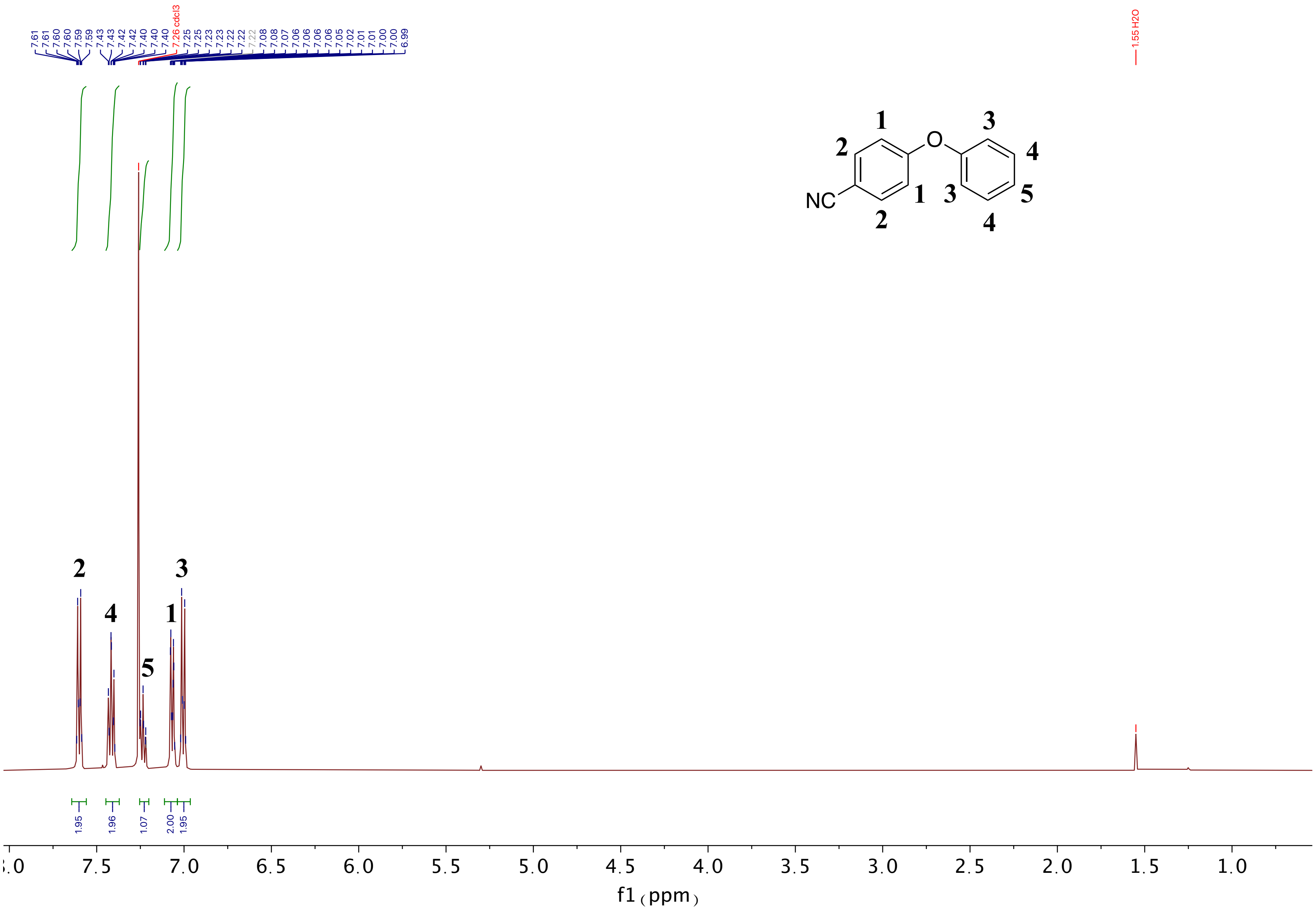

Supplementary Figure 79. ^{1}H NMR (500 MHz) of **1-phenoxy-4-(trifluoromethyl)benzene** measured in $CDCl_3$ at room temperature.

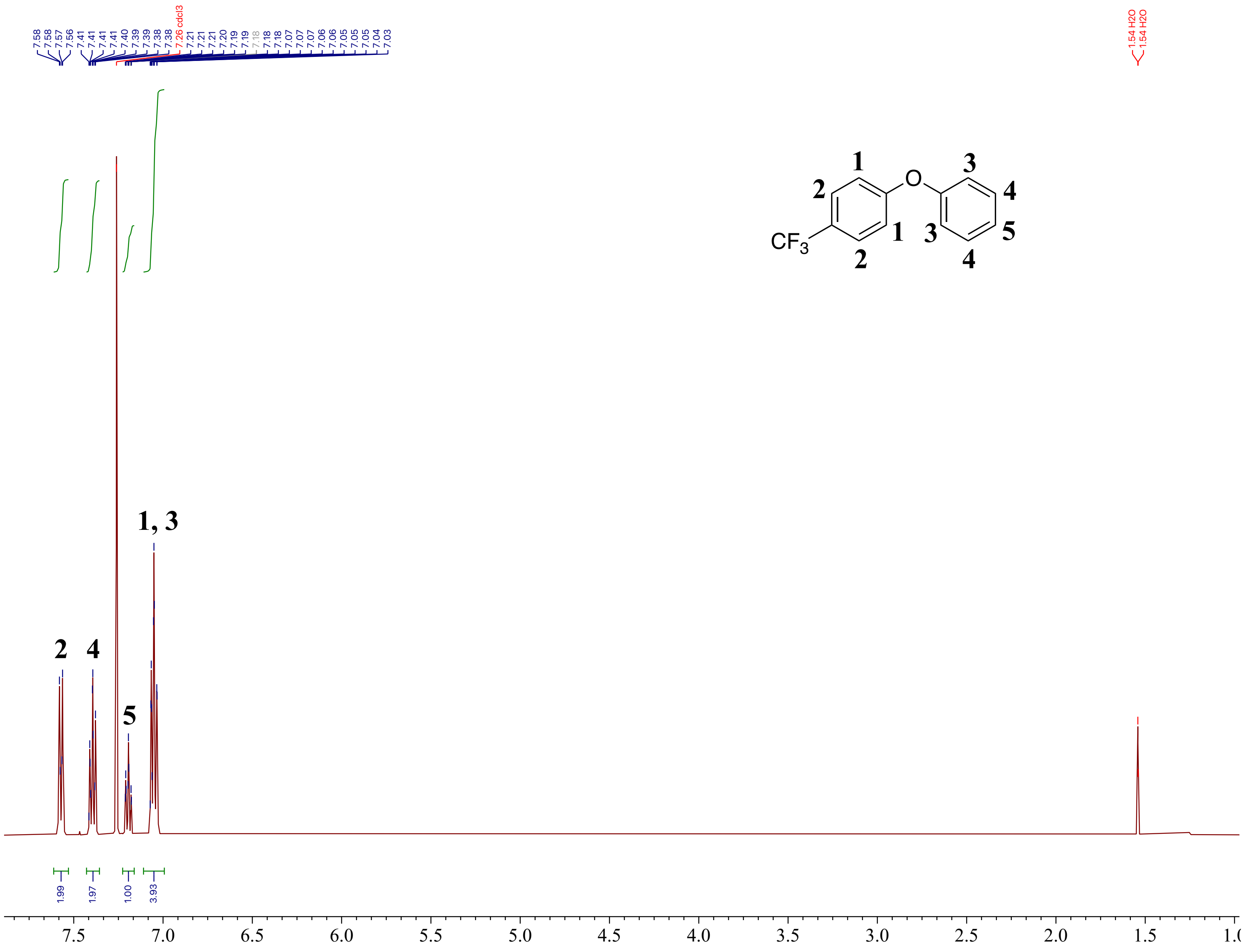

Supplementary Figure 80. ^{1}H NMR (500 MHz) of **4-phenoxybenzaldehyde** measured in $CDCl_3$ at room temperature.

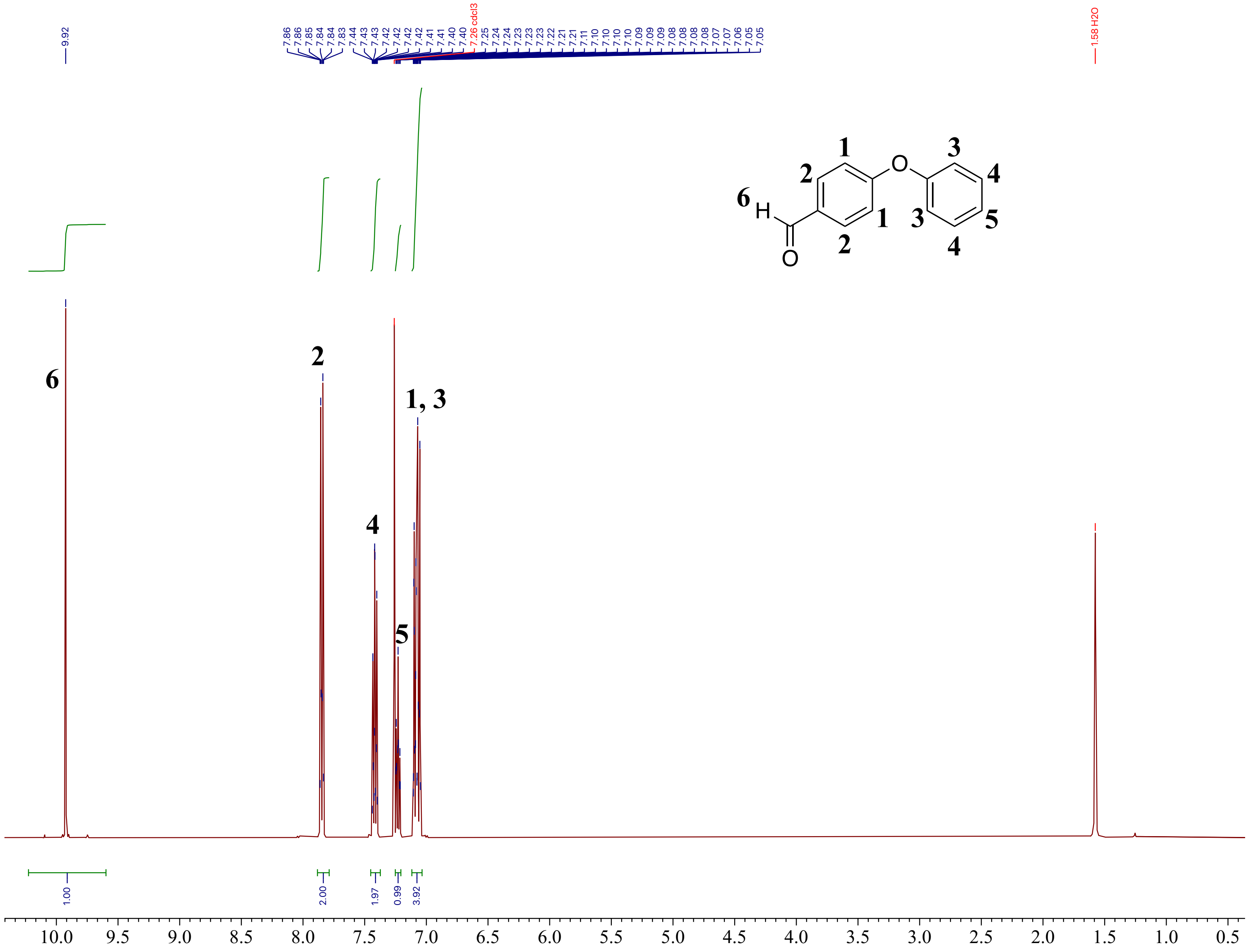

Supplementary Figure 81. ^{1}H NMR (500 MHz) of **1-(4-phenoxyphenyl)ethan-1-one** measured in $CDCl_3$ at room temperature.

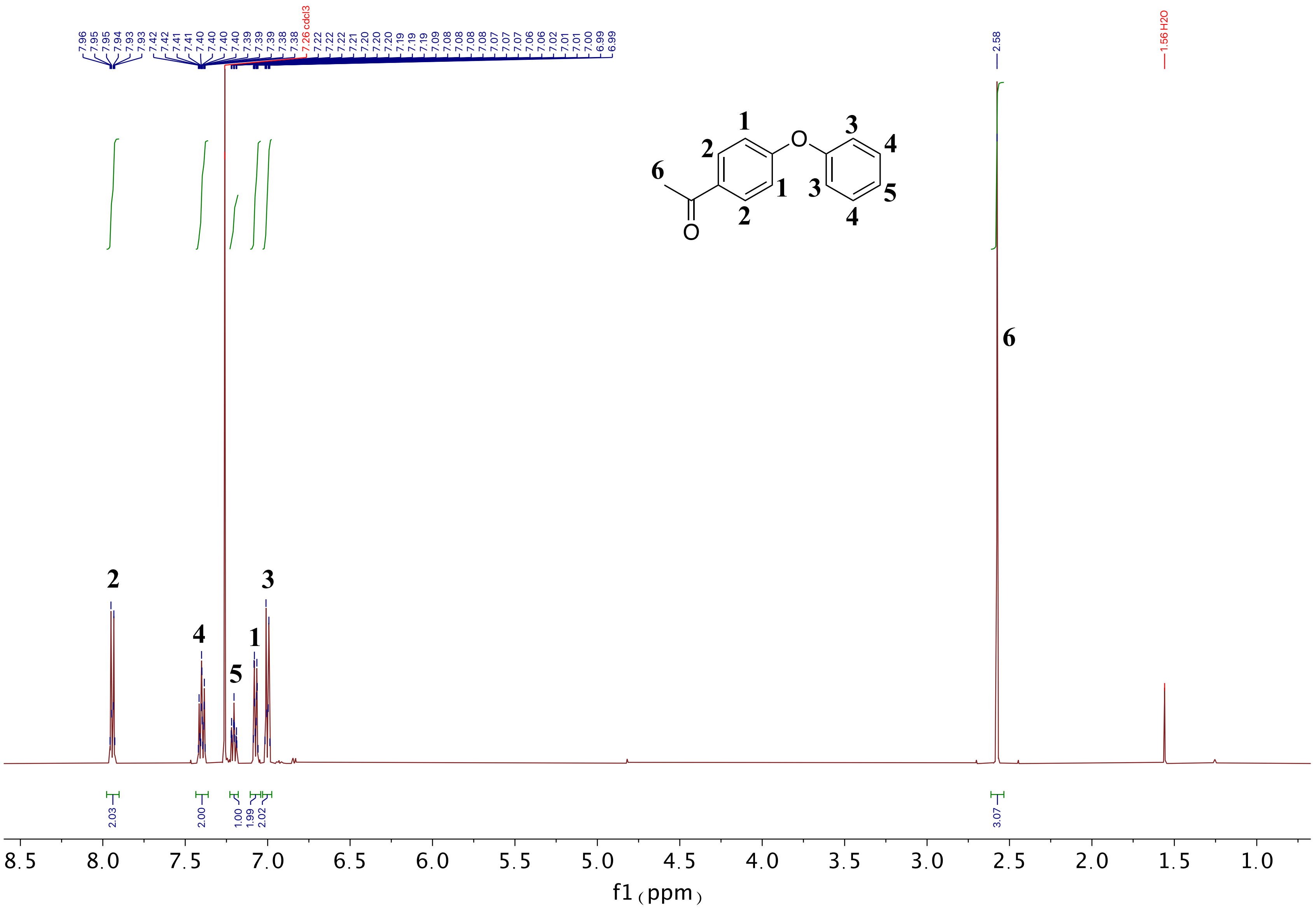

Supplementary Figure 82. ^{1}H NMR (500 MHz) of **1-ethyl-4-phenoxybenzene** measured in $CDCl_3$ at room temperature.

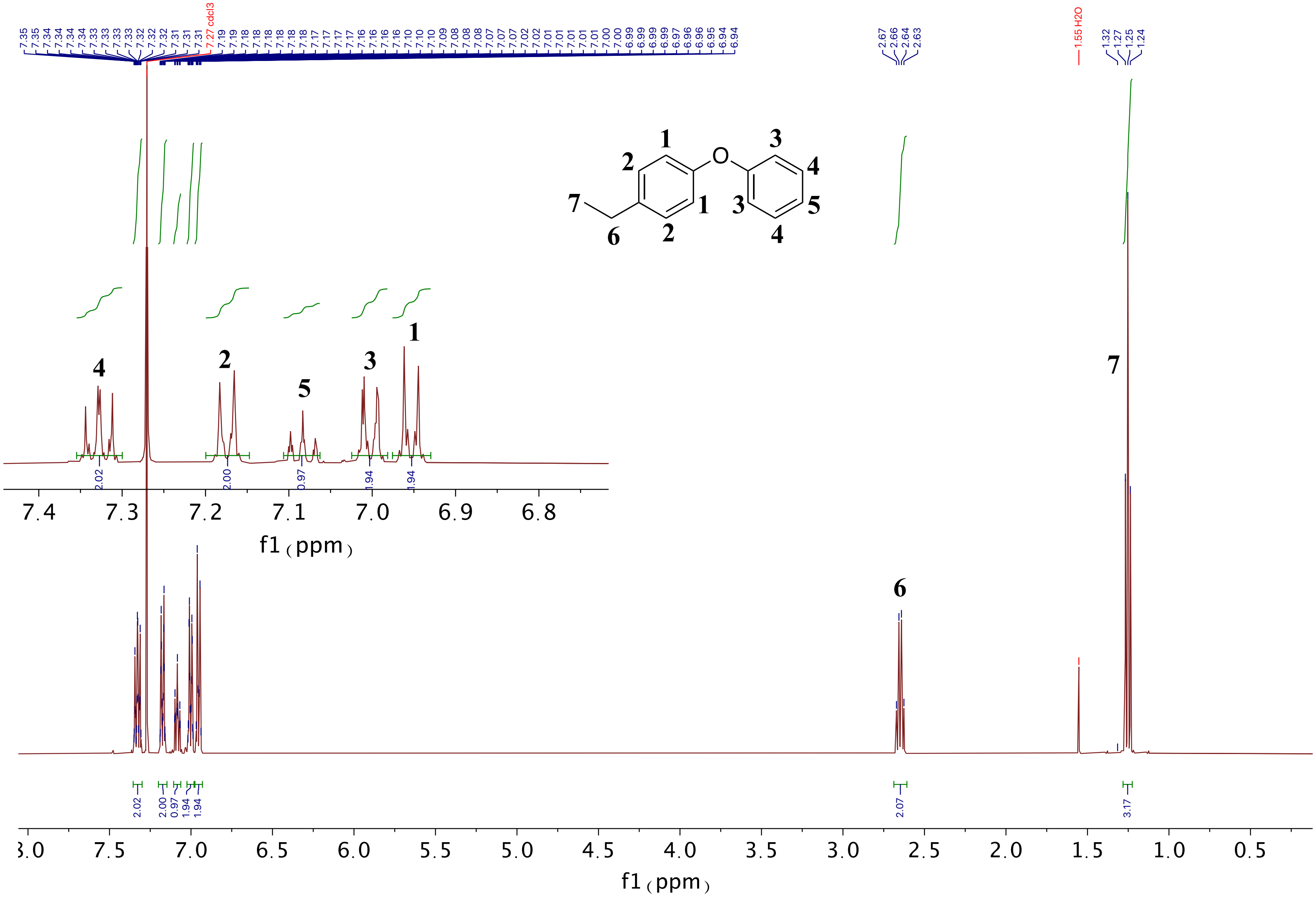

Supplementary Figure 83. ^{1}H NMR (500 MHz) of **1-(4-phenoxyphenyl)ethan-1-ol** measured in $CDCl_3$ at room temperature.

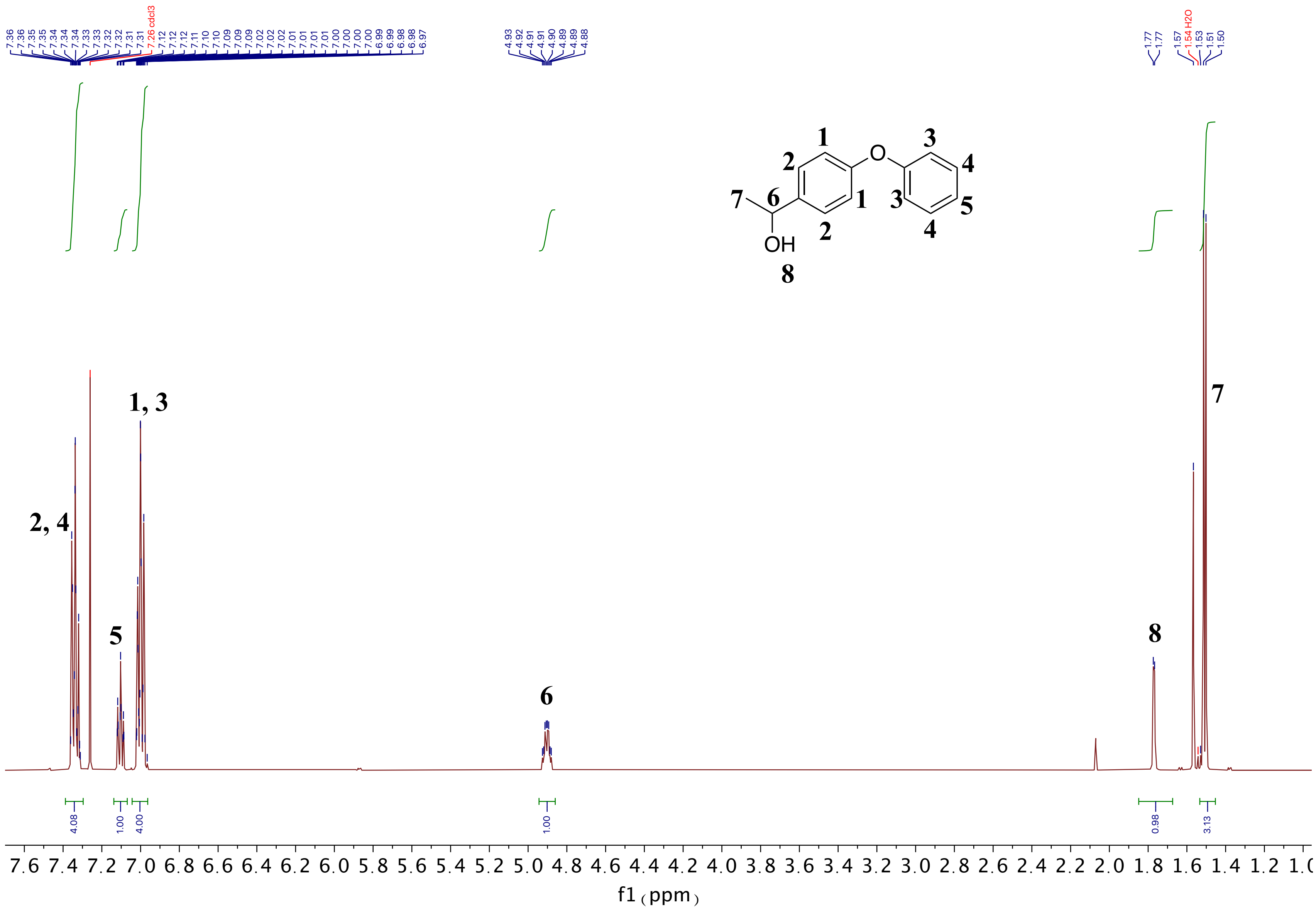

Supplementary Figure 84. ^{1}H NMR (500 MHz) of **2-phenoxyphenol** measured in $CDCl_3$ at room temperature.

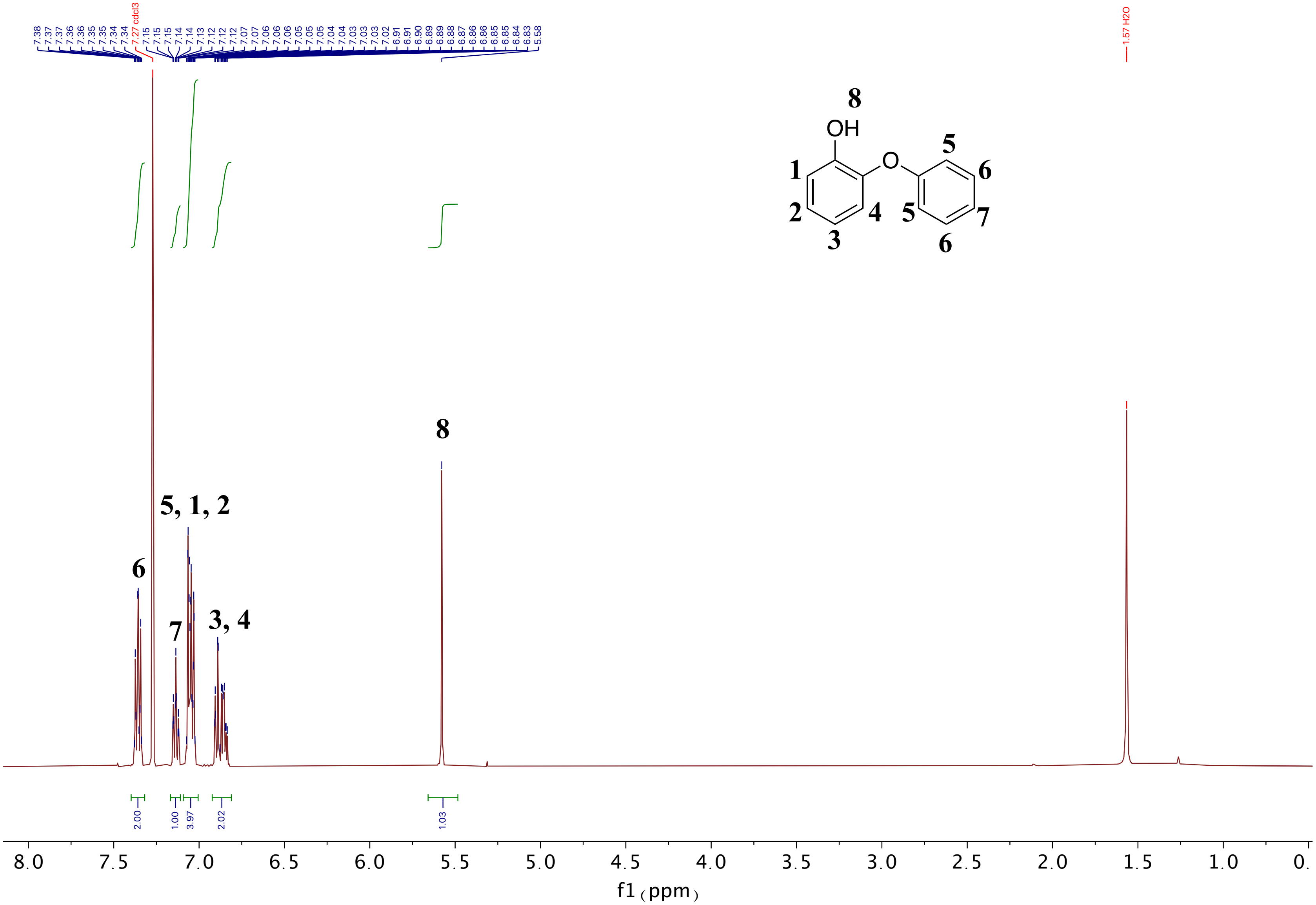

Supplementary Figure 85. ^{1}H NMR (500 MHz) of **4,4'-oxybis(methoxybenzene)** measured in $CDCl_3$ at room temperature.

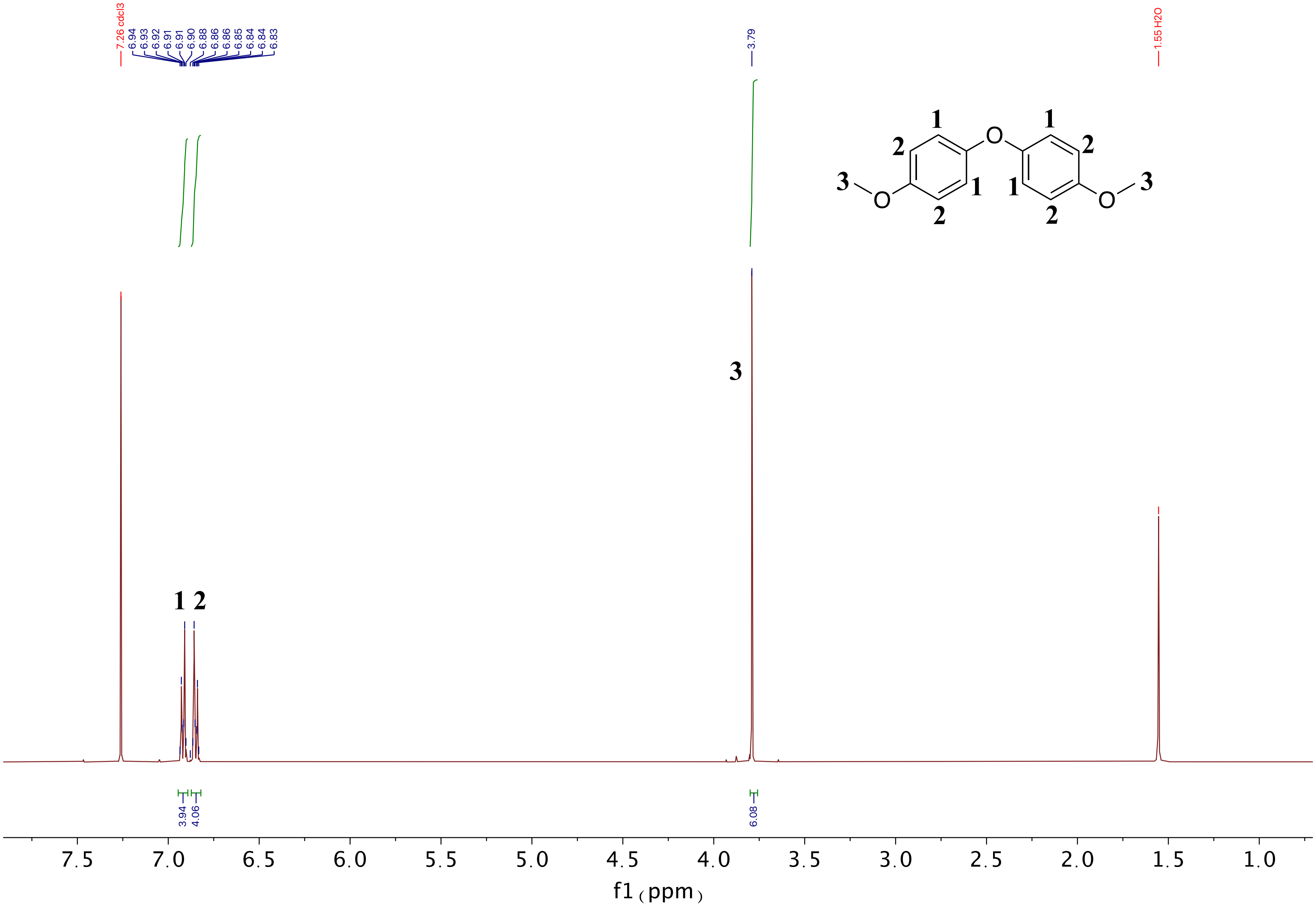

Supplementary References

1. Lam, C. H.; Lowe, C. B.; Li, Z. L.; Longe, K. N.; Rayburn, J. T.; Caldwell, M. A.; Houdek, C. E.; Maguire, J. B.; Saffron, C. M.; Miller, D. J.; Jackson, J. E., Electrocatalytic upgrading of model lignin monomers with earth abundant metal electrodes. *Green Chemistry* **2015,** *17* (1), 601-609.

2. Zhou, Y. T.; Klinger, G. E.; Hegg, E. L.; Saffron, C. M.; Jackson, J. E., Multiple Mechanisms Mapped in Aryl Alkyl Ether Cleavage via Aqueous Electrocatalytic Hydrogenation over Skeletal Nickel. *Journal of the American Chemical Society* **2020,** *142* (8), 4037-4050.

3. Kanan, M. W.; Nocera, D. G., In situ formation of an oxygen-evolving catalyst in neutral water containing phosphate and Co2+. *Science* **2008,** *321* (5892), 1072-1075.

4. Lin, C. Y.; George, M. W.; Gill, P. M. W., EDF2: A density functional for predicting molecular vibrational frequencies. *Australian Journal of Chemistry* **2004,** *57* (4), 365-370.

5. Ma, D. W.; Cai, Q., N,N-Dimethyl glycine-promoted Ullmann coupling reaction of phenols and aryl halides. *Organic Letters* **2003,** *5* (21), 3799-3802.

6. Hu, T. J.; Chen, X. R.; Wang, J.; Huang, J., CuO Nanoparticles on Ionic Polymer for Coupling Reactions of Aryl Bromides and Chlorides with Phenols. *Chemcatchem* **2011,** *3* (4), 661-665.

7. Burgos, C. H.; Barder, T. E.; Huang, X. H.; Buchwald, S. L., Significantly improved method for the Pd-catalyzed coupling of phenols with aryl halides: Understanding ligand effects. *Angewandte Chemie-International Edition* **2006,** *45* (26), 4321-4326.

8. Phan, N. T. S.; Nguyen, T. T.; Nguyen, V. T.; Nguyen, K. D., Ligand-Free Copper-Catalyzed Coupling of Phenols with Nitroarenes by using a Metal-Organic Framework as a Robust and Recoverable Catalyst. *Chemcatchem* **2013,** *5* (8), 2374-2381.

9. Zhang, R. Z.; Liu, J. M.; Wang, S. F.; Niu, J. Z.; Xia, C. G.; Sun, W., Magnetic CuFe2O4 Nanoparticles as an Efficient Catalyst for C-O Cross-Coupling of Phenols with Aryl Halides. *Chemcatchem* **2011,** *3* (1), 146-149.

10. Zhang, S. L.; Bie, W. F., Isolation and characterization of copper(III) trifluoromethyl complexes and reactivity studies of aerobic trifluoromethylation of arylboronic acids. *Rsc Advances* **2016,** *6* (75), 70902-70906.

11. Zhang, J. L.; Chen, J. X.; Liu, M. C.; Zheng, X. W.; Ding, J. C.; Wu, H. Y., Ligand-free copper-catalyzed coupling of nitroarenes with arylboronic acids. *Green Chemistry* **2012,** *14* (4), 912-916.

12. Dawange, M.; Galkin, M. V.; Samec, J. S. M., Selective Aerobic Benzylic Alcohol Oxidation of Lignin Model Compounds: Route to Aryl Ketones. *Chemcatchem* **2015,** *7* (3), 401-404.

13. Furuta, A.; Nishiyama, H., Highly efficient catalytic system for hydrosilylation of ketones with iron(II) acetate-thiophenecarboxylate. *Tetrahedron Letters* **2008,** *49* (1), 110-113.

14. Dingemans, T. J.; Mendes, E.; Hinkley, J. J.; Weiser, E. S.; StClair, T. L., Poly(ether imide)s from diamines with para-, meta-, and ortho-arylene substitutions: Synthesis, characterization, and liquid crystalline properties. *Macromolecules* **2008,** *41* (7), 2474-2483.

15. Gogoi, P.; Bezboruah, P.; Gogoi, J.; Boruah, R. C., ipso-Hydroxylation of Arylboronic Acids and Boronate Esters by Using Sodium Chlorite as an Oxidant in Water. *European Journal of Organic Chemistry* **2013,** *2013* (32), 7291-7294.

16. Ramezani, L.; Yahyazadeh, A.; Sheykhan, M., The First C-Cl Activation in Ullmann C-O Coupling by MOFs. *Chemcatchem* **2018,** *10* (20), 4636-4651.
